# A Study to Investigate the Efficacy and Safety of an Anti-Interleukin-18 Monoclonal Antibody in the Treatment of Type 2 Diabetes Mellitus

**DOI:** 10.1371/journal.pone.0150018

**Published:** 2016-03-01

**Authors:** Elizabeth A. McKie, Juliet L. Reid, Prafull C. Mistry, Stephen L. DeWall, Lee Abberley, Philip D. Ambery, Blas Gil-Extremera

**Affiliations:** 1 Respiratory R&D, GlaxoSmithKline, Uxbridge, United Kingdom; 2 Immunoinflammation Therapy Area Unit, GlaxoSmithKline, Stevenage, United Kingdom; 3 Clinical Statistics, GlaxoSmithKline, Stevenage, United Kingdom; 4 Clinical Immunology, Biopharm R&D, GlaxoSmithKline, King of Prussia, Pennsylvania, United States of America; 5 Drug Metabolism and Pharmacokinetics, GlaxoSmithKline, King of Prussia, Pennsylvania, United States of America; 6 GlaxoSmithKline, Clinical development, Cardiovascular and Metabolic Medicines Development Centre, London, United Kingdom; 7 Department of Medicine, University Hospital San Cecilio, Granada, Spain; Rush University, UNITED STATES

## Abstract

**Objective:**

Evidence suggests that chronic subclinical inflammation plays an important role in the pathogenesis of type 2 diabetes (T2DM). Circulating levels of interleukin (IL)-18 appear to be associated with a number of micro- and macrovascular comorbidities of obesity and T2DM. This study was designed to investigate whether inhibition of IL-18 had any therapeutic benefit in the treatment of T2DM. Preliminary efficacy, safety and tolerability, pharmacokinetics, and pharmacodynamics of the anti-IL-18 monoclonal antibody, GSK1070806, were assessed.

**Research Design and Methods:**

This was a multicentre, randomized, single-blind (sponsor-unblinded), placebo-controlled, parallel-group, phase IIa trial. Obese patients of either sex, aged 18–70 years, with poorly controlled T2DM on metformin monotherapy were recruited. Patients received two doses, of placebo (n = 12), GSK1070806 0.25 mg/kg (n = 13) or GSK1070806 5 mg/kg (n = 12). The primary end-point was the change from baseline in fasting plasma glucose and weighted mean glucose area under the curve (AUC)(0–4 hours) postmixed meal test on Days 29, 57, and 85.

**Results:**

Thirty-seven patients were randomized to one of the three treatment arms. There were no statistically significant effects of GSK1070806 doses on fasting plasma glucose levels, or weighted mean glucose AUC(0–4 hours) compared with placebo.

**Conclusions:**

GSK1070806 was well tolerated, and inhibition of IL-18 did not lead to any improvements in glucose control. However, because of study limitations, smaller, potentially clinically meaningful effects of IL-18 inhibition cannot be excluded.

**Trial Registration:**

ClinicalTrials.gov NCT01648153

## Introduction

Type 2 diabetes mellitus (T2DM), is a heterogeneous disorder characterized by multiple defects in insulin action in tissues (muscle, adipose, and liver) and defects in pancreatic insulin secretion; this eventually leads to loss of pancreatic insulin-secreting cells. The treatment goals for T2DM patients are effective control of blood glucose, blood pressure, and lipids (if elevated), and, ultimately, to avert the serious complications associated with sustained tissue exposure to excessively high glucose concentrations. T2DM is associated with micro- and macrovascular complications, including cardiovascular disease, peripheral vascular disease, stroke, diabetic neuropathy, amputations, renal failure, and blindness that result in increasing disability and reduced life expectancy [1]. With progression of T2DM over time, multiple drugs, including insulin, are usually required to achieve acceptable glycemic control. Therefore, there is a need for new potentially disease-modifying treatments.

A growing body of evidence suggests that chronic subclinical inflammation plays an important role in the pathogenesis of T2DM. Elevated concentrations of various inflammatory markers in the circulation have been reported in humans with insulin resistance (IR), e.g., interleukin (IL)-1β [2], IL-6 [3], tumour necrosis factor (TNF)-α [4], soluble TNF receptors (sTNFR1, sTNFR2) [5], and C-reactive protein (CRP) [6]. In patients with T2DM, adipose tissue contributes significantly to the levels of proinflammatory cytokines in the circulation.

IL-18 is a member of the IL-1 family of cytokines. In addition to its role in the inflammatory response to microbes, recent studies have elucidated a broad spectrum of effector functions that implicate IL-18 as an important factor in human autoimmune and metabolic diseases. Studies in humans demonstrate elevated IL-18 levels in serum, adipose tissue, and muscle in obese patients with IR and patients with T2DM or metabolic syndrome [7–13]. The correlation between IL-18 levels and IR in T2DM patients appears to be independent of TNF-α, CRP, and IL-6 levels. In contrast to TNF-α and IL-6, IL-18 levels also correlate with early signs of IR in nondiabetic “healthy” individuals [3,9].

The observation that obese/diabetic individuals exhibit elevated levels of proinflammatory cytokines lends support to the hypothesis that obesity-induced IR is an inflammatory condition [14,15] and that inflammation, IR, and aberrant lipid metabolism may be interlinked components of the metabolic syndrome [16]. The link between the action of cytokines, such as IL-18, and IR has a mechanistic basis involving the activation of c-Jun N-terminal kinase 1(JNK1)/signal transducer and activator of transcription (STAT) kinase pathway and phosphorylation of the serine/threonine IRS1 [17], which is required for efficient signalling of insulin through its cognate receptor. In addition, circulating levels of IL-18 appear to be associated with a number of microvascular [18–22] and macrovascular [23–28] comorbidities of obesity and T2DM. Several lines of evidence implicate IL-18 in the direct induction of renal injury in diabetic nephropathy [18]. Patients with T2DM have not only a significantly higher serum but also higher urinary levels of IL-18 compared with healthy controls [19,20,29]. Moreover, there is a positive correlation between IL-18 levels in diabetic patients and the development of urinary albumin excretion, with the highest IL-18 levels found in patients with microalbuminuria and clinical albuminuria [19,20,29]. These observations suggest that treatment strategies that reduce inflammatory cytokines such as anti-IL-18 could potentially be disease modifying in the treatment of diabetes.

GSK1070806 is a humanized IgG1 antibody that binds to human IL-18 with a high affinity (K_d_ = 30.3 pM) and neutralizes its function. In the first time in human (FTIH) study [30], single intravenous (i.v.) infusions of GSK1070806 at doses ≤10 mg/kg in healthy patients and ≤3 mg/kg in obese patients were well tolerated. The current study was designed to investigate the preliminary efficacy, safety, and tolerability of two i.v. doses of the anti-IL-18 monoclonal antibody, GSK1070806, in patients with T2DM.

## Research Design and Methods

### Setting and participants

Obese male or female patients of nonchildbearing potential between 18 and 70 years of age inclusive, at the time of signing the informed consent and with a body mass index ≥30 kg/m^2^ and <40 kg/m^2^, with T2DM were eligible for inclusion in the study. Only women of nonchildbearing potential were eligible for the study, as the compound was in an early stage of development, and preclinical reprotoxicity studies had not yet been conducted. Patients were required to be on a stable dose of monotherapy with metformin for 3 months prior to screening and at a total daily dose ≥1,000 mg for at least 2 months prior to dosing. Patients were also required to have >7% HbA1c (53 mmol/mol) but ≤9.5% (80 mmol/mol) and levels of microalbuminuria indicative of progressive kidney disease, i.e., 30–300 mg/l albumin in urine or albumin creatinine ratio ≥3.5 mg/mmol (female) or ≥2.5 mg/mmol (male), and ≤30 mg/mmol (female and male).

Subjects who tested positive for *Mycobacterium tuberculosis*, hepatitis B, hepatitis C, or human immunodeficiency virus were excluded from the study. Other key exclusion criteria are listed in the S1 Protocol. The trial was performed after approval by the ethics committee East of England–Cambridge East Ethics at each center and according to good clinical practice and the Declaration of Helsinki 2008 (ClinicalTrials.gov ID: NCT01648153). Written informed consent was obtained from each patient prior to the performance of any study-specific procedures. The study period was 6 August 2012 to 3 January 2014. The authors confirm that all ongoing and related trials for this drug/intervention are registered.

### Trial design

**Fig 1:**
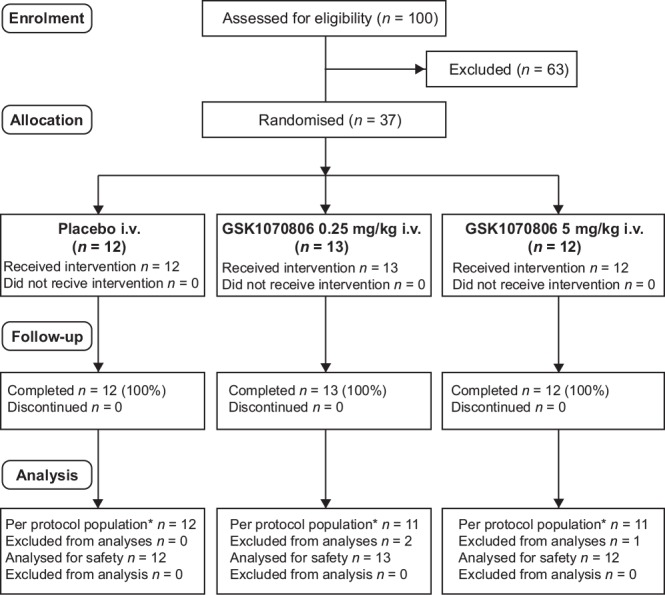
CONSORT Flow Diagram. *Per protocol population was used for applicable efficacy, PK, and PD/biomarker analyses.

This multicenter, randomized, single-blind (sponsor-unblinded), placebo-controlled, parallel-group, phase IIa trial was conducted at seven centers in Spain. Patients were randomized to placebo, 0.25 mg/kg GSK1070806, or 5 mg/kg GSK1070806, and received two i.v. infusions of GSK1070806 or placebo 28 days apart (Fig 1). A postmixed meal test (MMT) challenge (using Ensure-High Protein) was conducted on study Days 1 (predose), 29, 57, and 85 (S1 Fig). Blood samples for the measurement of glucose, insulin, and C-peptide were taken immediately before the MMT (within 5 min), and at 15, 30, 60, 90, 120, 180, and 240 min following ingestion of the Ensure-High Protein.

The primary objective of the study was to assess the efficacy of two repeat i.v. administrations of GSK1070806 in obese patients with T2DM. This was assessed by measuring improvements in fasting and postprandial glucose control, specifically, the change from baseline in fasting plasma glucose (FPG) and weighted mean glucose area under the curve (AUC)(0–4 hours) post-MMT (calculated using the linear trapezoidal method) on Days 29, 57, and 85.

Safety and tolerability [adverse events (AEs), clinical laboratory tests, ECGs, and vital signs] were assessed at all clinic visits. Efficacy [change from baseline in percent HbA_1c_, fasting blood insulin, and C-peptide levels; change from baseline in weighted mean insulin and C-peptide levels AUC(0–4 hours) post-MMT] was measured on Days 29, 57, and 85; derived measures of insulin sensitivity and β-cell function were also calculated at these time points. Other secondary objectives included plasma pharmacokinetic (PK) parameters [AUC(0–τ), maximum serum concentration (C_max_), median time to reach C_max_ (T_max_) and after the second dose of λz and *t*_½_]; pharmacodynamic (PD) markers (serum levels of free IL-18 and drug-bound IL-18); and change from baseline in serum and/or plasma levels of biomarkers of inflammation of cardiovascular and metabolic disease [adiponectin, high-sensitivity (hs)-CRP, IP-10 (CXCL10), fructosamine, hs-IL-6, sICAM, MMP-9, NEFA, PAI-1, and resistin]. Further details can be found in S1 Protocol.

Patients were assigned equally to one of the three trial arms in accordance with the randomization schedule generated by GlaxoSmithKline’s Biopharm Clinical Pharmacology and Biometrics Department, prior to the start of the study, using validated internal software. Patients remained on their current diet and dose of metformin. This was a single-blind (sponsor-unblinded) study; patients, site staff, and site monitors were blinded, but the GlaxoSmithKline study team were unblinded.

### Statistical analysis

The sample size was selected using an estimation-based approach to sample-size derivation to allow preliminary characterization of efficacy, safety, and tolerability in obese patients with T2DM, and to investigate the effect on PD assessment and biomarkers. Since an estimation-based approach was used, no formal hypothesis was tested, and there were no formal calculations of power or sample size for this study. Expected 95% confidence interval widths for the mean difference in plasma glucose between treatment and placebo were calculated, assuming that 10 patients with evaluable data completed each active arm and the placebo group with an estimated (i.e., using internal GSK data) standard deviation for change from baseline in FPG and weighted mean glucose AUC(0–4 hours) of 27.0 and 32.8, respectively. Based on these assumptions, and using a two-sided two-sample t test, this study was estimated to provide 80% power to detect a difference in change from baseline FPG of 35.8 mg/dl (1.99 mmol/l) and a difference in change from baseline weighted mean AUC(0–4 hours) of 45.8 mg/dl (2.54 mmol/l). Although differences as low as 30 mg/dl (1.66 mmol/l) for both FPG and weighted mean AUC(0–4 hours) may be of clinical significance, the study was not powered to detect changes of this magnitude.

Full details of the statistical analysis can be found in the S1 Protocol.

## Results

### Patient characteristics and trial flow

The trial was conducted between August 2012 and January 2014. Thirty-seven patients were enrolled (Fig 1); all patients attended all study visits, and there were no withdrawals from the study. Baseline demographic characteristics are summarized in Table 1. Review of the PK concentration and drug-bound IL-18 data identified three patients in the active treatment groups with either nonquantifiable or below the limit of quantification (BLQ) data reported or much lower exposure relative to other patients within the same treatment group up to Day 29 following the first dose; these three patients were excluded from the per protocol population, as evidence suggested they did not receive an initial dose of GSK1070806.

**Table 1:** Baseline Demographics.

	Placebo (n = 12)	GSK1070806 0.25 mg/kg(n = 13)	GSK10708065 mg/kg(n = 12)
**Mean age, years (SD)**	55.1 (9.61)	58.0 (9.57)	58.7 (7.97)
**Sex, n (%)**			
**Female:**	2 (17)	4 (31)	3 (25)
**Male:**	10 (83)	9 (69)	9 (75)
**BMI (kg/m**^**2**^**), mean (SD)**	32.4 (2.65)	34.5 (3.18)	33.3 (2.43)
**Height (cm), mean (SD)**	167.2 (9.67)	164.2 (10.57)	166.0 (7.51)
**Weight (kg), mean (SD)**	90.7 (12.14)	93.1 (13.84)	92.0 (10.53)
**Ethnicity, *n* (%)**			
**Hispanic or Latino**	1 (8)	1 (8)	2 (17)
**Not Hispanic or Latino**	11 (92)	12 (92)	10 (83)
**Race, n (%)**			
**White**	12	13	12

An unexpectedly large number of patients (n = 28) screened in Spain failed screening due to a positive Quantiferon Gold test; this finding was not localized to one site but was spread across the various centers used in the study, suggesting that *Mycobacterium tuberculosis* is endemic in this population.

### Efficacy results

**Fig 2:**
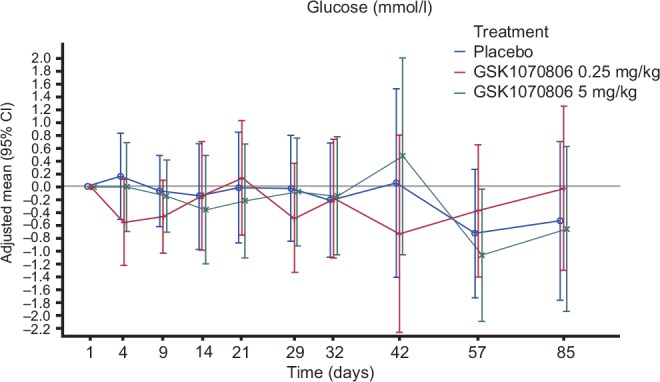
Model Adjusted Mean (95% CI) Change from Baseline Plot for Fasting Plasma Glucose (mmol/l) (All Visits up to Day 85) [Per Protocol Population].

Overall, there were no statistically significant effects of GSK1070806 doses on FPG over placebo on Days 29, 57, and 85 or other assessment days, as the 95% CIs included zero (S1 Table). During the course of the study, patients demonstrated increases or decreases in their blood glucose values, but there were no consistent trends observed across the placebo and active dose groups. Mean plasma glucose levels revealed several apparent improvements in glucose control, as determined by reductions in FPG, in a number of patients dosed with placebo and doses of GSK1070806 up to Day 85 (Fig 2). Overall, however, there were no obvious trends of improvement in active- versus placebo-treated patients.

**Fig 3:**
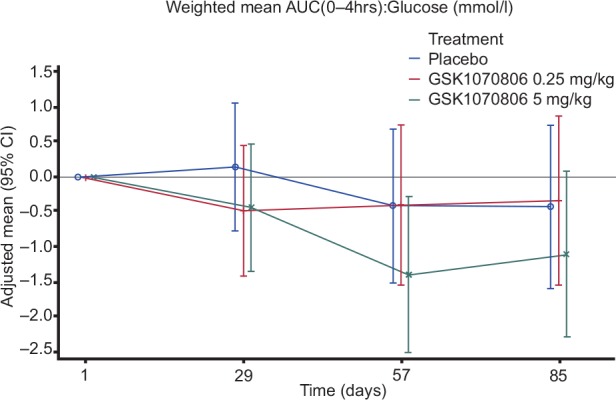
Model Adjusted Mean (95% CI) Change from Baseline Plot for Weighted Mean AUC(0–4 hours) Glucose from Mixed Meal Test (All Visits up to Day 85) [Per Protocol Population].

With the exception of insulin and C-peptide at one time point (0.25 mg/kg vs placebo on Day 29), there were also no statistically significant effects of GSK1070806 doses on glucose, insulin and C-peptide weighted mean AUC(0–4 hours) over placebo on Days 29, 57, and 85 (Fig 3, S2, S3 and S4 Tables).

**Fig 4:**
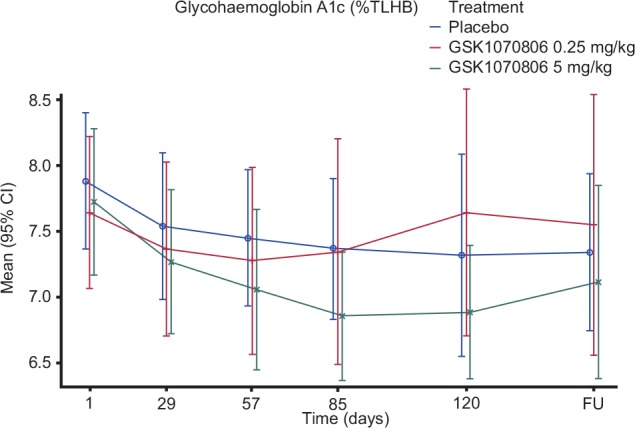
Mean (95% CI) Plot: HbA1c Percent [Per Protocol Population: Subjects 205, 212 & 203 Excluded].

Analysis of individual subject HbA1c percent profiles demonstrated that some subjects in all three treatment groups were responding to study treatment, indicated by a reduction in HbA1c percent from baseline. Assessment of the mean change from baseline (Fig 4) shows evidence of a reduction in HbA1c percent up to Day 85 with subjects dosed with GSK1070806 5 mg/kg compared with placebo, although the data are highly variable.

No underlying trends were apparent in homeostasis model assessment (HOMA)-%S- and HOMA-%B-derived parameters [31]. There were no notable differences between the treatment groups for change from baseline body mass index at Day 85 (S2 Fig). Horizontal waist circumference in the patients was variable, with some showing a small decrease and others showing an increase from baseline to Day 85 in the placebo and GSK1070806 0.25 and 5 mg/kg cohorts (S3 Fig).

### Safety and tolerability results

GSK1070806 was well tolerated, and the frequency of AEs did not increase notably as the GSK1070806 dose increased (S5 Table). No patient withdrew from the trial because of an AE. The most frequently reported AE in the trial was nasopharyngitis, which occurred in 11 patients, four patients (33%) in the placebo group, three patients (23%) in the 0.25 mg/kg GSK1070806 group, and four patients (33%) in the 5 mg/kg group. Headache, diarrhea, and hypertension were the second most frequently reported AEs that occurred in five patients each. One patient in the 5 mg/kg GSK1070806 group reported an AE of hyperesthesia that was considered by the investigator to be possibly drug related.

Two AEs, diverticulitis in one patient in the placebo group and VIIth nerve paralysis in one patient in the 5 mg/kg GSK1070806 group, were considered by the investigator to be serious. Both had resolved by the end of the study, and neither was considered to be drug related.

No apparent changes were observed for mean ECG interval values (PR, QRS, QT, QTcB, QTcF, RR, heart rate) following administration of placebo or GSK1070806 at either dose level. No apparent changes were observed in mean values for any vital signs parameter (heart rate, respiration rate, and body temperature) following administration of placebo or GSK1070806 at either dose level. Hypertension was reported by 15% of patients in the 0.25 mg/kg GSK1070806 group and 25% of patients in the 5 mg/kg GSK1070806 group (none in the placebo group). A detailed review of individual subject values suggested that despite there being individuals with variations in blood pressure, there did not appear to be an overall trend. No patients treated with GSK1070806 had detectable levels of anti-GSK1070806 binding antibodies.

### Pharmacokinetic results

Plasma PK results are summarized in S6 Table. Following the first and second dose, T_max_ was approximately 1 hour for both the 0.25 and 5 mg/kg doses. GSK1070806 AUC(0–τ), and C_max_ increased with increasing dose in an approximately dose-proportional manner (14- to 17-fold increase in parameters for a 20-fold dose increase). Limited accumulation was observed for C_max_ and AUC(0–τ), with the accumulation ratio between the first and second infusion ranging from 1.2- to 1.5-fold for these parameters. The lack of marked accumulation is consistent with the observed geometric mean half-life for GSK1070806, which ranged from 23 to 30 days. The variability on all parameters was generally low (range 17–33%, in the per protocol population).

### Pharmacodynamic and biomarker results

Target engagement for GSK1070806 was assessed in serum using an assay that specifically detected drug-bound IL-18 as previously described [30]. The lower limit of quantification (LLQ) of this assay was 19.5 pg/ml; measurements below LLQ were imputed to LLQ/2 for the purpose of plotting the data. Patients treated with 0.25 mg/kg GSK1070806 showed a mean serum drug-bound IL-18 level that increased up to approximately Day 29 and then remained relatively constant through Day 85, decreasing to the follow-up (FU) visit at Day 210 (S4A Fig). A similar pattern in mean drug-bound IL-18 levels was observed in patients treated with 5 mg/kg GSK1070806, except for a decrease in mean levels at Day 29 with levels increasing again through Day 120 followed by a decrease at the FU visit. For the placebo group, the mean drug-bound IL-18 levels remained below the LLQ of the assay and unchanged over time. The mean serum-free IL-18 levels varied across the treatment groups (S4B Fig) and, for the majority of patients, free IL-18 was BLQ at baseline.

A broad range of biomarkers associated with inflammatory, metabolic, and cardiovascular disease were assessed during the study; however, generally, biomarker levels were variable across patients, and the majority of parameters did not show any notable trends compared with the placebo group.

Assessment of the mean change from baseline for hs-CRP and IL-6 indicated some evidence of a reduction in this parameter up to Day 85, for subjects dosed with GSK1070806 5 mg/kg compared with placebo (S6 and S7 Figs). There also appeared to be a reduction in IP-10 out to Day 85 in the GSK1070806 5 mg/kg group, but the data are highly variable, and a reduction at Day 57 was also observed in the placebo group that was driven primarily by data from two individuals (S7 Fig).

## Conclusions

A body of preclinical and clinical data supports the hypothesis that chronically elevated levels of circulating inflammatory cytokines in obese and diabetic patients are associated with a range of pathological effects such as IR, pancreatic inflammation, β-cell destruction, vascular remodeling, atherosclerosis, and hypertension. Published literature suggests that levels of IL-18 in obese patients are elevated compared with normal weight individuals; therefore, patient selection in this trial was optimized to select obese patients, as this patient group was considered to be most likely to benefit from treatment with GSK1070806.

While there were no statistically significant effects of GSK1070806 doses on FPG levels, or glucose, insulin, and C-peptide weighted mean AUC(0–4 hours) over placebo, it should be noted that the trial was not formally powered to detect statistically significant differences in these measures between GSK1070806 and placebo, as the sample size was selected to allow preliminary characterization of efficacy, safety, and tolerability in obese patients with T2DM, and to investigate the effect on PD assessment and biomarkers.

One of the inclusion criteria was that patients were required to be on a stable dose of metformin for 3 months prior to screening, and at a total daily dose of ≥1,000 mg for at least 2 months prior to dosing. There was, however, a high placebo response rate in this study, with patients in both the placebo and treatment groups demonstrating decreases of >1% HbA1c by Day 57 of the study. This suggests that this patient group was generally noncompliant in taking metformin and only started to take it regularly on entry into the study. From information collected relating to concomitant medication usage, some patients had been on metformin for over 10 years at the time of entry into the study and may have stopped taking it as regularly as they should have done. In hindsight, this could have been limited by the inclusion of a run-in period so that patients entering the trial were known to be compliant with their medication. It was interesting that for some placebo-treated patients who demonstrated significant reductions in HbA1c (e.g., baseline: 8.8%; Day 57: 7.1%), notable decreases in other biomarkers (e.g., IP-10, fructosamine, NEFAs, s-ICAM) were also apparent. This suggests that metformin treatment alone also impacts many inflammatory markers, but unfortunately it also confounded the overall interpretation of any biomarker data from the study, as it was impossible to distinguish changes that were due to inhibition of GSK1070806 with those that were due to resumption or regular use of metformin monotherapy. Overall, biomarker levels were variable across patients, and the majority of parameters did not show any notable trends compared with the placebo group. Assessment of the mean change from baseline for hs-CRP and IL-6 indicates some evidence of a reduction in these inflammatory markers up to Day 85, with patients dosed with GSK1070806 5 mg/kg compared with placebo. The mean reduction in hs-CRP that was observed between Days 29 and 85 in this study ranged from 13.5% to 21.4% in the 5 mg/kg group; this is lower than has been previously reported for other effective T2DM therapies. A 12-week study that evaluated the effect of treatment with rosiglitazone or metformin on top of glimepiride on inflammatory biomarkers in patients with T2DM demonstrated a 40% reduction in serum CRP levels at Week 12; a similar magnitude of change was reported for IL-6 and resistin.

Both doses of GSK1070806 were well tolerated, with no drug-related serious AEs (SAEs) seen. The two SAEs that were reported during the course of the study were considered unrelated to study medication by the investigator: an AE of diverticulitis in one subject in the placebo group and an AE of VIIth nerve paralysis in one subject dosed with 5 mg/kg GSK1070806. Both SAEs had resolved by the end of the study.

Chronic inhibition of IL-18 has the potential to increase the risk of infections as well as affect viral and intracellular bacterial clearance by the host. However, as observed in the previous FTIH study, all AEs that described infection were mild or moderate, unremarkable, and considered unrelated to study treatment by the investigators. The lack of increase in the incidence rate of infections in GSK1070806 groups compared with placebo provides further reassurance that chronic dosing of GSK1070806 should have an acceptable safety profile.

Variations in the levels of serum IL-18 detectable at baseline were observed, as not all patients had measurable levels at baseline. Following dosing with GSK1070806 5 mg/kg, a decrease BLQ was observed in the patients with measurable serum-free IL-18 at baseline that persisted through the final time point (FU), demonstrating that prolonged inhibition of IL-18 had occurred in these patients. By contrast, serum-free IL-18 was detectable in four patients in the placebo arm at various time points. At baseline, serum drug-bound IL-18 was BLQ as expected, in all except two patients in the 0.25 mg/kg treatment group that appeared to have detectable levels of drug-bound IL-18. This was likely due to interference in the assay, as there cannot be any drug-bound IL-18 present predose.

After dosing, the mean serum drug-bound IL-18 levels increased from baseline in both groups treated with GSK1070806, demonstrating target engagement. This also confirmed that serum IL-18 was present, even in individuals that had levels BLQ when assessed using the free IL-18 assay. By contrast, in the placebo group, the serum drug-bound IL-18 levels for patients remained below the LLQ and unchanged over time.

Increased mean serum drug-bound IL-18 levels observed in the GSK1070806 treatment groups were sustained for a prolonged period of time, indicating sustained target engagement. There was no dose-dependent increase in the mean serum drug-bound IL-18 profiles, indicating that saturation of the target was achieved in the systemic compartment at both doses. Mean serum drug-bound IL-18 levels at the last measurement time point remained above the baseline value in all active dose groups. Overall, the serum drug-bound IL-18 results were consistent with what was observed in the FTIH study with GSK1070806.

Observed PK profiles in this study were overall similar to the PK profiles observed in the FTIH at equivalent doses. The observed geometric mean half-life in the 5 mg/kg dose group was lower (30 days) than in the FTIH study (37–38 days) at approximately equivalent dose and more aligned with the expectation for a monoclonal antibody. The reason for this discrepancy is unclear, but it will be monitored in future clinical studies.

In conclusion, this study demonstrated that two repeat doses of GSK1070806 administered 28 days apart were well tolerated in patients with T2DM, but no significant reductions in the primary efficacy end-points for the study were observed. There was some evidence of a reduction in HbA1c percent up to Day 85 with patients dosed with GSK1070806 5 mg/kg compared with placebo, but the data were highly variable across all treatment groups. This study demonstrated that inhibiting IL-18 does not appear to have a major impact on blood glucose control; however, given the limited power of the study, the improvements noted in the placebo arm, and the trend toward reduction in HbA1c percent with patients dosed with GSK1070806 5 mg/kg, smaller potentially clinically meaningful effects of IL-18 inhibition cannot be excluded. It is also possible that the effects of chronic inflammation via IL-18 on IR may take longer to reverse than the time period evaluated in this study.

## Supporting Information

S1 CONSORT Checklist CONSORT Checklist. (DOC)

S1 Fig Trial Schematic. FU, follow-up; MMT, mixed meal test.
(DOCX)

S2 Fig Individual Subject Plot for Body Mass Index (kg/m^2^). (DOCX)

S3 Fig Individual Subject Plot for Waist Circumference. (DOCX)

S4 Fig Mean Serum Drug-Bound and Serum-Free IL-18. Panel a shows the mean serum drug-bound IL-18 and 95% CI in the per protocol population. Panel b shows the mean serum-free IL-18 in all patients. 1:1H, Day 1 1 hour; 29:1H, Day 29 1 hour; FU, follow-up (approximately Day 210). For free IL-18 BLQ set to 1/2BLQ = 9.75 and drug-bound IL-18 BLQ set to 1/2BLQ = 2. Patients 205 and 212 (GSK1070806 0.25 mg/kg) had missing first dose, and patient 203 (GSK1070806 5 mg/kg) first dose appeared to be mis-dosed due to lower PK exposure observed compared with the group.
(DOCX)

S5 Fig Mean (95% CI) Percentage Change from Baseline Plot of hs-CRP [Per Protocol Population]. (DOCX)

S6 Fig Mean (95% CI) Percentage Change from Baseline Plot of IL-6 [Per Protocol Population]. (DOCX)

S7 Fig Mean (95% CI) Percentage Change from Baseline Plot of IP-10 [Per Protocol Population]. (DOCX)

S1 File Supplementary Methods. (DOCX)

S2 File Appendix. (DOCX)

S1 Protocol Redacted Protocol. (PDF)

S1 Table Summary of Statistical Analysis Results of Change from Baseline in Fasting Plasma Glucose (mmol/l) (All Visits up to Day 85) [Per Protocol Population]. Note: Only results for Days 29, 57, and 85 are presented.
(DOCX)

S2 Table Summary of Statistical Analysis Results of Change from Baseline in weighted Mean glucose AUC(0–4 hours) from Mixed Meal Test (All Visits up to Day 85) [Per Protocol Population]. (DOCX)

S3 Table Summary of Statistical Analysis Results of Change from Baseline in Insulin-Weighted Mean AUC(0–4 hours) from Mixed Meal Test (All Visits up to Day 85) [Per Protocol Population]. (DOCX)

S4 Table Summary of Statistical Analysis Results of Change from baseline in C-peptide Weighted Mean AUC(0–4 hours) from Mixed Meal Test (All Visits up to Day 85) [Per Protocol Population]. (DOCX)

S5 Table All Post-Treatment AEs Reported in Two or More Patients. AE, adverse event.
(DOCX)

S6 Table Plasma GSK1070806 PK Parameters in the [Per Protocol Population]. *Values represent the geometric mean (CVb%) for each parameter, except for T_max_: median (min–max). ^†^AUC(0–Week 8) = area under the curve from time 0 to Week 8 following the second dose. ^‡^Cum AUC(0–Week 12) = cumulative area under the curve from time 0 to Week 12, from the first dose. N, number of patients in cohort; n, number of patients with nonmissing values; NA, not applicable.
(DOCX)
