# Supplementary figures and images for: A Study to Investigate the Efficacy and Safety of an Anti-Interleukin-18 Monoclonal Antibody in the Treatment of Type 2 Diabetes Mellitus
Source: PLoS One. 2016 Mar 1;11(3):e0150018. doi: 10.1371/journal.pone.0150018 (PMC4773233; doi:10.1371/journal.pone.0150018)

Supplementary Figures

**S1 Fig. Trial Schematic.**

**
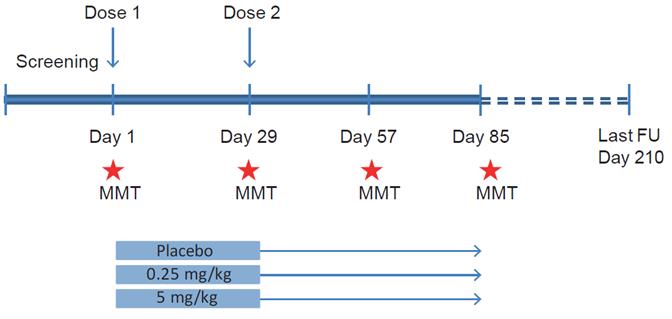
**

FU, follow-up; MMT, mixed meal test

Supplement: S1 Fig — FU, follow-up; MMT, mixed meal test. (DOCX) [file pone.0150018.s002.docx]
