# Supplementary material for: A Study to Investigate the Efficacy and Safety of an Anti-Interleukin-18 Monoclonal Antibody in the Treatment of Type 2 Diabetes Mellitus
Source: PLoS One. 2016 Mar 1;11(3):e0150018. doi: 10.1371/journal.pone.0150018 (PMC4773233; doi:10.1371/journal.pone.0150018)
Supplement: S2 Fig — (DOCX) [file pone.0150018.s003.docx]

Supplementary Figures

**S2 Fig. Individual Subject Plot for Body Mass Index (Kg/m^2^).**
