# Supplementary material for: A Study to Investigate the Efficacy and Safety of an Anti-Interleukin-18 Monoclonal Antibody in the Treatment of Type 2 Diabetes Mellitus
Source: PLoS One. 2016 Mar 1;11(3):e0150018. doi: 10.1371/journal.pone.0150018 (PMC4773233; doi:10.1371/journal.pone.0150018)
Supplement: S4 Fig — Panel a shows the mean serum drug-bound IL-18 and 95% CI in the per protocol population. Panel b shows the mean serum-free IL-18 in all patients. 1:1H, Day 1 1 hour; 29:1H, Day 29 1 hour; FU, follow-up (approximately Day 210). For free IL-18 BLQ set to 1/2BLQ = 9.75 and drug-bound IL-18 BLQ set to 1/2BLQ = 2. Patients 205 and 212 (GSK1070806 0.25 mg/kg) had missing first dose, and patient 203 (GSK1070806 5 mg/kg) first dose appeared to be mis-dosed due to lower PK exposure observed compared with the group. (DOCX) [file pone.0150018.s005.docx]

Supplementary Figures

**S4 Fig. Mean Serum Drug-Bound and Serum-Free IL-18.**

Panel A shows the mean serum drug-bound IL-18 and 95% CI in the per protocol population. Panel B shows the mean serum-free IL-18 in all patients.

**A**


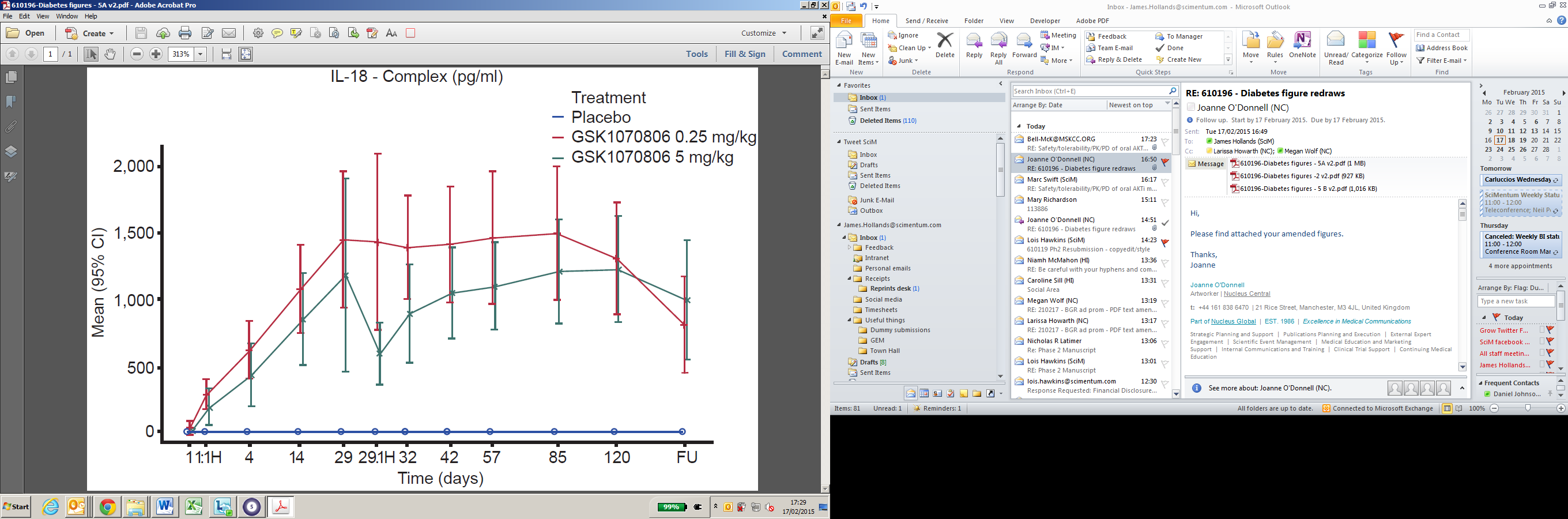


**B**


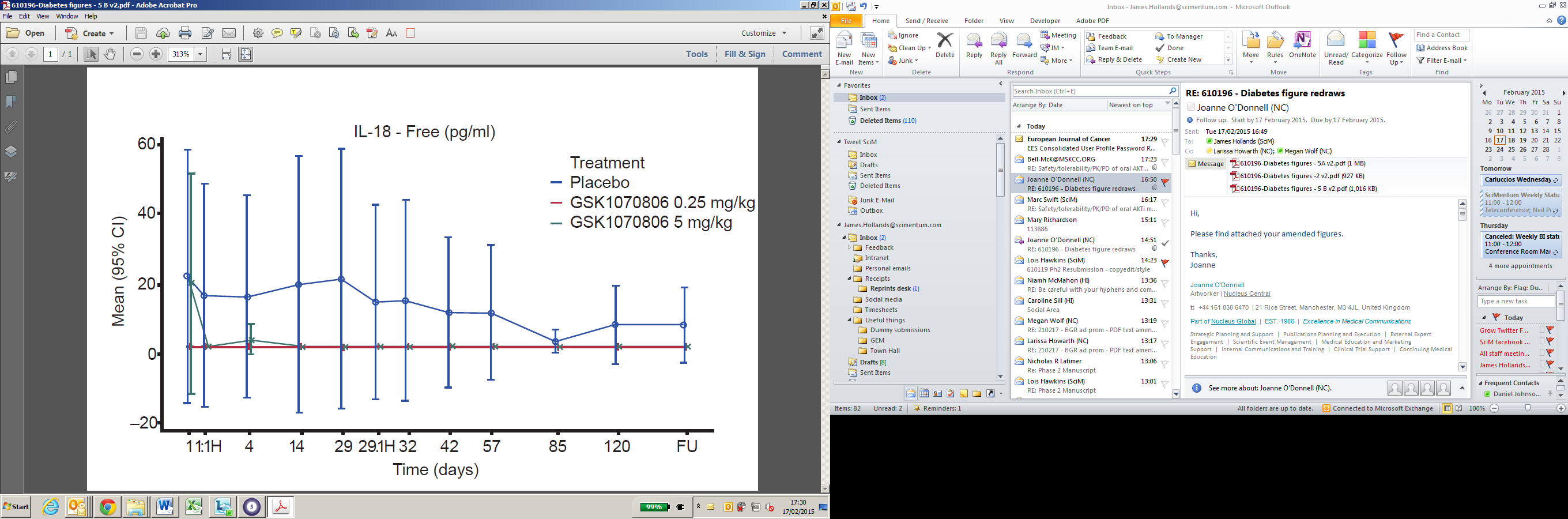


1:1H, Day 1 1 hour; 29:1H, Day 29 1 hour; FU, follow-up (approximately Day 210).

For free IL-18 BLQ set to 1/2BLQ = 9.75 and drug-bound IL-18 BLQ set to 1/2BLQ = 2.

Patients 205 and 212 (GSK1070806 0.25 mg/kg) had missing first dose and patient 203 (GSK1070806 5 mg/kg) first dose appeared to be mis-dosed due to lower PK exposure observed compared with the group.
