# Supplementary material for: A Study to Investigate the Efficacy and Safety of an Anti-Interleukin-18 Monoclonal Antibody in the Treatment of Type 2 Diabetes Mellitus
Source: PLoS One. 2016 Mar 1;11(3):e0150018. doi: 10.1371/journal.pone.0150018 (PMC4773233; doi:10.1371/journal.pone.0150018)
Supplement: S6 Fig — (DOCX) [file pone.0150018.s007.docx]

Supplementary Figures

**S6 Fig. Mean (95% CI) Percentage Change from Baseline Plot of IL-6 [Per Protocol Population].**


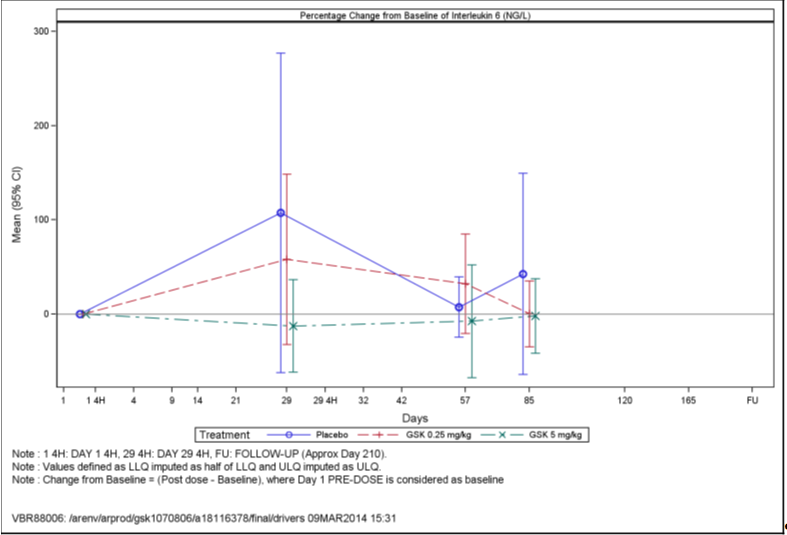


Note: Two subjects (GSK 0.25mg/kg) had missed first dose & one subject (GSK 5.0mg/kg) first dose appeared to be mis-dosed due to lower PK exposure observed compared to the group
