# Supplementary material for: A Study to Investigate the Efficacy and Safety of an Anti-Interleukin-18 Monoclonal Antibody in the Treatment of Type 2 Diabetes Mellitus
Source: PLoS One. 2016 Mar 1;11(3):e0150018. doi: 10.1371/journal.pone.0150018 (PMC4773233; doi:10.1371/journal.pone.0150018)
Supplement: S1 File — (DOCX) [file pone.0150018.s009.docx]

**Supplementary Data

S3 File: Supplementary Methods**

*Key exclusion criteria*

Other key exclusion criteria were: current evidence, or history within the last 7 days, of an influenza-like illness; use of anti-inflammatory drugs including corticosteroids, chronic maintenance therapy with non-steroidal anti-inflammatory drugs (NSAIDs), anti-TNFα or anti-IL-1 within 60 days prior to dosing; current evidence of ongoing or acute infection, or a history of repeated, chronic or opportunistic infections or a serious bacterial infection within 6 months of randomisation; history of malignancy or significant cardiac, pulmonary, metabolic, renal, hepatic, or gastrointestinal conditions; history of chronic granulomatous infections; creatinine clearance less than 60 ml/min; history of a severe allergic reaction, anaphylaxis or immunodeficiency; and current or chronic history of liver disease, or known hepatic or biliary abnormalities.

*Biomarker analyses*

Adiponectin, high sensitivity (hs)-CRP, IP-10 (CXCL10), fructosamine, hs-IL-6, sICAM, MMP-9, NEFA, PAI-1 and resistin were measured from fasted blood samples on Days 1 (pre-dose), 29, 57 and 85. Lipid levels were assessed as part of the clinical chemistry assessments. All biomarker analyses were carried out at Quest Diagnostics, using validated assays. Change from baseline in waist circumference and body mass index and incidence and titres of serum anti-GSK1070806 antibodies were also assessed.

*Statistical Analysis of Efficacy Endpoints*

Efficacy endpoints (change from baseline in FPG and change from baseline in glucose, insulin and C-peptide weighted mean AUC(0–4hrs) were analysed separately using suitable mixed models repeated measures analyses (model included fixed categorical terms [treatment, visit and treatment by visit interaction], fixed continuous covariate [baseline] and visit as repeated term), using the ‘All Patients’ (all patients who received study drug) and, where applicable, the ‘Per Protocol’ population (applicable patients excluded using the ‘All Patients’ population). Point estimates and corresponding 95% CIs were constructed for the adjusted differences between doses of GSK1070806 (0.25 and 5 mg/kg) and placebo. Safety and tolerability data was presented in tabular format and summarised descriptively according to GSK’s Integrated Data Standards Library standards. For patients in both active treatment group and for both dose occasions, GSK1070806 PK parameters were derived (where data permitted) by standard non-compartmental analysis using Phoenix WinNonlin®, (Certara Inc. Princeton, NJ, USA). Accumulation ratio for AUC(0-τ) and C_max_ following administration of two doses was estimated. Calculations were based on the actual sampling times recorded during the study. PD and biomarker data were descriptively summarised and/or graphically presented.
