# Supplementary material for: A Study to Investigate the Efficacy and Safety of an Anti-Interleukin-18 Monoclonal Antibody in the Treatment of Type 2 Diabetes Mellitus
Source: PLoS One. 2016 Mar 1;11(3):e0150018. doi: 10.1371/journal.pone.0150018 (PMC4773233; doi:10.1371/journal.pone.0150018)
Supplement: S2 File — (DOCX) [file pone.0150018.s010.docx]

**Supplementary Data**

**S4 File: Appendix**

**Participating Investigators to the Study**

Fajardo Montañana C – Hospital de la Ribera, Alzira, Spain

Gil Extremera B – Hospital Clinico San Cecilio, Granada, Spain

Garcia Puig J – Hospital la Paz, Madrid, Spain

Tinahones Madneno F – Hospital Clinico Universitario Virgen de la Victoria, Málaga, Spain

Fernández Roure JL – CAP La Roca, Barcelona, Spain

Teresa Parreño L – Instituto de Ciencias Médicas, Alicante, Spain

Santos Rey M – Hospital Universitari Aman de Vilanova, Lleida, Spain
