# Supplementary material for: A Study to Investigate the Efficacy and Safety of an Anti-Interleukin-18 Monoclonal Antibody in the Treatment of Type 2 Diabetes Mellitus
Source: PLoS One. 2016 Mar 1;11(3):e0150018. doi: 10.1371/journal.pone.0150018 (PMC4773233; doi:10.1371/journal.pone.0150018)
Supplement: S1 Protocol — (PDF) [file pone.0150018.s011.pdf]

Division: Worldwide Development
Retention Category: GRS019
Information Type: Protocol Amendment

**Title:**	A Single Blind (Sponsor-unblinded), Placebo-controlled, Parallel-group Study to Investigate the Efficacy and Safety of GSK1070806 in the Treatment of Obese Subjects with T2DM

Compound Number: GSK1070806

Effective Date: 25-JUN-2013

Protocol Amendment Number: 01

Subject: T2DM, Obese, IL-18, Monoclonal antibody (mAb), Efficacy

Author: [redacted] QSci, Biopharm; [redacted], BTM, Biopharm R&D; [redacted], CPSSO, Biopharm, CPSE; [redacted], QSci, Biopharm CPMS; [redacted] BTM, Biopharm R&D;

Revision Chronology:

2011N125769_00	2012-MAR-29	Original
2011N125769_01	2013-JUN-25	Amendment No.: 01 To update the protocol to include interim data analyses for the purpose of internal GSK decision making.

Copyright 2013 the GlaxoSmithKline group of companies. All rights reserved. Unauthorised copying or use of this information is prohibited.

SPONSOR SIGNATORY:

[redacted], MD,
VP, Biopharm Translational Medicine, R&D,
GlaxoSmithKline

Date

SPONSOR/MEDICAL MONITOR INFORMATION PAGE

Medical Monitor and Sponsor Contact Information:

Role	Name	Day Time Phone Number	After-hours Phone/Cell/ Pager Number	Fax Number	GSK Address
Primary Medical Monitor	[redacted]	[redacted]	[redacted]	[redacted]	MPC Clinical Development GlaxoSmithKline Stockley Park, Iron Bridge Road, UK,
Secondary Medical Monitor	[redacted] MD	[redacted]	[redacted]	[redacted]	BTM, Biopharmaceuticals GlaxoSmithKline Gunnels Wood Road Stevenage UK,
Tertiary Medical Monitor	[redacted] MD	[redacted]	[redacted]	[redacted]	BTM, Biopharmaceuticals GlaxoSmithKline Gunnels Wood Road Stevenage UK,

Sponsor Legal Registered Address:

GlaxoSmithKline Research & Development Limited
980 Great West Road
Brentford
Middlesex, TW8 9GS
UK

In some countries, the clinical trial sponsor may be the local GlaxoSmithKline affiliate company (or designee). If applicable, the details of the alternative Sponsor and contact person in the territory will be provided to the relevant regulatory authority as part of the clinical trial application.

Regulatory Agency Identifying Number(s): 2012-000126-22

INVESTIGATOR PROTOCOL AGREEMENT PAGE

I confirm agreement to conduct the study in compliance with the protocol, as amended by this protocol amendment.

Investigator Name:	
Investigator Address:	
Investigator Phone Number:	

Investigator Signature	Date

TABLE OF CONTENTS

PAGE

ABBREVIATIONS 9

1. INTRODUCTION 13
   1.1. Background 14
   1.2. Pre-clinical studies 14
   1.3. Interleukin 18 and microvascular co-morbitities in T2DM. 15
   1.4. Interleukin 18 and macrovascular co-morbitities in T2DM. 15
   1.5. Supporting data from trials with pharmacological intervention 16
      1.5.1. Observations from Clinical Trials with rhIL-18 (SB-485232) in Humans 17
   1.6. Previous Human Experience with GSK1070806 17
   1.7. Rationale 18
      1.7.1. Study Rationale 19
      1.7.2. Dose Rationale 20
   1.8. Summary of Risk Assessment 20
   1.9. Summary of Risk Management 21
      1.9.1. Inclusion of females of child-bearing potential 22
      1.9.2. Follow-up time for use of female contraception 22
      1.9.3. Influenza management 23
      1.9.4. Travel restrictions for subjects participating in trial 24

2. OBJECTIVE(S) AND ENDPOINTS 24

3. INVESTIGATIONAL PLAN 25
   3.1. Study Design/ Schematic 25
   3.2. Discussion of Design 26
   3.3. Treatment Assignment 27
   3.4. Investigational Product and Other Study Treatment Dosage/Administrations 27
      3.4.1. Dose Adjustment/Stopping Safety Criteria 27
         3.4.1.1. Liver Chemistry Stopping Criteria 27
         3.4.1.2. QTc Withdrawal Criteria 28
         3.4.1.3. Blood Glucose Withdrawal Criteria 28
   3.5. Time and Events Table 30

4. STUDY POPULATION 32
   4.1. Number of Subjects 32
   4.2. Eligibility Criteria 32
      4.2.1. Inclusion Criteria 32
      4.2.2. Exclusion Criteria 33
   4.3. Screen and Baseline Failures 35

5. DATA ANALYSIS AND STATISTICAL CONSIDERATIONS 35
   5.1. Hypotheses and Treatment Comparisons 35
   5.2. Sample Size Considerations 35
      5.2.1. Sample Size Assumptions 35
      5.2.2. Sample Size Sensitivity 36
      5.2.3. Sample Size Re-estimation 36
   5.3. Data Analysis Considerations 36

5.3.1. Interim Analysis 36
5.3.2. Final Analyses 37
5.3.3. Safety Analyses 37
5.3.3.1. Pharmacokinetic Analyses 38
5.3.3.2. Pharmacokinetic/Pharmacodynamic Analyses 38
5.3.3.3. Pharmacodynamic/Biomarker Analyses 39
5.3.3.4. Exploratory Biomarker(s) Analyses 40

6. STUDY ASSESSMENTS AND PROCEDURES 40
6.1. Demographic/Medical History Assessments 40
6.2. Safety 41
6.2.1. Physical Exams 41
6.2.2. Vital Signs 41
6.2.3. BP monitoring 41
6.2.4. Electrocardiogram (ECG) Monitoring 42
6.2.5. Clinical Laboratory Assessments 42
6.3. Pregnancy 43
6.3.1. Time period for collecting pregnancy information 43
6.3.2. Action to be taken if pregnancy occurs 43
6.3.3. Action to be taken if pregnancy occurs in a female partner of a male study subject 44
6.4. Pharmacokinetics 44
6.4.1. Blood Sample Collection 44
6.4.2. Sample Analysis 44
6.5. Anti-GSK1070806 Antibodies 44
6.5.1. Blood Sample Collection 44
6.5.2. Sample Analysis 45
6.6. Biomarker(s) 45
6.6.1. Inflammatory and Metabolic Biomarkers 45
6.6.2. Exploratory Biomarker Analysis 45
6.6.3. Biomarkers in urine 45
6.7. Free and drug-bound IL-18 levels in serum 45
6.8. Clinical function assessments 46
6.8.1. Mixed Meal test (MMT) 46
6.8.2. BMI and Waist Circumference 46
6.9. Pharmacogenetics 46

7. LIFESTYLE AND/OR DIETARY RESTRICTIONS 46
7.1. Contraception Requirements 46
7.1.1. Female Subjects 46
7.1.2. Male Subjects 47
7.2. Meals and Dietary Restrictions 47
7.3. Caffeine, Alcohol, and Tobacco 48
7.4. Activity 48
7.5. Travel 48

8. CONCOMITANT MEDICATIONS AND NON-DRUG THERAPIES 49
8.1. Permitted Medications 49
8.2. Prohibited Medications 49
8.3. Non-Drug Therapies 49

9. COMPLETION OR EARLY WITHDRAWAL OF SUBJECTS 50

9.1. Subject Completion....50
9.2. Subject Withdrawal Criteria....50
9.3. Subject Withdrawal Procedures....50
9.3.1. Subject Withdrawal from Study....50
9.3.2. Subject Withdrawal from Study Treatment....51
9.4. Treatment After the End of the Study....51

10. STUDY TREATMENT....51
10.1. Blinding....51
10.2. Packaging and Labeling....51
10.3. Preparation/Handling/Storage/Accountability....51
10.4. Assessment of Compliance....52
10.5. Treatment of Investigational Product Overdose....52

11. ADVERSE EVENTS (AE) AND SERIOUS ADVERSE EVENTS (SAE)....53
11.1. Definition of Adverse Events....53
11.2. Adverse Events of Special Interest....54
11.2.1. Home Blood Glucose Monitoring....54
11.2.2. Hypoglycemia Events....54
11.2.3. Allergic Reactions....55
11.2.4. Infections....55
11.2.5. Influenza Management....55
11.3. Definition of Serious Adverse Events....56
11.4. Method of Detecting AEs and SAEs....57
11.5. Recording of AEs and SAEs....57
11.6. Evaluating AEs and SAEs....57
11.6.1. Assessment of Intensity....57
11.6.2. Assessment of Causality....58
11.7. Follow-up of AEs and SAEs....58
11.8. Prompt Reporting of SAEs to GSK....59
11.9. Regulatory Reporting Requirements For SAEs....59

12. LIVER CHEMISTRY FOLLOW-UP PROCEDURES....60
12.1. Restarting Investigational Product....62

13. STUDY CONDUCT CONSIDERATIONS....62
13.1. Posting of Information on Publicly Available Clinical Trial Registers....62
13.2. Regulatory and Ethical Considerations, Including the Informed Consent Process....62
13.2.1. Urgent Safety Measures....63
13.3. Quality Control (Study Monitoring)....63
13.4. Quality Assurance....64
13.5. Study and Site Closure....64
13.6. Records Retention....64
13.7. Provision of Study Results to Investigators, Posting of Information on Publically Available Clinical Trials Registers and Publication....65
13.8. Data Management....66

14. REFERENCES....67

APPENDICES....74
Appendix 1: Liver Safety Algorithms....74

Appendix 2: Liver Safety Drug Restart Guidelines 75
Appendix 3: Pharmacogenetic research 78
Appendix 4: Hypoglycemia monitoring 82
Appendix 5: Protocol Amendment Changes 84

ABBREVIATIONS

ACR	Albumin Creatinine Ratio
AE	Adverse Event
ALT	Alanine aminotransferase (SGPT)
ANOVA	Analysis of Variance
AOSD	Adult-Onset Still's Disease
AST	Aspartate aminotransferase (SGOT)
AUC	Area under concentration-time curve
AUC(0-∞)	Area under the concentration-time curve from time zero (pre-dose) extrapolated to infinite time
AUC(0-τ)	Area under the concentration-time curve over the dosing interval
BMI	Body mass index
BP	Blood pressure
BTM	Biopharm Translational Medicine
BUN	Blood urea nitrogen
CIB	Clinical Investigator's Brochure
CL	Systemic clearance of parent drug
Cmax	Maximum observed concentration
CPK	Creatine phosphokinase
CPMS	Clinical Pharmacokinetics Modelling & Simulation
CPSE	Centre for Clinical Study Excellence
CPSS0	Clinical Pharmacology Science and Study Operations
CRF	Case Report Form
CRP	C-reactive Protein
CUC	Clinical Unit Cambridge
CV	Cardiovascular
DBP	Diastolic blood pressure
DDS	Drug Development Sciences
DILI	Drug Induced Liver Injury
DMPK	Drug Metabolism and Pharmacokinetics
DNA	Deoxyribonucleic acid
DRE	Disease Related Event
ECG	Electrocardiogram
ECL	Immunoelectrochemi-Luminescent
EDC	Electronic data capture
EISR	Expedited Investigator Safety Report
FasL	Fas and Fas ligand
Fabs	Absolute bioavailability of drug determined following extravascular and intravascular dosing
FDA	Food and Drug Administration
FSH	Follicle Stimulating Hormone
FTIH	First time in humans
GCP	Good Clinical Practice
GCSP	Global Clinical Safety and Pharmacovigilence
GGT	Gamma glutamyltransferase

GLP	Good Laboratory Practice
GLS	Geometric Least-Squares
GSK	GlaxoSmithKline
HBsAg	Hepatitis B surface antigen
hCG	Human chorionic gonadotropin
HDL	High-Density Lipoprotein
HGE	Capture Hypoglycemic Events
HIV	Human Immunodeficiency Virus
HOMA	homeostasis model assessment
HPLC	High-Performance Liquid Chromatograph
h/hr	Hour(s)
HR	Heart rate
HRT	Hormone Replacement Therapy
HWE	Hardy-Weinberg Equilibrium
IB	Investigator's Brochure
IBD	Inflammatory Bowel Disease
ICH	International Conference on Harmonization of Technical Requirements for Registration of Pharmaceuticals for Human Use
IDMC	Independent Data Monitoring Committee
IDSL	Integrated Data Standards Library
IEC	Independent Ethics Committee
IFN-γ	Interferon Gamma
IgG1	Immunoglobulin G 1
IL-6	Interleukin-6
IL-18	Interleukin-18
IND	Investigational New Drug
IP	Investigational Product
IR	Insulin Resistance
IRB	Institutional Review Board
IRS1	Insulin Receptor Substrate 1
IUD	Intrauterine Device
IUS	Intrauterine System
IV	Intravenous
JNK	c-Jun N-terminal kinase
Kg	Kilogram
L	Liter
LADA	Latent Autoimmune Diabetes of Adults
LDH	Lactate Dehydrogenase
LDL	Low-Density Lipoprotein
LSLV	Last Subject's Last Visit
λz	Terminal phase rate constant
μg	Microgram
μL	Microliter
mAbs	Monoclonal Antibodies
MCH	Mean corpuscular hemoglobin
MCHC	Mean corpuscular hemoglobin concentration

MCP	Monocyte Chemoattractant Protein
MCV	Mean corpuscular volume
MedDRA	Medical Dictionary for Regulatory Activities
Mg	Milligrams
mL	Milliliter
MMPs	Matrix Metalloproteases
MMT	Mixed Meal Test
MSDS	Material Safety Data Sheet
msec	Milliseconds
NK	Natural Killer
NOD	Non-Obese Mouse
OGTT	Oral Glucose Tolerance Test
PAI	Plasminogen Activator Inhibitor
PCOS	Polycystic Ovary Syndrome
PD	Pharmacodynamic
PGx	Pharmacogenetics
PK	Pharmacokinetic
QT	ECG QT interval
QTcB	QT duration corrected for heart rate by Bazett’s formula
QTcF	QT duration corrected for heart rate by Fridericia’s formula
RA	Rheumatoid Arthritis
RAP	Reporting and Analysis Plan
RBC	Red blood cells
R & D	Research and Development
RNA	Ribonucleic acid
SAE	Serious adverse event(s)
sICAM	Soluble Intercellular Adhesion Molecule
SLE	Systemic Lupus Erythematosus
SOP	Standard Operating Procedure
SPM	Study Procedures Manual
sVCAM	Soluble Vascular Adhesion Molecule
t½	Terminal phase half-life
T2DM	Type 2 Diabetes Mellitus
TB	Tuberculosis
T_{CM}	Central Memory T Cells
T_h1	T Helper 1 Cells
tmax	Time of occurrence of Cmax
TNF-a	Tumor Necrosis Factor a
TNFR	TNF Receptors
ULN	Upper limit of normal
Vss	Volume of Distribution at Steady State
VP	Vice president
WCBP	Women of Childbearing Potential
WBC	White blood cells
WHO	World Health Organisation
WHR	Waist to Hip Ratio

Trademark Information

Trademarks of the GlaxoSmithKline group of companies
RELENZA

Trademarks not owned by the GlaxoSmithKline group of companies
Chiron RIBA
WinNonlin

1. INTRODUCTION

IL-18 is a member of the IL-1 family of cytokines and which was originally described as an interferon gamma (IFN-γ) inducing factor [Okamura, 1995]. The cytokine is produced constitutively in many different cell types, including macrophages, endothelial cells, vascular smooth muscle cells, dendritic cells and Kupffer cells. IL-18 is also produced in adipocytes [Shah, 2003; Skurk, 2005], but non-adipocyte cells have been identified as the main source of IL-18 in adipose tissue [Fain, 2006]. Over the past decade, it has become clear that IL-18 has more widespread functions than induction of IFN-γ These functions include regulation of gene expression including those for angiogenic factors like vascular endothelial growth factors [Amin, 2007], modulation of proliferation of various cell types including immune and nonimmune cells [Tomura, 1998; Tominaga, 2000; Khan, 2008], and regulation of cell migration and metastasis [Jung, 2006; Park, 2007].

One of the key biological functions of IL-18 is its role in host defense against microbial pathogens. IL-18 primes both innate and acquired immunity to viruses and other intracellular pathogens through activation and differentiation of T helper 1 cells (T_h1) and Natural Killer (NK) cells, the production of the pro-inflammatory cytokine IFN-γ, upregulation of Fas and Fas ligand (FasL), and also potentiation of other proinflammatory mediators. Studies in mice in response to challenge with the intracellular pathogen *Mycobacterium avium* have shown a strong requirement for a T_h1 response and a crucial role for IL-18 in the expulsion of the pathogen [Takeda, 1998].

In addition to its role in the inflammatory response to microbes, recent studies have elucidated a broad spectrum of effector functions that implicate IL-18 as an important factor in human autoimmune diseases and metabolic diseases. Numerous studies in animals and humans implicate elevated levels of IL-18 as a contributing factor to pathology of various diseases including diabetes mellitus, adult-onset Still's disease (AOSD), systemic lupus erythematosus (SLE), rheumatoid arthritis (RA), inflammatory bowel disease (IBD), vasculitis, allograft rejection, atherosclerosis, atopy, and syndromes associated with acute tissue injury such as acute pancreatitis and fulminant hepatic failure.

GSK1070806 is a humanised Immunoglobulin G 1 (IgG1) antibody that binds to human IL-18 with high affinity (KD = 30.3pM), and neutralises its function. GSK1070806 cross reacts with rhesus/cynomolgus IL-18 with high affinity (KD = 108 pM), but not mouse, rat, dog or pig IL-18. Biacore analysis and Elisa binding assays have shown that binding of GSK1070806 to IL-18 does not interfere with the binding of the natural endogenous inhibitor (IL-18BP) to IL-18. An initial Phase I study in healthy volunteers and healthy obese subjects has completed dosing and long term follow-up is on-going; GSK1070806 was well-tolerated in 57 subjects at doses up to 10mg/kg. Three cohorts of obese subjects (dosed at 0.25, 1 or 3mg/kg) were included in the study to enable evaluation of the effect of GSK1070806 on metabolic parameters. Data generated from these obese cohorts tends to indicate that subjects with fasting glucose levels close to or above the upper limit of normal (ULN) at baseline show an improvement in glucose control following oral glucose tolerance test (OGTT) challenge. It was considered that further investigation of this effect in subjects with T2DM was warranted.

1.1. Background

T2DM is a heterogeneous disorder characterized by multiple defects in insulin action in tissues (muscle, adipose and liver) and defects in pancreatic insulin secretion which eventually leads to loss of pancreatic insulin-secreting cells. The treatment goals for T2DM subjects are effective control of blood glucose, blood pressure, and lipids (if elevated) and, ultimately, to avert the serious complications associated with sustained tissue exposure to excessively high glucose concentrations. The associated complications of diabetes, such as cardiovascular disease, peripheral vascular disease, stroke, diabetic neuropathy, amputations, renal failure, and blindness result in increasing disability, reduced life expectancy, and enormous health costs.

A growing body of evidence suggests that chronic subclinical inflammation plays an important role in the pathogenesis of T2DM. Elevated concentrations of various inflammatory markers in the circulation have been reported in humans with insulin-resistance, e.g., interleukin-6 (IL-6) [Fernandez, 2000]; tumor necrosis factor a (TNF-α) [Mishima, 2002]; soluble TNF receptors (sTNFR1, sTNFR2) [Lin, 2004] and C-reactive protein (CRP). In patients with T2DM adipose tissue contributes significantly to the levels of pro-inflammatory cytokines in the circulation.

Supporting evidence from studies in humans demonstrates elevated IL-18 levels in serum, adipose tissue and muscle in obese subjects with insulin resistance (IR) and subjects with T2DM or metabolic syndrome [Fischer, 2005; Bosch, 2005a; Bosch, 2005b; Thorand, 2005; Escobar-Morreale, 2004; Hung, 2005; Troseid, 2010]. The correlation between IL-18 levels and IR in T2DM subjects appears to be independent of TNF-α, CRP, and IL-6 levels. In contrast to TNF-α and IL-6, IL-18 levels also correlate with early signs of IR in non-diabetic "healthy" individuals [Fischer, 2005].

1.2. Pre-clinical studies

The majority of investigations into IL-18 antagonists in the field of diabetes have focussed on the type 1 (autoimmune) diabetes; IL-18BP has been shown to attenuate the progression of diabetes in the non-obese mouse (NOD) model when using a prophylactic dosing regimen to young mice. The authors postulate a possible protective effect of IL-18BP on β-cell apoptosis [Zaccone, 2005] which is supported by e*x vivo* data demonstrating that IL-18BP protects β-cells from apoptosis in β-cell destruction assays [Lewis, 2006]. Assuming that IL-18 plays a similar role in T2DM inhibition of the cytokine could potentially have a significant benefit on disease given that the prevention of β-cell destruction is a key need that current therapies do not fulfil.

A recent study by Marleaut (2011) has demonstrated a novel role for anti-IL18 in T1DM in the NOD mouse model. The authors demonstrated that IL-18 promotes naïve T cell activation and vigorous T cell expansion resulting from self antigen encounter. In parallel, IL-18 promotes the terminal differentiation of effector T cells, inducing a switch from IL-17-producers into IFN-γ expressing cells. These functions render IL-18 uniquely necessary for diabetes pathogenesis in the NOD mouse, unlike IL-12 and IFN-γ, which are redundant in genetically deficient mice. IL-18 upregulation has long been associated with intra-islet inflammation [Lewis, 2006]; however, the observation that IL-18 is

fundamental for the expansion of the earliest autoreactive T cells during prediabetes provides a novel perspective of its role in T1DM and points to potential utility for GSK1070806 in the treatment of patients with Latent Autoimmune Diabetes of Adults (LADA).

1.3. Interleukin 18 and microvascular co-morbitities in T2DM.

Circulating levels of IL-18 appear to be causally implicated in a number of co-morbidities of obesity and T2DM. Several lines of evidence implicate IL-18 in the direct induction of renal injury in diabetic nephropathy, in addition to its inflammatory effect and role in oxidative stress. In patients with diabetic nephropathy, expression of IL-18 is increased in renal biopsies in proximal and epithelial tubular cells [Miyauchi, 2009]. Patients with T2DM have not only significantly higher serum, but also higher urinary levels of IL-18 compared to healthy controls [Moriwaki, 2003; Wong, 2007, Nakamura, 2005]. Moreover, there is a positive correlation between IL-18 levels in diabetic patients and the development of urinary albumin excretion, with the highest IL-18 levels found in patients with microalbuminuria and clinical albuminuria [Moriwaki, 2003; Wong, 2007; Araki, 2007]. Increased expression of IL-18 is also implicated in the pathogenesis of diabetic neuropathy and diabetic retinopathy, although data suggests that it is most closely linked to the development of diabetic nephropathy [Fujita, 2010]. Although good glycaemic control remains the accepted means of inhibiting the complications associated with T2DM, inhibition of inflammation is anticipated to reduce the incidence of microvascular disease.

1.4. Interleukin 18 and macrovascular co-morbitities in T2DM.

In the cardiovascular system, increased levels of IL-18 have been identified in atherosclerotic plaques and a role for this cytokine has been suggested in plaque progression [Mallat, 2001; Gerdes, 2002]. Elevated levels of IL-18 are also seen following myocardial infarction, heart failure and stroke [Welsh, 2010; Hulthe, 2006; Yuen, 2007]. That this cytokine plays a fundamental role in ischemia-induced myocardial remodeling was illustrated in studies utilizing antibodies to neutralize IL-18 [Venkatachalam, 2009]. In a mouse model of ischemia/reperfusion, administration of IL-18 antibodies resulted in reduced tissue damage and infarct size. IL-18 plays an important role in myocardial hypertrophy as indicated by treatment of isolated cardiomyocytes [Chandrasekar, 2005] and mice with this cytokine [Woldbaek, 2005]. Treatment of isolated heart fibroblasts with IL-18 results in the rapid activation of the c-Jun N-terminal kinase (JNK) and phosphoinositide 3-kinase (PI3-kinase) pathways [Fix, 2011]. Studies with pharmacological inhibitors illustrated that activation of these pathways is critical to interleukin-18 mediated alterations in fibroblast function. These studies illustrate the diverse roles that IL-18 plays in cardiac tissue maintenance remodelling and repair. Plasma levels of IL-18 also show a positive correlation with intimal-medial thickening and predict future cardiovascular events in T2DM patients [Yamagami, 2005].

Clinical and population studies have also consistently found increased circulating levels of IL-18 in patients with hypertension. Although obesity, and possibly age, is a determinant of plasma IL-18 levels, the relationship of IL-18 to hypertension seems to be independent of these factors [Rabkin, 2009]. It is thought that IL-18 may contribute to the pathophysiology of atherosclerosis by increasing the expression of proinflammatory cytokines, adhesion molecules, and matrix metalloproteases (MMPs) which are associated with plaque formation. In addition, either directly or through oxidative stress pathways and MMPs, IL-18 may alter endothelial function or induce vascular smooth muscle cell migration and/or proliferation to produce the vascular changes that occur with hypertension.

A recent large study concluded that the use of long-term intensive therapy to achieve target HbA1c in T2DM patients, compared with standard therapy, increased mortality and did not significantly reduce major cardiovascular (CV) events; [Gerstein, 2008] and studies which tighten glycaemic control have actually been shown to increase hypoglycaemia, [The ADVANCE Collaborative Group, 2008, Duckworth, 2009] especially in patients approaching target HbA1c. Thus, tight glycemic control burdens patients with complex treatment regimens, risk of hypoglycemia, possible weight gain, and relatively high costs while offering uncertain benefits in return [Montori, 2009]. Therefore novel treatments which control hyperglycemia and its adverse consequences without increasing CV or other risks are required.

1.5. Supporting data from trials with pharmacological intervention

In obese women undertaking nutritional and lifestyle interventions, significant reductions in weight, body fat, WHR, and IR were associated with reductions in serum IL-18 levels [Esposito, 2002; Bruun, 2007]. In T2DM patients, a number of studies have demonstrated the effect of salsalate on correcting the inflammatory aspects of T2DM with a commensurate improvement in IR [Goldfine, 2008]. In a comparator study of metformin and rosiglitazone combination vs. metformin monotherapy, a range of inflammatory serum biomarkers were measured and evaluated against clinical metabolic parameters. A significant reduction in peripheral TNF-α, IL-6, and IL-18 levels was observed in the rosiglitazone+metformin group but not in the metformin monotherapy group. In this study, changes in IL-18 concentration (but not other inflammatory biomarkers) were negatively correlated with changes in the homeostasis model assessment (HOMA)-β and a change in the IL-18 level was found to be an independent risk factor for the HOMA-β change. These results indicated that decreases in IL-18 levels may be associated with improvements in β-cell function inpatients with T2DM [Kim, 2007].

A number of clinical trials have recently been initiated or completed with various IL-1β antagonists in T2DM patients. The first study to investigate this mechanism in patients with T2DM involved administration of a 13-week course of the endogenous IL-1 receptor antagonist (IL-1Ra) Anakinra (Kineret) and demonstrated modestly improved glycaemic control (0.4% reduction in HbA1c) and evidence of improved pancreatic function (10% increase in C-peptide$_{AUC}$ after IV glucose stimulation test vs. 10% decrease in placebo arm) [Larsen, 2007]. More recently single doses of a potent anti-IL-1β monoclonal antibody Gevokizumab ("XOMA052", Xoma) demonstrated a more pronounced

improvement of long-term glycaemic control (0.6% reduction in HbA1c at day 28 after single 0.3mg/kg dose and pancreatic function in patients with T2DM [Donath, 2008]. Decreases in systemic markers of inflammation, including CRP and ESR were also reported.

A subsequent Phase IIB study with Gevokizumab did not deliver the anticipated improvements in glucose control or reductions in HbA1c in patients on metformin monotherapy. No significant differences between active and control groups were observed following 6-monthly treatment. Highly significantly decreases in CRP and increases in HDL were however observed in some dose groups, supporting further development of the molecule for the treatment of cardiovascular disease. Other anti-IL-1β monoclonal antibodies (e.g. Canakinumab, Novartis AG) are also being investigated for treatment of T2DM in ongoing Phase II trials.

1.5.1. Observations from Clinical Trials with rhIL-18 (SB-485232) in Humans

GSK is currently investigating the utility of recombinant human IL-18 (rhIL-18; SB-485232) for the treatment of a range of cancers. In a number of Phase I studies patients with concomitant medical conditions such as diabetes were eligible for participation provided their disease was considered stable by the principal investigator and that they had been receiving treatment for at least 6 months. Across two Phase I and one Phase II dose-finding studies conducted to date, the most common clinical chemistry abnormality encountered during dosing of rhIL-18 was hyperglycemia. Across the three completed and reported dose finding studies (n=72), 8 patients experienced a Grade 3 (>13.9 - 27.8 mmol/L) hyperglycaemic event and 1 patient experienced Grade 4 (>27.8 mmol/L) hyperglycemia. All nine of these patients were diagnosed as diabetic at the time of randomisation.

No clear dose-response relationship with plasma blood glucose levels was evident across the studies and, since there was no placebo group, it is impossible to assign a clear cause-and-effect relationship between rhIL-18 levels and the observed hyperglycaemic events. However, the consistent timing of hyperglacaemia AEs relative to dosing (5-10 days post infusion), in addition to the magnitude of increase in blood glucose, suggest these events were related to dosing with SB485232.

1.6. Previous Human Experience with GSK1070806

A first Time in Human (FTIH) study with GSK1070806 [Protocol A18110040; GlaxoSmithKline Document Number GM2007/00045/09] is on-going. All data are available for Cohorts I-VIIIand data up to day 56 are available for Cohort IX.

Study A18110040 was designed to evaluate the safety, tolerability, pharmacokinetics, and pharmacodynamics of a wide range of doses of GSK1070806 in healthy (6 cohorts) and obese subjects (3 cohorts). A total of 57 subjects were dosed with active drug at doses ranging from 0.008 mg/kg to 10.0 mg/kg. Safety data from this study indicated that GSK1070806 is generally well-tolerated at all doses investigated with an acceptable safety profile to support continued clinical development. The overall rate of AEs was low

and similar to placebo. To date most commonly reported AEs were nasopharyngitis, oropharyngeal pain, and headache. No clinically significant effects on laboratory endpoints, vital signs, telemetry and Holter recording or ECG recordings were observed in Part 1 of the study. In Part 2 of the study a single clinically significant Holter recording was noted and one subject experienced an episode of food poisoning which resulted in elevated liver enzymes. Neither of these were considered to be related to administration of GSK1070806. Four serious adverse event (SAEs) were reported during this study, none of which were considered to be due to administration of GSK1070806.

The observed preliminary pharmacokinetics profiles after single dose IV administration in healthy volunteers and obese subjects were consistent overall with the pharmacokinetics profile of other mAbs targeting a soluble ligand. The pharmacokinetic (PK) profile of GSK1070806 supports once monthly dosing in subjects with T2DM.

Ex- vivo pharmacodynamic effects of GSK1070806 have been assessed in all cohorts in the study using a whole blood stimulation assay (WBA). There was an indication of a time and dose dependent inhibition of stimulated IFN-γ production and down-regulation of NK cell surface activation/differentiation marker expression (CD69 and CD274) in blood samples from dosed subjects. The inhibition kinetics of these responses was rapid, with significant changes by 1 hour post dose and maximal effects observed by 48 hours post dose. The duration of effect was prolonged (>150 days at the 10mg/kg dose).

Analysis of data generated during OGTT challenge in obese subjects dosed with 0.25mg/kg to 3.0mg/kg GSK1070806 tends to indicate that subjects with fasting glucose levels close to or above the ULN at baseline show an improvement in glucose control following administration of GSK1070806. It was considered that further investigation of this effect in subjects with T2DM was warranted.

1.7. Rationale

Prevention and control of diabetes with diet, weight control, and physical activity has been difficult to achieve. Treatment of T2DM has centered on (1) increasing insulin levels, either by direct insulin administration or oral agents that promote insulin secretion (insulin secretagogues, such as oral sulfonylureas), (2) improving tissue sensitivity, such as with insulin sensitizer biguanide metformin or thiazolidinediones (TZDs), or (3) reducing the rate of carbohydrate absorption from the gastrointestinal tract by the use of a-glucosidase inhibitors (such as acarbose) or agents that decrease gastric motility. Progressive β-cell dysfunction and β-cell failure are fundamental pathogenic features of T2DM, and, ultimately, the development and continued progression of diabetes is a consequence of the failure of the β-cell to overcome IR. Current therapies become less effective over time as a result of progressive loss of β-cell function and number, with the result that a majority of T2DM subjects do not achieve current glycemic goals as reflected by HbA1c greater than 7% in more than 60% of treated subjects. Intensive efforts have been made to develop newer classes of drugs to control hyperglycemia in T2DM subjects and preserve β-cell number/ function.

Although IR is characterized by complex interactions between genetic determinants, nutritional factors, and lifestyle, it is increasingly accepted that mediators synthesized from cells of the immune system as well as by adipose tissue are critically involved in the regulation of insulin action [Tilg, 2008]. The observation that obese/diabetic individuals exhibit elevated levels of proinflammatory cytokines lends support to the hypothesis that obesity-induced IR is an inflammatory condition [Grimble, 2002; Pickup, 1998] and that inflammation, IR, and aberrant lipid metabolism may be interlinked components of the metabolic syndrome [Pickup, 1997]. IL-1β and IL-18 are closely related structurally and share numerous characteristics in their activation by capsases and signalling through IL-1R family members. Both IL-1β and IL-18 have been shown to adversely impact β-cell function and survival (via FasL-mediated apoptosis), are elevated in subjects with metabolic diseases, may directly or indirectly act on the hypothalamus to regulate appetence, and may also modulate glucose metabolism through regulation of insulin receptor efficacy. The link between the action of cytokines like IL-18 and IR has a mechanistic basis involving the activation of JNK1/STAT kinase pathway and phosphorylation of the serine/threonine insulin receptor substrate 1(IRS1) [Solinas, 2007] which is required for efficient signalling of insulin through its cognate receptor. These observations have triggered renewed interest in the potential use of anti-inflammatory strategies as potentially disease modifying treatments for diabetes and metabolic syndrome.

1.7.1. Study Rationale

This study will be the first investigation of GSK1070806 in obese subjects with T2DM and is primarily designed to investigate the efficacy, safety and tolerability of two repeat intravenous doses of this monoclonal antibody. This study will also investigate antibody distribution (pharmacokinetics) and its subsequent impact on inflammatory and metabolic parameters that may be deregulated by abnormal levels of IL-18 (pharmacodynamics).

A further key aim of this study is to better understand the mechanisms by which GSK1070806 exerts its therapeutic benefit in T2DM. As discussed in Section 1.3 above, there is an expectation that neutralisation of elevated levels of circulating IL18 will prevent the gradual loss of β-cell function which is observed in subjects with T2DM, thus providing disease modifying therapy. It may also act directly or indirectly on the hypothalamus to regulate appetence, and it may also modulate glucose metabolism through regulation of insulin receptor efficacy. The study will be designed to enable an assessment of the effects of GSK1070806 on β-cell function, insulin resistance and appetence.

In addition to its direct effect on the pathology of T2DM, it is postulated that GSK1070806 may have a beneficial effect on the micro- and macro-vascular complications of T2DM. The inclusion of subjects with microalbuminuria (defined as the daily excretion of 30 to 300mg albumin in urine <u>or</u> ACR ≥3.5 mg/mmol (female) or ≥2.5 mg/mmol (male) but ≤30mg/mmol in the study will enable an investigation into the effects of GSK1070806 on markers and endpoints relevant to diabetic nephropathy. Markers of cardiovascular disease will also be explored.

The study will be conducted in obese diabetic subjects on the basis that levels of IL-18 in obese subjects are elevated compared to normal weight individuals, and it is considered that this is the population which is most likely to benefit from an anti-IL18 therapy. A wealth of preclinical and clinical data supports the hypothesis that chronically elevated peripheral IL-18 in obese and diabetic subjects mediates a range of pathological effects such as IR, pancreatic inflammation, β-cell destruction, vascular remodelling, atherosclerosis, and hypertension.

1.7.2. Dose Rationale

A wide range of doses of GSK1070806 were investigated in an on-going FTIH study; from 0.008 mg/kg up to 10 mg/kg in healthy volunteers and from 0.25 mg/kg to 3 mg/kg in obese subjects. These doses have been well tolerated with an acceptable safety profile.

Repeat dosing (2 doses 28 day apart) of GSK1070806 given intravenously at two dose levels (a low and a high dose) is proposed in this study with the aim of beginning to understand the dose response relationship. In the FTIH study (A18110040), after a single intravenous administration of GSK1070806 at a dose level of 0.25 mg/kg in obese subjects, a trend for a decrease in blood glucose levels after an oral glucose tolerance test was observed in all 3 subjects on active drug on day 28 and in 2 out of 3 subjects on active at day 56. This study aims to confirm the trend observed in obese subjects in subjects with T2DM and therefore a low dose of 0.25 mg/kg is proposed in this study.

Targeting IL- 18 in T2DM is an unprecedented mechanism therefore to fully explore the effect of GSK1070806 over a broad dose range a high dose of 5 mg/kg has been selected. In study A18110040, based on the interim analysis of the WBA data, maximum ex-vivo PD effect was observed up to day 56 in particular on IFNγ (i.e. inhibition) with no clear differentiation between 1 and 3 mg/kg in obese subjects. The high dose of 5 mg/kg is therefore expected to maximize pharmacological effects, based on the ex-vivo pharmacodynamics data generated in the WBA in healthy and obese subjects, while also taking into account the change in the population studied (i.e. T2DM) and the likelihood that these subjects will have higher levels of inflammatory cytokines. This higher dose of 5 mg/kg provides also a clear differentiation with the low dose proposed to be evaluated in the study.

Furthermore, other monoclonal antibodies (e.g. XOMA052 and Canakinumab) targeting another pro-inflammatory cytokine from the same family IL1β and also developed for T2DM have reported a reduction in %HbA1c and CRP levels and increase in HDL at low doses. This data supports the adequate distribution of a monoclonal antibody into the relevant site of action for the T2DM indication.

1.8. Summary of Risk Assessment

IL-18 plays an important role in establishing host immune responses against infectious pathogens via the induction of IFN-γ. Chronic inhibition of IL-18 function has the potential to increase the risk of infections as well as impacting on viral and intracellular bacterial clearance by the host. However, due to the redundancy in the pathways leading to the production of IFN-γ, the risk for infections may not be significantly increased in the clinic and may not be different from currently marketed anti-inflammatory medications e.g. Humira, Ustekinumab.

Given the role of IL18 in innate immunity, a series of preclinical studies were conducted to investigate the effects of GSK1070806 on the immune system. These studies demonstrated that treatment with GSK1070806 did not affect the cytolytic activity of natural killer (NK) cells, chemotaxis or oxidative burst capacity of neutrophils, nor did it affect the response to recall antigens. These data are reviewed extensively in the GSK1070806 Investigator Brochure (GlaxoSmithKline Document Number WM2009/00047/01). Furthermore, only ~25% of the LPS/IL-12 induced upregulation of NK cell activation/differentiation markers (CD69 and CD274) was inhibited by GSK1070806 in the ex-vivo whole blood assay in the FTIH study.

Through its effect on Th1, cytotoxic lymphocytes, and NK cells, IL-18 is hypothesised to potentiate immune surveillance of nascent tumours. Indeed, recombinant human IL-18 (SB485232) immunotherapy is currently being investigated by GSK for treatment of various human cancers in combination with established chemotherapy. In general, this risk may be more pronounced with long-term, chronic antagonism of IL-18.

Given the potential utility of GSK1070806 in treating metabolic diseases, the impact of IL-18 antagonism in obese subjects was assessed in the FTIH study. No significant differences in safety, tolerability, or immunological parameters were observed compared to normal healthy individuals. It has been proposed that subjects with T2DM are more prone to certain infections compared with normal individuals [Shah, 2003]. Furthermore, recent case reports have suggested that morbidly obese subjects with a body mass index (BMI) >40kg/m^2 may be at greater risk of complications from influenza, possibly as a result of existing pulmonary and cardiovascular co-morbidites [Napolitano, 2009]. Hence there is a theoretical increase in risk of compromised immunity in obese subjects with T2DM dosed with GSK1070806.

1.9. Summary of Risk Management

This study is designed to evaluate the efficacy, safety and tolerability of GSK1070806 in obese subjects with T2DM on metformin monotherapy. Morbidly obese subjects with BMI ≥ 40kg/m^2 will be excluded from the study. Single-dose intravenous administration of GSK1070806 has been shown to be well tolerated at doses up to 3mg/kg in obese subjects (BMI range 30-40kg/m^2) and at doses up to 10mg/kg in healthy volunteers during the on-going FTIH. In this planned study two doses will be administered 28 days apart; whilst two doses have not been administered to date, the design, which allows a long observational period following the first dose to monitor any adverse events prior to administration of a second dose is considered appropriate. Furthermore in the preclinical safety study which was conducted in cynomologous monkeys, doses up to 300mg/kg were administered weekly for 4 weeks and were well-tolerated. Based on the half-life of the molecule (26-30 days) significant accumulation of drug following the administration of a second dose is not expected.

Given the potential impact of GSK1070806 on immunity to infection all subjects will be rigorously screened by clinical exam for ongoing or potentially emergent infections by a physician at the screening visit, prior to dosing, and during the course of the study. Subjects who develop clinical signs of an infection during their involvement in the study will be clinically reviewed and samples taken for virology and/or microbiology confirmation of the infection as appropriate and in accordance with local guidelines.

Viral screening methods will be employed at screening. Subjects who test positive for any of the following will be excluded from the study:

- Hepatitis B
- Hepatitis C
- HIV

Subjects with current evidence of acute or ongoing infection, history of repeated, chronic or opportunistic infections, ongoing viral infection [as specified in Section 5.2.3] or history of mycobacterium infection including tuberculosis (TB) will also be excluded. Screening for *Mycobacterium tuberculosis* will be performed at screening and all subjects that test positive will be excluded. Given the hypothetical role of IL-18 in the potentiation of immune surveillance of nascent tumours, subjects with a history of malignancy will also be excluded from the study.

1.9.1. Inclusion of females of child-bearing potential

While the reproductive toxicology package has not been conducted repeat dose pre-clinical safety studies (1 month in duration) with GSK1070806 involving male and female animals did not show any drug-related findings in the reproductive tissues (ovaries, uterus, vagina, cervix, prostate and testes), nor was any binding of GSK1070806 seen with human reproductive tissues (fallopian tube, ovary, placenta, uterus cervix, uterus endometrium, prostate or testis).

Eligible women of childbearing potential (WCBP) will be allowed to participate in the proposed trial at study sites where regulation allows for their inclusion. The recommendations and precautions for preventing WCBP enrolled in the proposed Phase IIa study from becoming pregnant and the procedures to be followed in case of accidental pregnancy are discussed in Section 6.3.1, Section 6.3.2 and Section 6.3.3, respectively.

1.9.2. Follow-up time for use of female contraception

The duration of required contraception for WCBP enrolled in the study is approximately 30 weeks after the first dose or up to study day 210. This time period is based on the observed half life of GSK1070806 from the on-going FTIH study and the time required for the drug levels to decline to a concentration level which has been shown to have limited effect on *ex-vivo* PD parameters.

Based on the observed PK profile from the on-going FTIH study, it will take on average 170 days (Approximately 24 weeks) after the first dose for the exposure levels to fall below 10µg/mL following the administration of a 5 mg/kg dose. The rationale for using a cut-off value of 10µg/mL is based on the emerging preliminary *ex-vivo* PD data (WBA) from the on-going FTIH study; where the administration of a 0.25 mg/kg dose to obese subjects, resulted in limited inhibition of IFNγ. The obese population investigated in the FTIH study was considered more appropriate to base this rationale on as the proposed study will investigate obese T2DM patients.

It is therefore suggested to use the maximum concentration (Cmax) observed at this dose (0.25 mg/kg) in the obese population as the cut-off concentration value for the contraception period follow-up. In the on-going FTIH study, the geometric mean Cmax value achieved after a single dose of 0.25 mg/kg in obese subjects was approximately 10µg/mL.

Table 1 : Pharmacokinetics of GSK1070806

Dose mg/kg	Time (days) from first dose to 10 µg/mL	Nr. of half-lives
5	170	4.7

As these are average predictions, an additional 40 days (Approximately an additional half-life) were added for extra caution. Therefore, a period of 210 days (approximately 30 weeks) after the first dose (or approximately 26 weeks after the last dose) is proposed as the required duration of contraceptive use.

1.9.3. Influenza management

Where possible, inactivated flu vaccine will be administered at screening prior to starting treatment with study medication and in accordance with local practices. The onset of seroprotection for the specific vaccine (as indicated by product labeling), should be taken into consideration. If not administered prior to screening (e.g. if screening occurred before the flu season) and subsequently deemed necessary inactivated flu vaccine may be administered 2 months post the first dose.

If any subject develops signs and symptoms related to influenza (for example body temperature fever (>38°C) or a history of fever, and two or more of the following symptoms: cough, sore throat, runny nose, sneezing, limb / joint pain, headache, vomiting / diarrhoea in the absence of a known cause) during participation in the study, the subject will be treated as per local protocols and tested for Influenza A and B (by RT-PCR) including H1N1. Subjects will be offered anti-viral therapy where possible (e.g Tamiflu or RELENZA™). Each case of infection will be carefully reviewed and assessed for potential relatedness to study drug after consultation between the Sponsor's Medical Monitor, the study Investigator and appropriate specialists. Additionally, if a household member of a subject has the flu, and the subject has not been vaccinated for the flu, the subject should be offered anti-viral therapy where possible (e.g Tamiflu or RELENZA).

1.9.4. Travel restrictions for subjects participating in trial

Subjects will be advised to refrain from travelling to countries where there is a high incidence of infectious diseases until the study follow up visit on Day 210. This is based on the same rationale which was proposed on Section 1.9.3. to determine the follow-up time for use of female contraception. By Day 210 the effects on IFNγ inhibition in the obese population is predicted to be minimal, therefore little or no impact on the subjects immunity to infection would be anticipated after this time period.

2. OBJECTIVE(S) AND ENDPOINTS

**Primary Objective**	**Primary Endpoint**
• To evaluate the efficacy of two repeat intravenous dose administrations of GSK1070806 in subjects with T2DM.	• Change from baseline in fasting plasma glucose and weighted mean glucose AUC (0-4hrs) post-Mixed Meal Test (MMT) on days 29, 57 and 85.
**Secondary Objectives**	**Secondary Endpoints**
• To evaluate the safety and tolerability of two repeat intravenous dose administrations of GSK1070806 in obese subjects with T2DM.	• Safety and tolerability parameters include: adverse events, clinical laboratory tests, electrocardiograms (ECGs), and vital signs.
• To evaluate the effect of two repeat intravenous dose administrations of GSK1070806 on additional markers of efficacy, in obese subjects with T2DM.	• Change from baseline on days 29, 57 and 85 in % HbA1c, fasting blood insulin, and C-peptide levels; change from baseline in weighted mean insulin, and C-peptide levels [AUC (0-4hrs)] post-MMT on days 29, 57 and 85 and derived measures of insulin sensitivity and β-cell function.
• To evaluate the plasma PK of repeat intravenous doses of GSK1070806 in obese subjects with T2DM.	• AUC(0-τ), Cmax, tmax and after the second dose λz and t½.
• To investigate the effect of repeat intravenous doses of GSK1070806 on free and drug bound IL-18 levels (if measurable) in obese subjects with T2DM.	• Serum levels of free IL-18 and drug bound IL-18
• To explore the pharmacodynamic (PD) effect of repeat intravenous doses of GSK1070806 on biomarkers of inflammation and metabolic disease.	• Change from baseline in serum and/or plasma levels of biomarkers of inflammation (e.g. hs-CRP, and IL-6) and metabolic disease (e.g. adiponectin, frucosamine, total cholesterol, high-density lipoprotein (HDL)/low-density lipoprotein (LDL), triglycerides) over 12 weeks.
• To investigate the effect of repeat intravenous doses of GSK1070806 on body composition in obese subjects with T2DM.	• Change from baseline in waist circumference and BMI

Secondary Objectives	Secondary Endpoints
• To assess the potential of anti-GSK1070806 antibody formation following repeat intravenous administration of GSK1070806.	• Incidence and titers of serum of anti-GSK1070806 antibodies
**Exploratory Objectives**	**Exploratory Endpoints**
• To explore the pharmacodynamic (PD) effect of repeat intravenous doses of GSK1070806 on biomarkers of kidney function.	• Changes from baseline in markers of kidney function, e.g., albumin/creatinine ratio (ACR), serum creatinine, monocyte chemoattractant protein (MCP -1) over 12 weeks.
• To explore the effect of repeat intravenous doses of GSK1070806 on cardiovascular function.	• Change from baseline in 24hr blood pressure (BP) on days 29 and 85.
• To explore the PK/PD relationship following repeat intravenous doses administration of GSK1070806 in obese subjects with T2DM.	• Drug bound IL-18 increase in serum, % HbA1c versus cumulative GSK1070806 AUC over 12 weeks and/or change from baseline weighted mean glucose AUC (0-4hrs) post-MMT versus cumulative GSK1070806 AUC on selected time-points up to and possibly including Day 85 if appropriate

3. INVESTIGATIONAL PLAN

3.1. Study Design/ Schematic

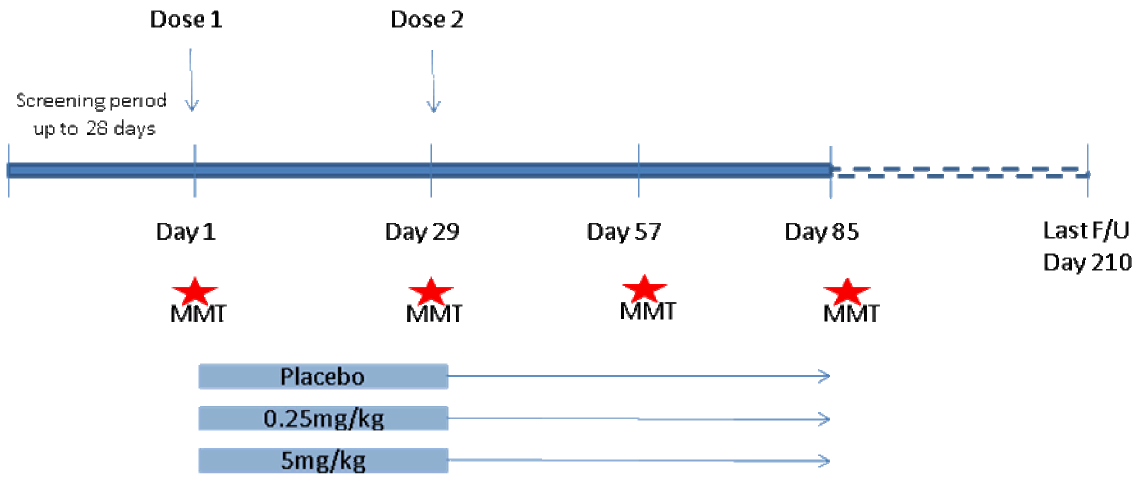

Protocol waivers or exemptions are not allowed with the exception of immediate safety concerns. Therefore, adherence to the study design requirements, including those specified in the Time and Events Table, are essential and required for study conduct.

3.2. Discussion of Design

The proposed study will be a randomised, single-blind (sponsor-unblinded), placebo-controlled, study to investigate the efficacy, safety, tolerability, pharmacokinetics and pharmacodynamics of repeat intravenous infusions (2 doses 4-weeks apart) of GSK1070806 in obese patients with T2DM. The primary objective of the study will be to assess improvements in fasting and postprandial glucose control. This will be a parallel-group study in 30 obese subjects with T2DM who are poorly controlled on metformin monotherapy (HbA1C>7% but ≤9.5%), and who have levels of microalbuminuria indicative of progressive kidney disease i.e. 30-300mg/L albumin in urine <u>or</u> ACR ≥3.5 mg/mmol (female) or ≥2.5 mg/mmol (male) and ≤30mg/mmol. There will be three treatment groups comprising two active and one placebo arm with 10 subjects per dose group.

The study contains a broad range of biomarker assessments, the purpose of which is to evaluate the mechanistic basis by which GSK1070806 exerts its therapeutic benefit in subjects with T2DM. Various hypotheses for these mechanisms have been proposed based on published literature and data from the on-going FTIH study A18110040.

The study consists of screening and a treatment period (4 weeks, sponsor unblinded, placebo-controlled). Details of the blinding status for individuals are provided in Table 2.

Table 2 Summary of blinding status of personnel

Role	Blinded
Medical Monitor	N
Therapeutic Area Representative	N
Study Operations Scientist	N
GCSP Representative	N
Pharmacokineticist	N
Data Manager	N
Statistician	N
Programmer(s)	N
Site Pharmacist	N
All Other Site Staff	Y
Site Monitors	Y

Subjects will be randomised into one of the three treatment groups where they will receive two intravenous infusions of GSK1070806 or placebo twenty-eight days apart. A MMT challenge will be conducted on Day 1, Day 29, Day 57 and Day 85 for evaluation of the primary endpoints.

3.3. Treatment Assignment

Subjects will be assigned equally to placebo, 0.25 mg/kg GSK1070806, or 5 mg/kg GSK1070806 in accordance with the randomization schedule generated by Biopharm Clinical Pharmacology and Biometrics, prior to the start of the study, using validated internal software. Subjects will remain on their current diet and dose of metformin.

3.4. Investigational Product and Other Study Treatment Dosage/Administrations

Table 3 Summary of investigational product

	Study Treatment	
**Product name:**	GSK1070806	Placebo
**Dosage form:**	Intravenous infusion	Intravenous infusion
**Unit dose strength(s)/Dosage level(s):**	0.25 & 5 mg/kg	Matching volume of 0.9% saline to active dose
**Route/ Administration/ Duration:**	Intravenous infusion/ 60 min	Intravenous infusion/ 60 min
**Dosing instructions:**	Days 1 and 29 study visits	Days 1 and 29 study visits
**Device:**	IV infusion pump	IV infusion pump
**Manufacturer/ source of procurement:**	GSK	Baxter

3.4.1. Dose Adjustment/Stopping Safety Criteria

3.4.1.1. Liver Chemistry Stopping Criteria

Liver chemistry threshold stopping criteria have been designed to assure subject safety and to evaluate liver event etiology (in alignment with the Food and Drug Administration (FDA) premarketing clinical liver safety guidance).

Study treatment will be stopped if any of the following liver chemistry stopping criteria is met:

1. ALT ≥ 3xULN and total bilirubin ≥ 2xULN (>35% direct bilirubin*); or
   ALT ≥ 3xULN and INR** > 1.5

   *serum bilirubin fractionation should be performed if testing is available; if unavailable, withdraw subject (if ALT ≥ 3xULN and total bilirubin ≥ 2xULN) and measure urinary bilirubin via dipstick.

   ** INR testing not required per protocol and the threshold value does not apply to subjects receiving anticoagulants..

2. ALT ≥ 5xULN.

3. ALT ≥ 3xULN if associated with symptoms (new or worsening) believed to be related to hepatitis (such as fatigue, nausea, vomiting, right upper quadrant pain or tenderness or jaundice) or believed to be related to hypersensitivity (such as fever, rash or eosinophilia).
4. ALT ≥ 3xULN persists for ≥ 4 weeks.
5. ALT ≥ 3xULN and cannot be monitored weekly for 4 weeks.

Subjects with ALT ≥ 3xULN **and** < 5xULN **and** bilirubin < 2xULN, who do not exhibit hepatitis symptoms or rash, can continue study treatment as long as they can be monitored weekly for 4 weeks. See Section 12 for details on weekly follow-up procedures for these subjects.

Refer to Section 12, Liver Chemistry Follow-up Procedures, for details of the required assessments if a subject meets any of the above criteria.

3.4.1.2. QTc Withdrawal Criteria

A subject that meets the criteria below will be withdrawn from the study. The QT correction formula used to determine discontinuation should be the same one used throughout the study.

- QTc, QTcB, QTcF > 500 msec or uncorrected QT >600 msec
- If subject has underlying bundle branch block then the QTc withdrawal criteria depends on the baseline value:

Baseline QTc value (with underlying bundle branch block)	QTc withdrawal criteria
<450 ms	>500 ms
450-480 ms	≥530 ms

Withdrawal decisions are to be based on an average QTc value of triplicate ECGs. If an ECG demonstrates a prolonged QT interval, obtain 2 more ECGs over a brief period, and then use the averaged QTc values of the 3 ECGs to determine whether the subject should be discontinued from the study.

3.4.1.3. Blood Glucose Withdrawal Criteria

Hyperglycemia

Although subjects should remain on their stable dose of prescribed metformin treatment, there is a small risk that glucose control will deteriorate during the course of the clinical trial. Subjects will be required to measure glucose levels three days per week, once before breakfast then again 2hrs after breakfast, from randomization until Day 85 and record results in a subject diary card. If subjects have a morning fasting blood glucose concentration above 13.3mmol/L (240mg/dL) for 3 consecutive days and this is confirmed with a venous sample, they will be offered standard of care at the discretion of the investigator, in consultation with the medical monitor. Subjects will continue to be monitored out to the end of the study as planned.

Hypoglycemia

Although medications that lower blood glucose are capable of producing hypoglycemia, symptoms of hypoglycemia are generally observed in subjects treated with medications that augment insulin secretion. This manifestation of hypoglycemia is variable among subjects with diabetes. In addition to the symptoms that may occur in association with a low blood glucose levels, some diabetics may experience symptoms with a rapid lowering or improvement in their blood glucose control. In contrast, some subjects with diabetes with a very low glucose level may be unaware or asymptomatic.

Whilst hypoglycaemia is not expected in this study because both metformin and GSK1070806 should impact on insulin resistance alone, subjects with frequent hypoglycemia will be withdrawn from the study at the discretion of the investigator, in consultation with the medical monitor. Subjects experiencing severe hypoglycemia (refer to Appendix 4) will be withdrawn.

3.5. Time and Events Table

	Screening (up to 28 days)	Day 1				Day 4	Day 9	Day 14	Day 21	Day 29				Day 32	Day 42	Day 57	Day 85	Day 120 (approx)	Day 165 (approx)	Day 210 (approx)
		Pre-dose	Dose	1hr post-dose	4hrs post dose					Pre-dose	Dose	1hr post-dose	4hrs post dose							
Informed consent	X																			
GSK1070806 or placebo			X								X									
Medical history	X																			
Pregnancy test for females	X	X								X						X	X			X
Adverse events					X	X	X	X	X	X			X	X	X	X	X	X	X	X
Serious adverse event	X	X			X	X	X	X	X	X			X	X	X	X	X	X	X	X
Concomitant meds	X	X				X	X	X	X	X				X	X	X	X	X	X	X
Full physical examination	X																			
Brief physical exam		X								X										
Drugs of abuse and alcohol tests	X																			
Vital signs (BP, HR, RR, body temp) – supine	X	X			X	X	X	X	X	X			X	X	X	X	X	X	X	X
End of 24hr BP monitoring[1]		X								X							X			
Serology: Hep B and C, HIV, FSH & estradiol (WNCBP)	X																			
Influenza screening	X																			
Vaccination (flu/pneumococcus)	X																			
QuantiFERON TB test	X																			
12-lead ECG	X	X		X	X					X		X	X							
BMI and waist circumference	X	X															X			

	Screening (up to 28 days)	Day 1				Day 4	Day 9	Day 14	Day 21	Day 29				Day 32	Day 42	Day 57	Day 85	Day 120 (approx)	Day 165 (approx)	Day 210 (approx)
		Pre-dose	Dose	1hr post-dose	4hrs post dose					Pre-dose	Dose	1hr post-dose	4hrs post dose							
Clinical Labs including Hematology & Chemistry	X	X			X	X	X	X	X	X			X	X	X	X	X	X	X	X
Mixed Meal Test (breakfast)		X								X						X	X			
Blood sample for PK		X		X	X	X		X		X		X	X	X	X	X	X	X		X
Blood sample for HbA1c	X	X								X						X	X	X		X
Blood sample for immunogenicity		X								X						X	X	X		X
Fasted blood samples for biomarkers		X								X						X	X			X
Blood sample for Free IL-18, IL-18 drug complex		X		X		X		X		X		X		X	X	X	X	X		X
Blood sample for pharmacogenetics		X																		
Urine sample for albumin and biomarker assessments	X	X								X						X	X			
Outpatient visit	X	X				X	X	X	X	X				X	X	X	X	X	X	X

1. Monitoring to start 24hrs before clinic visit

4. STUDY POPULATION

4.1. Number of Subjects

Approximately 35 subjects will be enrolled such that approximately 30 subjects complete dosing and critical assessments.

If subjects prematurely discontinue the study, additional subjects may be enrolled as replacement subjects and assigned to the same treatment sequence at the discretion of the Sponsor in consultation with the investigator.

4.2. Eligibility Criteria

4.2.1. Inclusion Criteria

Specific information regarding warnings, precautions, contraindications, adverse events, and other pertinent information on the GSK investigational product or other study treatment that may impact subject eligibility is provided in the Clinical Investigator's Brochure (CIB, GlaxoSmithKline Document Number WM2009/00047/01).

Deviations from inclusion criteria are not allowed because they can potentially jeopardize the scientific integrity of the study, regulatory acceptability or subject safety. Therefore, adherence to the criteria as specified in the protocol is essential.

A subject will be eligible for inclusion in this study only if all of the following criteria apply.

1. A diagnosis of T2DM as determined by a responsible physician based on a medical evaluation including medical history, physical examination, and laboratory tests, with onset at least 6 months prior to Screening. Subjects may be entered if they have stable hypertension or dyslipidemia on therapy, provided there is no change in either the type or dose of medications during the 3-month period before the study. Subjects with other conditions except as noted in the Exclusion criteria may be included only if the investigator and GSK medical monitor agree that the condition is unlikely to introduce additional risk factors and will not interfere with study procedures.
2. Male or female between 18 and 70 years of age inclusive, at the time of signing the informed consent.
3. HbA1c levels ≥ 7.0 % and ≤ 9.5%; at Screening.
4. On a stable dose of monotherapy with metformin for three months prior to screening, and at a total daily dose greater than or equal to 1000 mg for at least 2 months prior to dosing.
5. Fasting plasma glucose level < 13.3 mmol/L (240 mg/dL) at screening.
6. Obese with BMI ≥ 30 kg/m^2, and < 40 kg/m2.
7. Presence of microalbuminuria: 30-300mg/L albumin in urine or Albumin Creatinine Ratio (ACR) ≥ 3.5 mg/mmol (female) or ≥2.5 mg/mmol (male) and ≤ 30 mg/mmol (female and male)..

8. The subject is capable of giving written informed consent, which includes compliance with the requirements and restrictions listed in the consent form.
9. A female subject is eligible to participate if she is of:
   - Non-childbearing potential defined as pre-menopausal females with a documented tubal ligation or hysterectomy; or postmenopausal defined as 12 months of spontaneous amenorrhea [in questionable cases a blood sample with simultaneous follicle stimulating hormone (FSH) > 40 MlU/ml and estradiol < 40 pg/ml (<140 pmol/L) is confirmatory]. Females on hormone replacement therapy (HRT) and whose menopausal status is in doubt will be required to use one of the contraception methods in the full protocol if they wish to continue their HRT during the study. Otherwise, they must discontinue HRT to allow confirmation of post-menopausal status prior to study enrollment. For most forms of HRT, at least 2-4 weeks will elapse between the cessation of therapy and the blood draw; this interval depends on the type and dosage of HRT. Following confirmation of their post-menopausal status, they can resume use of HRT during the study without use of a contraceptive method.
   - Child-bearing potential (**only applicable for sites where regulation allows for their inclusion**) and agrees to use one of the contraception methods listed in Section 7.1. Acceptable contraception is required by WCBP for one month prior to screening, during the course of the study and 210 days after the first dose of study drug.
10. Male subjects must agree to use one of the contraception methods listed in Section 7.1.2. This criterion must be followed from the time of the first dose of study medication until after the last follow-up visit at Day 210.
11. ALT < 2xULN; alkaline phosphatase and bilirubin ≤ 1.5xULN (isolated bilirubin >1.5xULN is acceptable if bilirubin is fractionated and direct bilirubin <35%).
12. Single or Average QTc, QTcB or QTcF < 450 msec; or QTc < 480 msec in subjects with Bundle Branch Block.

4.2.2. Exclusion Criteria

Deviations from exclusion criteria are not allowed because they can potentially jeopardize the scientific integrity of the study, regulatory acceptability or subject safety. Therefore, adherence to the criteria as specified in the protocol is essential.

A subject will not be eligible for inclusion in this study if any of the following criteria apply:

1. Current evidence, or history within the last 7 days, of an influenza-like illness as defined by fever (>38°C) and two or more of the following symptoms: cough, sore throat, runny nose, sneezing, limb / joint pain, headache, vomiting / diarrhoea in the absence of a known cause, other than influenza.

2. Use of anti-inflammatory drugs including corticosteroids, chronic maintenance therapy with NSAIDs, anti-Tumor Necrosis Factor (anti-TNF) or anti-Interleukin-1 (anti-IL1) within 60 days prior to dosing. NSAIDs are permitted for intermittent use during the course of the study.
3. Current evidence of ongoing or acute infection, history of repeated, chronic or opportunistic infections (e.g. recurrent folliculitis, other cutaneous infections or repeated pneumonia) or history of a serious bacterial infection within 6 months of randomisation.
4. History of malignancy or significant cardiac, pulmonary, metabolic, renal, hepatic, or gastrointestinal conditions that in the opinion of the investigator and/or GSK Medical Monitor, places the subject at an unacceptable risk as participant in this trial.
5. History chronic granulomatous infections, such as of Mycobacterium tuberculosis or any other previous Mycobacterium infection.
6. Creatinine clearance less than 60ml/min
7. Screens positive of Hepatitis B surface antigen, Hepatitis C antibody or Human Immunodeficiency Virus (HIV)
8. History of a severe allergic reaction, anaphylaxis or immunodeficiency.
9. Current or chronic history of liver disease, or known hepatic or biliary abnormalities (with the exception of Gilbert's syndrome or asymptomatic gallstones).
10. A positive pre-study drug/alcohol screen.
11. History of regular alcohol consumption within 6 months of the study defined as:

    An average weekly intake of >21 units for males or >14 units for females. One unit is equivalent to 8 g of alcohol: a half-pint (~240 ml) of beer, 1 glass (125 ml) of wine or 1 (25 ml) measure of spirits.

12. The subject has participated in a clinical trial and has received an investigational product within the following time period prior to the first dosing day in the current study: 30 days, 5 half-lives or twice the duration of the biological effect of the investigational product (whichever is longer).
13. Exposure to more than four new chemical entities within 12 months prior to the first dosing day.
14. Unable to refrain from the use of prescription or non-prescription drugs, including vitamins, herbal and dietary supplements (including St John’s Wort) within 7 days (or 14 days if the drug is a potential enzyme inducer) or 5 half-lives (whichever is longer) prior to the first dose of study medication, unless in the opinion of the Investigator and GSK Medical Monitor the medication will not interfere with the study procedures or compromise subject safety.
15. History of sensitivity to any of the study medications, or components thereof or a history of drug or other allergy that, in the opinion of the investigator or GSK Medical Monitor, contraindicates their participation.
16. Where participation in the study would result in donation of blood or blood products in excess of 500 mL within a 56 day period.

17. Pregnant females as determined by positive serum or urine hCG test at screening.
18. Lactating females.
19. Unwillingness or inability to follow the procedures outlined in the protocol.
20. Subject is mentally or legally incapacitated.
21. Subject has received a live attenuatedvaccine(s) within 30 days of randomisation or will require vaccination with a live attenuated vaccine prior to the end of the study.

4.3. Screen and Baseline Failures

Data for screen and baseline failures will be collected in source documentation at the site but will not be transmitted to GSK.

5. DATA ANALYSIS AND STATISTICAL CONSIDERATIONS

5.1. Hypotheses and Treatment Comparisons

Precision Estimation

This study is designed to estimate the effect of 0.25 mg/kg GSK1070806 and 5 mg/kg GSK1070806 relative to placebo on:

- Change from baseline in fasting plasma glucose levels
- Change from baseline in weighted mean AUC(0-4hrs) post-MMT profiles for glucose in obese subjects with T2DM currently on metformin monotherapy with HbA1c >7%.

No formal hypothesis will be tested. Point estimates and corresponding 95% confidence intervals will be constructed for the difference between the mean of the test treatment and the mean of the reference treatment, μ(test) - μ(reference).

Supportive analyses in the Bayesian framework, assuming a non-informative prior, may also be explored for key primary and secondary endpoints Posterior probabilities and 95% credible intervals for the difference between the mean effect of the test treatment and the mean effect of the reference treatment (μ(test) - μ(reference)) being greater than treatment differences of interest may be provided.

5.2. Sample Size Considerations

5.2.1. Sample Size Assumptions

There are no formal calculations of power or sample size for this study. The sample size has been selected, to allow preliminary characterisation of efficacy, safety and tolerability in obese subjects with T2DM, and to investigate the effect on PD assessment and biomarkers.

A sufficient number of subjects (male and female with T2DM currently on metformin monotherapy with HbA1c in the 7-9.5% range) will be recruited into the study such that approximately 36 subjects are randomised to placebo or GSK1070806 to achieve 30 evaluable subjects.

GSK studies SGLT-2 [GlaxoSmithKline Document Number RM2006/00553/00] and SGLT-1 inhibitors [GlaxoSmithKline Document Number GM2007/00045/09] have demonstrated that detectable changes in FPG and 2h/24h glucose profiles with 10 randomised subjects is achievable.

Based on the data from SGA112534 and KG2104940, estimates of the square root of the mean square errors for change from baseline FPG and weighted mean glucose AUC(0-4) are 23.0mg/dL and 32.8mg/dL, respectively. Expected 95% CI widths for mean difference of plasma glucose between treatment and placebo have been calculated assuming that 10 subjects with evaluable data completed each active arm and into the combined placebo group.

Parameter	SD	95% CI Half Width
Change FPG	27.0	25.0
AUC(0-4)	32.8	30.4

Assuming 10 evaluable subjects per arm, and given the estimates of standard deviation above and using two-sided two-sample t test, this study will provide 80% power to detect a difference in change from baseline FPG of 35.8 mg/dL (1.99 mmol/L) and a difference in change from baseline weighted mean AUC(0-4) of 45.8 mg/dL (2.54 mmol/L).

Although differences as low as 30 mg/dL (1.66 mmol/L) for both FPG and weighted mean AUC(0-4) may be of clinical significance, but the study is not powered to detect changes of this magnitude.

5.2.2. Sample Size Sensitivity

No sample size sensitivity analyses will be performed.

5.2.3. Sample Size Re-estimation

No sample size re-estimation will be performed.

5.3. Data Analysis Considerations

5.3.1. Interim Analysis

<u>During The Study</u>

There will be ongoing data reviews conducted by the study team of the unblinded safety, pharmacokinetics and biomarker/pharmacodynamic data throughout the trial progression.

Interim Analysis: Day 57

Details of the formal interim analysis are outlined below:

- The interim analysis will occur when approximately 30 patients have completed Day 57 of the study. As appropriate, available data for subjects completing post Day 57 may also be included.
- The purpose of this interim analysis is to provide the project team and GSK stakeholders with key data to inform internal decision making and in order to plan future studies within the clinical development for the asset.
- Appropriate data summaries will be at the individual patient and treatment group level for key endpoints of interest and the circulation of results will be restricted to selected members of the project team and key GSK stakeholders. Results will not be circulated or discussed with staff involved in the conduct of the study at the sites.
- It is not expected that this review will occur on a fully clean database.
- There are no planned implications for the conduct of the study.
- Full details of planned interim analysis will be included in the reporting and analysis plan (RAP).

Interim Analysis: Day 85

It may also be necessary to conduct a formal interim analysis once all of the Day 85 data are available for all subjects to enable the data from the primary endpoint to be reported within 8 month of the primary completion date in accordance with GSK's data disclosure guidelines (POL-GSKF-408).

Full details of planned interim analysis will be included in the RAP.

5.3.2. Final Analyses

The final planned analyses will be performed after all subjects have completed the study and after database freeze/unblinding

5.3.3. Safety Analyses

Safety data will be presented in tabular and/or graphical format and summarized descriptively according to GSK's Integrated Data Standards Library (IDSL) standards.

5.3.3.1. Pharmacokinetic Analyses

Raw Plasma Concentrations

Blood sampling time will be related to the start of the infusion procedure.

Linear and semi-logarithmic individual plasma concentration-time profiles and mean and median profiles by GSK1070806 dose will be plotted.

Plasma concentrations of GSK1070806 will be listed and summarised by dose and nominal time.

Derived Plasma Pharmacokinetic Parameters

Pharmacokinetic analysis will be the responsibility of the Clinical Pharmacology Modeling and Simulation Department, QSci, GSK.

Plasma concentration time data for GSK1070806 will be analyzed by non-compartmental methods according to GlaxoSmithKline guidance document, GUI-51487 and using WinNonlin. Calculations will be based on the actual sampling times recorded during the study.

Where data permits, the following pharmacokinetic parameters will be determined from the plasma concentration-time data for each dose of GSK1070806 for each subject:

AUC(0-τ), Cmax, tmax and after the second dose λz, the number of points used to determine λz, terminal phase half life (t1/2) as well as the accumulation ratio.

Pharmacokinetic data will be presented in graphical and/or tabular form and will be summarized descriptively. All pharmacokinetic data will be stored in the Archives, GlaxoSmithKline Pharmaceuticals, R&D.

Statistical analyses of the pharmacokinetic parameter data will be the responsibility of Biopharm Clinical Pharmacology & Biometrics, GlaxoSmithKline.

5.3.3.2. Pharmacokinetic/Pharmacodynamic Analyses

Exploratory plots will be presented for individual and/or pooled plasma GSK1070806 concentrations versus serum free and/or drug bound IL-18 levels, if appropriate. If data permits, further PK/PD modelling will be performed using an indirect response model. Exploratory plots will be presented for % HbA1c versus cumulative GSK1070806 AUC over 12 weeks and/or change from baseline weighted mean glucose AUC (0-4hrs) post-MMT versus cumulative GSK1070806 AUC on selected time-points up to and possibly including Day 85. If more appropriate an exploratory plot for plasma glucose concentrations versus GSK1070806 plasma concentrations will be presented.

If deemed appropriate, further PK/PD modelling might be performed based on the results of the exploratory graphical analysis showing obvious relationships or trends between % HbA1c versus cumulative GSK1070806 AUC over 12 weeks and/or change from baseline weighted mean glucose AUC (0-4hrs) post-MM versus cumulative

GSK1070806 AUC on selected time-points up to and possibly including Day 85 or if more appropriate between plasma glucose concentrations versus GSK1070806 plasma concentrations. The choice of the structural pharmacokinetic / pharmacodynamic model will be dependent on the emerging data. More details of any exploratory pharmacokinetic/pharmacodynamic analysis will be provided in the Report and Analysis Plan (RAP). In addition, for the other biomarker variables/PD endpoints, summary tables will be reviewed to identify those biomarker variables/PD endpoints were there is a potential trend. If there is a trend then exploratory plots will be presented for individual and/or pooled plasma GSK1070806 concentrations versus corresponding biomarker variables/PD endpoints. If deemed appropriate, further PK/PD modelling might be performed on those biomarker variables/PD endpoints selected based on the results of the exploratory graphical analysis showing obvious relationships or trends between concentration and biomarker variables/PD endpoints. The choice of the structural pharmacokinetic/pharmacodynamic models will be dependent on the emerging data. More details of any exploratory pharmacokinetic/pharmacodynamic analyses will be provided in the RAP.

5.3.3.3. Pharmacodynamic/Biomarker Analyses

Pharmacodynamic/biomarker parameters will be descriptively summarised, graphically presented and listed by each dose regimen. The pre-dose measure will be used as the baseline measure for change from baseline derivations.

Change from baseline in fasting plasma glucose levels will be compared between treatment groups using repeated measures analysis with fixed effects for baseline, visit and baseline by visit. Visit will be considered a repeated measures factor within subject, where subject will be considered a random effect. The adjusted means, pairwise treatment differences, p-values and 95% confidence limits for the treatment differences will be presented.

For all formal PD analyses, untransformed data will be analysed, unless otherwise specified. For each endpoint, $\log_e$-transformed analyses will be conducted only if after examination of residuals of the untransformed analysis it is determined that the model assumptions have not been met.

Weighted means AUC(0-4hrs) post-MMT profiles for glucose will be calculated using the linear trapezoidal method. In order for the AUC to be calculated, the first and last time points and at least one additional assessment falling between the two measurements has to be non-missing.

Change from baseline in weighted means AUC(0-4hrs) post-MMT profiles for glucose, will be compared between treatment groups using repeated measures analysis with fixed effects for baseline, visit and baseline by visit. Visit will be considered a repeated measures factor within subject, where subject will be considered a random effect. The adjusted means, pairwise treatment differences, p-values and 95% confidence limits for the treatment differences will be presented.

Supportive analyses in the Bayesian framework, assuming a non-informative prior, may also be explored. Posterior probabilities and 95% credible intervals for the difference between the mean effect of the test treatment and the mean effect of the reference treatment (μ(test) - μ(reference)) being greater than treatment differences of interest may be provided. A more detailed description of descriptive summaries and analyses for all primary and secondary endpoints will be provided in the RAP.

5.3.3.4. Exploratory Biomarker(s) Analyses

The results of exploratory biomarker investigations may be reported separately from the main clinical study report. All endpoints of interest from all comparisons will be descriptively and/or graphically summarized as appropriate to the data.

Additional exploratory analyses may be performed to further characterize the novel biomarker.

6. STUDY ASSESSMENTS AND PROCEDURES

This section lists the parameters of each planned study assessment. The exact timing of each assessment is listed in the Time and Events Table (Section 3.5). Detailed procedures for obtaining each assessment are provided in the Study Procedures Manual (SPM). Whenever vital signs, 12-lead ECGs and blood draws are scheduled for the same nominal time, the assessments should occur in the following order: 12-lead ECG, vital signs, blood draws, so that the timing of the assessments should allow the blood draw to occur at the exact nominal time.

The timing and number of planned study assessments, including safety, PK, biomarker, immunogenicity , free IL-18 and IL-18 drug complex and urine assessments may be altered during the course of the study based on newly available data (e.g. to obtain data closer to the time of peak plasma concentrations) to ensure appropriate monitoring. The change in timing or addition of time points for any planned study assessments must be approved and documented by GSK, but this will not constitute a protocol amendment. The Institutional Review Board (IRB)/ Independent Ethics Committee (IEC) will be informed of any safety issues that require alteration of the safety monitoring scheme. No more than 550mL of blood will be collected over the duration of the study, including any extra assessments that may be required.

6.1. Demographic/Medical History Assessments

Subjects will be screened within 4 weeks prior to administration of study medication to confirm that they meet the entrance criteria for the study.

The study investigator or a sub-investigator will discuss with each subject the nature of the study, its requirements, and its restrictions. Written informed consent must be obtained prior to performance of any protocol-specific procedures.

Standard subject demographic data will be obtained and will include subject age, weight (kg), height (cm), sex, race and ethnicity.

Values for the laboratory parameters should be within the normal range for the relevant subject population as specified by the clinical laboratory, which will be an accredited facility. Subjects with screening laboratory values outside of the normal range will be accepted into the study only after the Investigator, or a qualified designee, and the GSK Medical Monitor have determined that the out of range value(s) are not clinically significant and would not pose an increased risk to the subject.

Medical/medication/alcohol history will be assessed as related to the eligibility criteria listed in Section 4.2. Cardiovascular medical history/risk factors will also be assessed at baseline.

6.2. Safety

Planned timepoints for all safety assessments are listed in the Time and Events Table (Section 3.5). Additional time points for safety tests such as vital signs, physical exams and laboratory safety tests may be added during the course of the study based on newly available data to ensure appropriate safety monitoring.

6.2.1. Physical Exams

A complete physical exam will be performed at screening. A brief physical exam will be performed on Day 1 (prior to the first dosing) and Day 29 (prior to the second dosing and on early withdrawal.

- A complete physical examination will include assessments of the head, eyes, ears, nose, throat, skin, thyroid, neurological, lungs, cardiovascular, abdomen (liver and spleen), lymph nodes and extremities. Height and weight will also be measured and recorded.
- A brief physical examination will include assessments of the skin, lungs, cardiovascular system, and abdomen (liver and spleen).

6.2.2. Vital Signs

Vital sign measurements will include systolic and diastolic BP, heart rate (HR), respiratory rate (RR) and body temperature. These assessments will be evaluated at screening, and at various time points outlined in the Time and Events Tables (Section 3.5) or on early withdrawal. On the dosing days (1 and 29), vital sign measurements will be performed at pre- and post dose. Single assessments will be performed on all other days prior to breakfast. All vital sign measurements will be made with the subject in a supine position having rested in this position for at least 5 minutes before each reading.

6.2.3. BP monitoring

BP will be monitor for 24 hours in all subjects prior to each dosing occasion and at the Day 85 visit.

Ambulatory Blood Pressure Monitoring (ABPM) will be used for this assessment. While the subject’s blood pressure is being measured, they can move around and have a normal daily life. The test is normally carried over 24 hours and uses a small digital blood pressure machine that is attached to a belt around subject’s upper arm. Further details can be found in the SPM.

6.2.4. Electrocardiogram (ECG) Monitoring

12-lead ECGs will be obtained at each time point in Section 3.5 of Time & Event table during the study and upon early subject withdrawal using an ECG machine that automatically calculates the heart rate and measures PR, QRS, QT, and QTc intervals. The value measured on pre-dose on Day 1 will be recorded as the baseline value. ECG measurements will be made with the subject in a supine position having rested in this position for at least 5 minutes before the reading is taken. Refer to Section 3.4.1.2 for QTc withdrawal criteria and additional QTc readings that may be necessary.

6.2.5. Clinical Laboratory Assessments

Subjects will be required to fast from midnight prior to collection of blood samples for clinical laboratory testing. Samples will be collected as outlined in the Time and Events Tables (Section 3.5) and upon early subject withdrawal.

At the Principal Investigator and GSK Medical Monitor's discretion, additional laboratory tests other those outlined below may be included to further assess safety and tolerability.

Hematology, clinical chemistry, urinalysis and additional parameters to be tested are listed below:

Hematology

Platelet Count	*RBC Indices*:	*Automated WBC Differential:*
RBC Count	MCV	Neutrophils
WBC Count (absolute)	MCH	Lymphocytes
Hemoglobin	MCHC	Monocytes
Hematocrit		Eosinophils
		Basophils

Clinical Chemistry (all samples will be fasted)

BUN	Potassium	AST (SGOT)	Total and direct bilirubin
Creatinine	Chloride	ALT (SGPT)	Uric acid
Glucose	Calcium	GGT	Albumin
Sodium		Alkaline phosphatase	Total Protein

Routine Urinalysis

Specific gravity
pH, glucose, protein, blood and ketones by dipstick
Microscopic examination (if blood, nitrite or protein is detected), includes RBC, WBC, casts and bacteria

Other screening tests

HIV
Hepatitis B (HBsAg)
Hepatitis C (Hep C antibody -- if second generation Hepatitis C antibody positive, a hepatitis C antibody Chiron RIBA immunoblot assay (or other third generation immunoassay) should be reflexively performed **on the same sample** to confirm the result)
Mycobacterium Tuberculosis – using QuantiFERON Gold Test
FSH and estradiol (as needed in women of non-child bearing potential only)
Alcohol and drug screen (to include at minimum: amphetamines, barbiturates, cocaine, opiates, cannabinoids and benzodiazepines).
HbA1c
Urine microalbuminuria or ACR

A breath alcohol and drug tests may be substituted for urine alcohol testing at the discretion of the study site. Additional drug screens may be performed at the discretion of site staff if needed.

6.3. Pregnancy

6.3.1. Time period for collecting pregnancy information

All pregnancies in female subjects and/or female partners of male subjects will be collected after the start of dosing and until the plasma levels of GSK1070806 are predicted on average to be approximately 10.0µg/mL from when the first dose is administered. As this is a single-blind (sponsor-unblinded) study the follow-up time will be based on the 5 mg/kg dose group which represents the worst case scenario and will be the day 210 follow up.

6.3.2. Action to be taken if pregnancy occurs

The investigator will collect pregnancy information on any female subject, who becomes pregnant while participating in this study. The investigator will record pregnancy information on the appropriate form and submit it to GSK within 2 weeks of learning of a subject's pregnancy. The subject will also be followed to determine the outcome of the pregnancy. Information on the status of the mother and child will be forwarded to GSK. Generally, follow-up will be no longer than 6 to 8 weeks following the estimated delivery date. Any premature termination of the pregnancy will be reported.

While pregnancy itself is not considered to be an AE or SAE, any pregnancy complication or elective termination of a pregnancy for medical reasons will be recorded as an AE or SAE.

A spontaneous abortion is always considered to be an SAE and will be reported as such. Furthermore, any SAE occurring as a result of a post-study pregnancy and is considered reasonably related to the study treatment by the investigator, will be reported to GSK as described in Section 11. While the investigator is not obligated to actively seek this information in former study participants, he or she may learn of an SAE through spontaneous reporting.

Any female subject who becomes pregnant while participating will discontinue study medication and be withdrawn from the study.

6.3.3. Action to be taken if pregnancy occurs in a female partner of a male study subject

The investigator will attempt to collect pregnancy information on any female partner of a male study subject who becomes pregnant while participating in this study. The investigator will record pregnancy information on the appropriate form and submit it to GSK within 2 weeks of learning of the partner's pregnancy. The partner will also be followed to determine the outcome of the pregnancy. Information on the status of the mother and child will be forwarded to GSK. Generally, follow-up will be no longer than 6 to 8 weeks following the estimated delivery date. Any premature termination of the pregnancy will be reported.

6.4. Pharmacokinetics

6.4.1. Blood Sample Collection

Blood samples for pharmacokinetic analysis of GSK1070806 will be collected at the time points indicated in Section 3.5, Time and Events Table. The actual date and time of each blood sample collection will be recorded. The timing of PK samples may be altered and/or PK samples may be obtained at additional time points to ensure thorough PK monitoring.

A PK sample will be collected at the time of the immunogenicity sample visit (see Section 3.5, Time and Events Table).

Details of PK blood sample collection (including volume to be collected), processing, storage and shipping procedures are provided in the Study Procedures Manual (SPM).

6.4.2. Sample Analysis

Plasma analysis will be performed under the management of Bioanalytical Science and Toxicokinetics, DMPK, GlaxoSmithKline. Concentrations of GSK1070806 will be determined in plasma samples using the currently approved analytical methodology. Raw data will be stored in the Good Laboratory Practice (GLP) Archives, GlaxoSmithKline. Once the plasma has been analyzed for GSK1080706, any remaining plasma may be stored in DMPK for further analysis if necessary and the results reported under a separate DMPK protocol.

6.5. Anti-GSK1070806 Antibodies

6.5.1. Blood Sample Collection

Serum samples for testing antibodies against GSK1070806 will be collected as described in the Time and Events Table (Section 3.5).

6.5.2. Sample Analysis

The presence of anti-GSK1070806 binding antibodies will be assessed using an immunoelectrochemi-luminescent (ECL) assay. If sera contain potential anti-GSK1070806 antibodies, they will be further analyzed for the specificity and titres. The results of anti-GSK1070806 antibody tests will be reported at the end of the study. The reports will include incidence of the confirmed positive with titres. Details of the ECL assays and sample processing will be given in the SPM.

6.6. Biomarker(s)

6.6.1. Inflammatory and Metabolic Biomarkers

Fasted blood sample(s) will be collected for measurement of biomarkers of inflammation and metabolic disease. These samples will be used to measure relevant biomarkers such as those provided in this protocol (e.g. total cholesterol, HDL and LDL cholesterol, triglycerides, HbA1c, fructosamine, adiponectin, glucose, and hsCRP). Further details can be found in the SPM.

6.6.2. Exploratory Biomarker Analysis

Samples collected for biomarker analyses during this study may be used for the purposes of measuring exploratory biomarkers to identify factors that may influence T2DM, and/or medically related conditions and/or the response to treatment with GSK1070806. If relevant, this approach will be extended to include the identification of biomarkers associated with adverse events. Biomarker samples will be collected as indicated in the Times and Events table. Limited biomarker testing will be conducted on day 210. The timing of the collections may be adjusted on the basis of emerging PK or PD data from this study or other new information in order to ensure optimal evaluation of the PD endpoints. Further details can be found in the SPM.

6.6.3. Biomarkers in urine

Samples will be taken to analyse the levels of albumin and creatinine. Additional exploratory biomarkers may also be assessed. The timing of the collections may be adjusted on the basis of emerging PK or PD data from this study or other new information in order to ensure optimal evaluation of the PD endpoints. Further details can be found in the SPM.

6.7. Free and drug-bound IL-18 levels in serum

Serum samples for the measurement of free and drug-bound IL-18 will be collected at the time points indicated in the Time and Events Table (Section 3.5). At Days 1 and 29, serum IL-18 samples will be taken pre-dose and at 1h (i.e. at the end of the infusion). The timing of the collections may be adjusted on the basis of emerging PK or PD data from this study or other new information in order to ensure optimal evaluation of the PD endpoints.

Further details on sample preparation, storage and analysis can be found in the SPM.

6.8. Clinical function assessments

6.8.1. Mixed Meal test (MMT)

A mixed meal tolerance test will be used to determine the level of glucose, insulin, and C-peptide in the blood in response to a specific amount of standard meal consumed. This test involves eating a standard meal, and obtaining multiple blood samples before and at 15, 30, 60, 90, 120, 180, 240 minutes after eating the meal.

On the basis of emerging data from this study or other new information additional analytes e.g. glucagon may be evaluated following the MMT.

Further details for the test procedures can be found in the SPM.

6.8.2. BMI and Waist Circumference

BMI and waist circumference will be monitored at screening and during the study. The times for assessing BMI and waist circumference can be found in Section 3.5, Time and Events Table.

6.9. Pharmacogenetics

Information regarding pharmacogenetic (PGx) research is included in Appendix 3.Appendix 2 The IRB/IEC and, where required, the applicable regulatory agency must approve the PGx assessments before these can be conducted at the site. In some cases, approval of the PGx assessments can occur after approval is obtained for the rest of the study. If so, then the written approval will clearly indicate approval of the PGx assessments is being deferred and in most cases, the study, except for PGx assessments, can be initiated. When PGx assessments will not be approved, then the approval for the rest of the study will clearly indicate this and therefore, PGx assessments will not be conducted.

7. LIFESTYLE AND/OR DIETARY RESTRICTIONS

7.1. Contraception Requirements

7.1.1. Female Subjects

Female subjects of childbearing potential must not become pregnant and so must be sexually inactive by abstinence or use contraceptive methods with a failure rate of < 1%.

Abstinence

Abstinence from penile-vaginal intercourse must be consistent with the preferred and usual lifestyle of the subject. Periodic abstinence (e.g. calendar, ovulation, symptothermal, post-ovulation methods) and withdrawal are not acceptable methods of contraception.

Contraceptive Methods with a Failure Rate of < 1%

- Oral contraceptive, either combined or progestogen alone
- Injectable progestogen
- Implants of etonogestrel or levonorgestrel
- Estrogenic vaginal ring
- Percutaneous contraceptive patches
- Intrauterine device (IUD) or intrauterine system (IUS) that meets the <1% failure rate as stated in the product label
- Male partner sterilization (vasectomy with documentation of azoospermia) prior to the female subject's entry into the study, and this male is the sole partner for that subject. For this definition, "documented" refers to the outcome of the investigator's/designee's medical examination of the subject or review of the subject's medical history for study eligibility, as obtained via a verbal interview with the subject or from the subject's medical records.
- Male condom combined with a female diaphragm, either with or without a vaginal spermicide (foam, gel, cream or suppository).
- Male condom combined with a vaginal spermicide (foam, gel, cream or suppository).

These allowed methods of contraception are only effective when used consistently, correctly and in accordance with the product label. The investigator is responsible for ensuring subjects understand how to properly use these methods of contraception.

7.1.2. Male Subjects

Male subjects with female partners of child-bearing potential must use one of the following contraceptive methods after the first dose of study treatment and until the final follow-up visit on Day 210:

- Condom plus partner use of a highly effective contraceptive (see list in Section 7.1.1).
- Abstinence, defined as sexual inactivity consistent with the preferred and usual lifestyle of the subject. Periodic abstinence (e.g. calendar, ovulation, symptothermal, post-ovulation methods) and withdrawal are not acceptable methods of contraception.

7.2. Meals and Dietary Restrictions

During the study period (Days 1, 29, 56, and 84), subjects will be required to fast from midnight the night before until receipt of a standard breakfast the following day. During each period while subjects are in-clinic all meals will be standardized in term of quantity and quality. Meals should be as consistent as possible across sites within each country. In addition, the meals will be as similar as possible during in-house dosing days.

Overnight fasting from midnight is also required prior to each scheduled blood sample collection for safety labs and biomarker samples.

Subjects will be required to refrain from participating in any weight loss treatment such as, but not limited to "crash"/starvation diets (e.g. <800ckal/day), ALLI (Orlistat), Ephedra (ma-huang) or other ephedrine based treatments, Cascara or other herbal treatments which, in the opinion of the investigator and/or GSK Medical Monitor, contraindicates the subject's participation. Subjects must not participate in any of the weight loss treatment outlined above during the 2weeks prior to dosing and for the remaining course of the study. Subjects should continue to practice healthy eating and lifestyle advice consistent with a diagnosis of Type 2 diabetes.

All subjects will be required to eat the standard meals provided by the clinic unit during overnight stay, and no other food will be allowed

7.3. Caffeine, Alcohol, and Tobacco

- During each dosing session, subjects will abstain from ingesting caffeine- or xanthine-containing products (e.g. coffee, tea, cola drinks, chocolate) for 24 hours prior to the start of dosing until collection of the final pharmacokinetic and or pharmacodynamic sample during each session. Subjects are also required to refrain from caffeine consumption during the 6 hours prior to any ECG assessments.
- During each dosing session, subjects will abstain from alcohol for 24 hours prior to the start of dosing until discharge from the clinic unit. Subjects must also refrain from alcohol for 24 hours prior to all outpatient visits. No more than 3 units of alcohol on average per day, and no more than 21 units on average per week should be consumed throughout the remainder of the study until after the final follow-up visit.
- Subjects who use tobacco products will be instructed that use of nicotine-containing products (including nicotine patches) will not be permitted while they are in the Clinical Unit. Subjects must not smoke more than 10 cigarettes per day during the course of the study.
- Subjects must refrain from all recreational drugs throughout the study (from screening to final follow-up). Drug testing will be performed at the times detailed in Section 3.5 Time and Events Table

7.4. Activity

Subjects will abstain from strenuous exercise for approximately 48 hours prior to screening visit, each study day where safety, PK/PD and biomarker samples will be taken, and follow up visits. Subjects may participate in light recreational activities during studies (e.g., watch television, read*)*

7.5. Travel

Subjects will also be advised to refrain from travelling to countries where there is a high incidence of infectious diseases until after their completion of the study. (e.g. until the follow-up visit on day 210 follow up).

8. CONCOMITANT MEDICATIONS AND NON-DRUG THERAPIES

8.1. Permitted Medications

Metformin monotherapy is allowed for the T2DM subjects during the study. Subjects may be entered if they have stable hypertension or dyslipidemia on therapy, provided there is no change in either the type or dose of medications during the 3-month period before the study.

Paracetamol, at doses of ≤2grams/day is permitted for use during the study. NSAIDs are also permitted for intermittent use during the study.. Other concomitant medication may be considered on a case by case basis by the GSK Medical Monitor.

8.2. Prohibited Medications

Subjects must abstain from taking prescription or non-prescription drugs (including vitamins and dietary or herbal supplements), within 7 days (or 14 days if the drug is a potential enzyme inducer) or 5 half-lives (whichever is longer) prior to the first dose of study medication until completion of the follow-up visit, unless in the opinion of the Investigator and sponsor the medication will not interfere with the study.

Any concomitant medications taken during the study will be recorded in the Case Report Form (CRF). The minimum requirement is that drug name and the dates of administration are to be recorded. Oral antihypertensives and prescription medications to lower cholesterol are permitted provided the subjects are on a stable dose of the same drug for 3 months before Screening. Although stable doses of all concomitant medications are preferable, changes in medications during the study to appropriately treat clinical conditions that might arise, including worsening blood pressure control and dyslipidemia, are allowed.

Anti-inflammatory drugs including corticosteroids, chronic maintenance therapy with NSAIDs, anti-Tumor Necrosis Factor (anti-TNF) or anti-Inerleukin-1 (anti-IL1) are prohibited during the study.

8.3. Non-Drug Therapies

Subjects must abstain from taking any vitamins, herbal and dietary supplements within 7 days or 5 half-lives (whichever is longer) prior to the first dose of study medication until completion of the follow-up visit, unless in the opinion of the Investigator and sponsor the medication will not interfere with the study.

9. COMPLETION OR EARLY WITHDRAWAL OF SUBJECTS

9.1. Subject Completion

A completed subject is one who has completed all phases of the study including the follow-up visit.

The end of the study is defined as the last subject's last visit.

9.2. Subject Withdrawal Criteria

A subject may withdraw from study treatment at any time at his/her own request, or may be withdrawn at any time at the discretion of the investigator for safety, behavioral or administrative reasons.

Subjects may be withdrawn if they fall under any of the following criteria:

1. A subject reaches one of the stopping criteria defined in Section 3.4.
2. A subject experiences unacceptable AE related to study drug or study procedure.
3. A subject demonstrates clinically significant changes in laboratory parameters or other safety monitoring.

9.3. Subject Withdrawal Procedures

9.3.1. Subject Withdrawal from Study

A subject may voluntarily discontinue participation at any time and is not obliged to state a reason for withdrawal. However, the reasons for withdrawal, or failure to provide a reason, must be documented in the CRF by the site staff. The Investigator may also, at his/her discretion; withdraw a subject from this study at any time.

Where possible, if a subject decides to withdraw or is withdrawn through a study day, all scheduled assessments will be taken as planned. The investigator must also make every effort to perform the following evaluations if not already scheduled for that day:

1. Physical exam
2. 12-lead ECG
3. Supine vital signs
4. Clinical laboratory tests (clinical chemistry, hematology and urinalysis)
5. Adverse events assessment

If a subject prematurely withdraws or is withdrawn on a non-study visit day, then the investigator must make every attempt to conduct the above evaluations.

These data should be recorded in the CRF, as they comprise an essential evaluation that should be performed before discharging any subject from the study.

Subjects who withdraw due to AEs will not be replaced and the AE must be followed as indicated in the SPM. Subjects who withdraw for other reasons may be replaced following discussions between the Investigator and study team. Replacement subjects will be assigned to the same treatment as that of the subject they are replacing.

9.3.2. Subject Withdrawal from Study Treatment

A subject will be considered to have prematurely withdrawn from the study drug if he does not receive a complete infusion of study drug for any reason.

Once a subject has discontinued study drug, the subject may not re-enter the study. Dosing of the subjects with investigational product may be stopped at any time, at the request of the subject, or at the discretion of the Principal Investigator (i.e. if clinically significant adverse events should occur). Withdrawal due to AE will be distinguished from withdrawal for other reasons. The reasons for withdrawal and the results of any relevant tests will be recorded in the CRF and the planned follow-up procedures will be performed, where possible.

9.4. Treatment After the End of the Study

Subjects will not receive any additional treatment from GSK after completion of the study because the indication being studied is not life threatening or seriously debilitating and/or other treatment options are available.

The investigator is responsible for ensuring that consideration has been given to the post-study care of the patient's medical condition, whether or not GSK is providing specific post-study treatment.

10. STUDY TREATMENT

Study treatment dosage and administration details are listed in Section 3.4.

10.1. Blinding

This will be a single-blind study. The subjects, site staff and site monitors will be blinded but GSK staff will be unblinded. For details of blinding see Section 6.

10.2. Packaging and Labeling

The contents of the label for GSK1070806 will be in accordance with all applicable regulatory requirements.

10.3. Preparation/Handling/Storage/Accountability

A description of the methods and materials required for preparation of GSK1070806 are provided in the SPM.

Study treatment must be dispensed or administered according to procedures described herein. Only subjects enrolled in the study may receive study treatment. Only authorized

site staff (investigator, study coordinater) may supply or administer study treatment. All study treatment must be stored in a secure area with access limited to the investigator and authorized site staff. Study treatment is to be stored at 2-8°C and protected from light. Maintenance of a temperature log is required.

The investigator, institution, or the head of the medical institution (where applicable) is responsible for study treatment accountability, reconciliation, and record maintenance. The investigator or the head of the medical institution (where applicable), or designated site staff (e.g., storage manager, where applicable) must maintain study treatment accountability records throughout the course of the study. The responsible person(s) will document the amount of investigational product received from and returned to GSK and the amount administered to subjects. The required accountability unit for this study will be number of vital. Discrepancies are to be reconciled or resolved. Procedures for final disposition of unused study treatment are listed in the SPM.

Investigational product is not expected to pose significant occupational safety risk to site staff under normal conditions of use and administration. A Material Safety Data Sheet (MSDS)/equivalent document describing occupational hazards and recommended handling precautions either will be provided to the investigator, where this is required by local laws, or is available upon request from GSK.

However, precautions are to be taken to avoid direct skin contact, eye contact, and generating aerosols or mists. In the case of unintentional occupational exposure notify the monitor, medical monitor and/or study manager.

Precaution will be taken to avoid direct contact with the investigational product. A MSDS describing occupational hazards and recommended handling precautions will be provided to the investigator.

10.4. Assessment of Compliance

When the individual dose for a subject is prepared from a bulk supply, the preparation of the dose will be confirmed by a second member of the study site staff.

When subjects are dosed at the study site, they will receive GSK1070806 or placebo directly from the investigator or designee, under medical supervision. The date and time of each dose administered in the clinic unit will be recorded in the source documents.

A subject may be withdrawn from the study if they do not receive the complete infusion of GSK1070806 or placebo.

10.5. Treatment of Investigational Product Overdose

For this study, any dose of GSK1070806 greater than that planned for the subject will be considered an overdose. For this study, it will be over 5mg/kg per single administration on these dosing days.

GSK does not recommend specific treatment for an overdose. The investigator will use clinical judgment to treat any overdose.

11. ADVERSE EVENTS (AE) AND SERIOUS ADVERSE EVENTS (SAE)

The investigator or site staff is responsible for detecting, documenting and reporting events that meet the definition of an AE or SAE.

AEs will be collected from the time of screening until the final follow-up contact. Medical occurrences that begin prior to the start of investigational product but after obtaining informed consent may be recorded on the Medical History/Current Medical Conditions CRF.

SAEs will be collected over the same time period as stated above for AEs. However, any SAEs assessed as related to study participation (e.g. study treatment, protocol-mandated procedures, invasive tests, or change in existing therapy) or related to a GSK product will be recorded from the time a subject consents to participate in the study up to and including any follow-up contact. All SAEs will be recorded and reported to GSK within 24 hours, as indicated in Section 11.8.

Investigators are not obligated to actively seek AEs or SAEs in former study participants. However, if the investigator learns of any SAE, including a death, at any time after a subject has been discharged from the study, and he/she considers the event reasonably related to the study treatment or study participation, the investigator would promptly notify GSK.

11.1. Definition of Adverse Events

An AE is any untoward medical occurrence in a patient or clinical investigation subject, temporally associated with the use of a medicinal product, whether or not considered related to the medicinal product.

Note: An AE can therefore be any unfavorable and unintended sign (including an abnormal laboratory finding), symptom, or disease (new or exacerbated) temporally associated with the use of a medicinal product.

Events meeting the definition of an AE **include**:

- Any abnormal laboratory test results (hematology, clinical chemistry, or urinalysis) or other safety assessments (e.g., ECGs, radiological scans, vital signs measurements), including those that worsen from baseline, and felt to be clinically significant in the medical and scientific judgement of the investigator.
- Exacerbation of a chronic or intermittent pre-existing condition including either an increase in frequency and/or intensity of the condition.
- New conditions detected or diagnosed after study treatment administration even though it may have been present prior to the start of the study.
- Signs, symptoms, or the clinical sequelae of a suspected interaction.

- Signs, symptoms, or the clinical sequelae of a suspected overdose of either study treatment or a concomitant medication (overdose per se will not be reported as an AE/SAE unless this is an intentional overdose taken with possible suicidal/self-harming intent. This should be reported regardless of sequelae.).
- "Lack of efficacy" or "failure of expected pharmacological action" per se will not be reported as an AE or SAE. However, the signs and symptoms and/or clinical sequelae resulting from lack of efficacy will be reported if they fulfil the definition of an AE or SAE.

Events that **do not** meet the definition of an AE include:

- Any clinically significant abnormal laboratory findings or other abnormal safety assessments that are associated with the underlying disease, unless judged by the investigator to be more severe than expected for the subject's condition.
- The disease/disorder being studied or expected progression, signs, or symptoms of the disease/disorder being studied, unless more severe than expected for the subject's condition.
- Medical or surgical procedure (e.g., endoscopy, appendectomy); the condition that leads to the procedure is an AE.
- Situations where an untoward medical occurrence did not occur (social and/or convenience admission to a hospital).
- Anticipated day-to-day fluctuations of pre-existing disease(s) or condition(s) present or detected at the start of the study that do not worsen.

11.2. Adverse Events of Special Interest

11.2.1. Home Blood Glucose Monitoring

Although it is not anticipated that administration of GSK1070806 will cause hyperglycaemia subjects will be instructed in the use of home blood glucose monitoring to monitor unexpected changes in blood glucose levels. The subjects should monitor their blood glucose as per the instructions of the investigator and as appropriate for their medical management (e.g., several times per week). The subjects should report promptly, as directed by the investigator, the occurrence of hyperglycemia or hypoglycemia (particularly if symptomatic) to the investigator (or his or her designee). Assessment and action should then occur as deemed appropriate by the investigator.

11.2.2. Hypoglycemia Events

Although it is not anticipated that administration of GSK1070806 will cause hypoglycaemia specific criteria for monitoring hypoglycemia events have been designed to ensure subject safety and to closely monitor hypoglycemia. Subjects will be instructed at each visit through the end-of-treatment visit on diet, exercise, and home blood glucose monitoring as well as on the signs and symptoms of hypoglycemia and on supplemental oral glucose treatment, if needed. In addition, subjects will be instructed to test their blood glucose any time they experience signs and symptoms of hypoglycemia to confirm that it is a hypoglycemia episode, defined in Appendix 4. It is not anticipated that administration of GSK1070806 will cause hyperglycaemia.

11.2.3. Allergic Reactions

Although it is considered unlikely for acute allergic reactions to occur in response to GSK1070806 exposure, all subjects will be monitored carefully for evidence of allergic response. It is important to recognize early signs of an anaphylactoid reaction and prevent progression to severe anaphylaxis. If such symptoms are developing, the subjects will be managed according to the guidelines from the Resuscitation Council (UK) on Emergency Treatment of Anaphylactic Reactions. Staff in the Clinic unit, where the study will be conducted, are trained according to the council's algorithms (physicians are trained in Advanced Life Support; nursing and technician staff are trained in Immediate or Basic Life Support). Any subject requiring treatment for suspected anaphylaxis will be transferred to an acute medical unit for further observation as judged necessary by the medical team in charge of the clinical emergency situation. Subjects will be advised to seek immediate medical attention if they develop a generalised rash, itching, severe nausea, swelling of the lips or tongue or difficulty in breathing after leaving the unit. Any subject that is treated for suspected anaphylaxis will be withdrawn from the study.

11.2.4. Infections

Given the possible impact of GSK1070806 on immunity to infection, all subjects will be rigorously screened by clinical exam for ongoing or potentially emergent infections by a physician at the screening visit, prior to administration of the first and second doseson Days 1 and 29 , and during the course of the study. Furthermore, intensive monitoring for clinical signs of infection (included body temperature) will be carried out at each visit. In addition, subjects who develop clinical signs of an infection during their involvement in the study will be clinically reviewed and samples taken for virologic and/or microbiologic confirmation of the infection as appropriate and in accordance with local guidelines. Plasmapharesis may be employed in the event of a serious adverse event or infection. During the out-patient follow-up period, subjects will be instructed to contact the clinical unit if they develop any signs or symptoms related to an infection.

11.2.5. Influenza Management

If any subject develops signs and symptoms related to influenza (for example body temperature fever (>38°C) or a history of fever, and two or more of the following symptoms: cough, sore throat, runny nose, sneezing, limb / joint pain, headache, vomiting / diarrhoea in the absence of a known cause) during participation in the study, the subject will be treated as per local protocols and tested for Influenza A and B (by RT-PCR) including H1N1. Subjects will be offered anti-viral therapy where possible (e.g Tamiflu or RELENZA). Each case of infection will be carefully reviewed and assessed for potential relatedness to study drug after consultation between the Sponsor's Medical Monitor, the study Investigator and appropriate specialists. Additionally, if a household member of a subject has the flu, and the subject has not been vaccinated for the flu, the subject should be offered anti-viral therapy where possible (e.g Tamiflu or RELENZA).

11.3. Definition of Serious Adverse Events

If an event is not an AE per Section 11.1, then it cannot be an SAE even if serious conditions are met (e.g., hospitalization for signs/symptoms of the disease under study, death due to progression of disease, etc).

An SAE is any untoward medical occurrence that, at any dose:

a. Results in death

b. Is life-threatening

   NOTE: The term 'life-threatening' in the definition of 'serious' refers to an event in which the subject was at risk of death at the time of the event. It does not refer to an event, which hypothetically might have caused death, if it were more severe.

c. Requires hospitalization or prolongation of existing hospitalization

   NOTE: In general, hospitalization signifies that the subject has been detained (usually involving at least an overnight stay) at the hospital or emergency ward for observation and/or treatment that would not have been appropriate in the physician's office or out-patient setting. Complications that occur during hospitalization are AEs. If a complication prolongs hospitalization or fulfills any other serious criteria, the event is serious. When in doubt as to whether "hospitalization" occurred or was necessary, the AE should be considered serious.

   Hospitalization for elective treatment of a pre-existing condition that did not worsen from baseline is not considered an AE.

d. Results in disability/incapacity, or

   NOTE: The term disability means a substantial disruption of a person's ability to conduct normal life functions. This definition is not intended to include experiences of relatively minor medical significance such as uncomplicated headache, nausea, vomiting, diarrhea, influenza, and accidental trauma (e.g. sprained ankle) which may interfere or prevent everyday life functions but do not constitute a substantial disruption.

e. Is a congenital anomaly/birth defect

f. Medical or scientific judgment should be exercised in deciding whether reporting is appropriate in other situations, such as important medical events that may not be immediately life-threatening or result in death or hospitalization but may jeopardize the subject or may require medical or surgical intervention to prevent one of the other outcomes listed in the above definition. These should also be considered serious. Examples of such events are invasive or malignant cancers, intensive treatment in an emergency room or at home for allergic bronchospasm, blood dyscrasias or convulsions that do not result in hospitalization, or development of drug dependency or drug abuse.

g. Is associated with liver injury **and** impaired liver function defined as:

   - ALT ≥ 3xULN and total bilirubin[*] ≥ 2xULN (>35% direct), **or**
   - ALT ≥ 3xULN and INR[**] > 1.5.

*Serum bilirubin fractionation should be performed if testing is available; if unavailable, measure urinary bilirubin via dipstick. If fractionation is unavailable and ALT ≥ 3xULN and total bilirubin ≥ 2xULN, then the event is still to be reported as an SAE.

- ** INR testing not required per protocol and the threshold value does not apply to subjects receiving anticoagulants. If INR measurement is obtained, the value is to be recorded on the SAE form.
- Refer to Section 12 for the required liver chemistry follow-up instructions

11.4. Method of Detecting AEs and SAEs

Care will be taken not to introduce bias when detecting AEs and/or SAEs. Open-ended and non-leading verbal questioning of the subject is the preferred method to inquire about AE occurrence. Appropriate questions include:

- “How are you feeling?”
- “Have you had any (other) medical problems since your last visit/contact?”
- “Have you taken any new medicines, other than those provided in this study, since your last visit/contact?”

11.5. Recording of AEs and SAEs

When an AE/SAE occurs, it is the responsibility of the investigator to review all documentation (e.g., hospital progress notes, laboratory, and diagnostics reports) relative to the event. The investigator will then record all relevant information regarding an AE/SAE in the appropriate data collection tool.

It is not acceptable for the investigator to send photocopies of the subject’s medical records to GSK in lieu of completion of the GSK, AE/SAE data collection tool. However, there may be instances when copies of medical records for certain cases are requested by GSK. In this instance, all subject identifiers, with the exception of the subject number, will be blinded on the copies of the medical records prior to submission of to GSK.

The investigator will attempt to establish a diagnosis of the event based on signs, symptoms, and/or other clinical information. In such cases, the diagnosis will be documented as the AE/SAE and not the individual signs/symptoms.

11.6. Evaluating AEs and SAEs

11.6.1. Assessment of Intensity

The investigator will make an assessment of intensity for each AE and SAE reported during the study and will assign it to one of the following categories:

Mild: An event that is easily tolerated by the subject, causing minimal discomfort and not interfering with everyday activities.

Moderate: An event that is sufficiently discomforting to interfere with normal everyday activities.

Severe: An event that prevents normal everyday activities.

An AE that is assessed as severe will not be confused with an SAE. Severity is a category utilized for rating the intensity of an event; and both AEs and SAEs can be assessed as severe. An event is defined as 'serious' when it meets at least one of the pre-defined outcomes as described in the definition of an SAE.

11.6.2. Assessment of Causality

The investigator is obligated to assess the relationship between study treatment and the occurrence of each AE/SAE. A "reasonable possibility" is meant to convey that there are facts/evidence or arguments to suggest a causal relationship, rather than a relationship cannot be ruled out. The investigator will use clinical judgment to determine the relationship. Alternative causes, such as natural history of the underlying diseases, concomitant therapy, other risk factors, and the temporal relationship of the event to the study treatment will be considered and investigated. The investigator will also consult the Investigator Brochure (IB) and/or Product Information, for marketed products, in the determination of his/her assessment.

For each AE/SAE the investigator must document in the medical notes that he/she has reviewed the AE/SAE and has provided an assessment of causality.

11.7. Follow-up of AEs and SAEs

After the initial AE/SAE report, the investigator is required to proactively follow each subject at subsequent visits/contacts. All AEs and SAEs will be followed until resolution, until the condition stabilizes, until the event is otherwise explained, or until the subject is lost to follow-up.

The investigator is obligated to perform or arrange for the conduct of supplemental measurements and/or evaluations as may be indicated or as requested by GSK to elucidate as fully as possible the nature and/or causality of the AE or SAE. The investigator is obligated to assist. This may include additional laboratory tests or investigations, histopathological examinations or consultation with other health care professionals. If a subject dies during participation in the study or during a recognized follow-up period, the investigator will provide GSK with a copy of any post-mortem findings, including histopathology.

New or updated information will be recorded in the originally completed data collection tool. The investigator will submit any updated SAE data to GSK within the designated reporting time frames.

11.8. Prompt Reporting of SAEs to GSK

Once the investigator determines that an event meets the protocol definition of an SAE, the SAE will be reported to GSK **within 24 hours**. Any follow-up information on a previously reported SAE will also be reported to GSK within 24 hours.

If the investigator does not have all information regarding an SAE, he/she will not wait to receive additional information before notifying GSK of the event and completing the appropriate data collection tool. The investigator will always provide an assessment of causality at the time of the initial report as described in Section 11.6.2, Assessment of Causality.

The primary mechanism for reporting SAEs to GSK will be the electronic data collection tool. For this study is InForm. If the electronic system is unavailable for greater than 24 hours, the site will use the paper SAE data collection tool and fax it to the GSK Medical Monitor, protocol contact (Clinical Study Manager) and date manager. Then the site will enter the serious adverse event data into the electronic system as soon as it becomes available.

After the study is completed at a given site, the electronic data collection tool (e.g., InForm system) will be taken off-line to prevent the entry of new data or changes to existing data. If a site receives a report of a new SAE from a study participant or receives updated data on a previously reported SAE after the electronic data collection tool has been taken off-line, the site can report this information on a paper SAE form or to their GSK protocol contact by telephone.

GSK contacts for SAE receipt can be found at the beginning of this protocol on the Sponsor/Medical Monitor Contact Information page.

11.9. Regulatory Reporting Requirements For SAEs

Prompt notification of SAEs by the investigator to GSK is essential so that legal obligations and ethical responsibilities towards the safety of subjects are met.

GSK has a legal responsibility to notify both the local regulatory authority and other regulatory agencies about the safety of a product under clinical investigation. GSK will comply with country specific regulatory requirements relating to safety reporting to regulatory authorities, IRBs/IECs and investigators.

Investigator safety reports are prepared for suspected unexpected serious adverse reactions according to local regulatory requirements and GSK policy and are forwarded to investigators as necessary. An investigator who receives an investigator safety report describing an SAE(s) or other specific safety information (e.g., summary or listing of SAEs) from GSK will file it with the IB and will notify the IRB/IEC, if appropriate according to local requirements.

12. LIVER CHEMISTRY FOLLOW-UP PROCEDURES

Refer to the diagram in Appendix 1 for a visual presentation of the procedures listed below.

The procedures listed below are to be followed if a subject meets any of the liver chemistry stopping criteria defined in Section 3.4.1.1:

- Immediately and permanently withdraw the subject from study treatment
- Notify the GSK medical monitor within 24 hours of learning of the abnormality to confirm the subject's study treatment cessation and follow-up.
- Complete the "Safety Follow-Up Procedures" listed below.
- Complete the liver event case report forms. If the event also meets the criteria of an SAE (see Section 11.3), the SAE data collection tool will be completed separately with the relevant details.
- Upon completion of the safety follow-up withdraw the subject from the study unless further safety follow up is required or GSK Medical Governance approval of drug restart is granted (see Section 12.1).
- Do not restart investigational product unless written approval is granted by GSK Medical Governance (see Section 12.1), whereupon the subject continues in the study after completion of the liver chemistry monitoring.

Safety Follow-Up Procedures for subjects with ALT ≥ 3xULN and bilirubin ≥ 2xULN (>35% direct); or ALT ≥ 3xULN and INR[1] > 1.5 [Stopping Criteria #1]:

- Make every reasonable attempt to have the subject return to the clinic within 24-72 hrs for repeat liver chemistries and additional testing.
- Monitor subjects <u>weekly</u> until liver chemistries (ALT, AST, alkaline phosphatase, bilirubin) resolve, stabilize or return to within baseline values.

Safety Follow-Up Procedures for subjects with ALT ≥ 5xULN or ALT ≥ 3xULN who have hepatitis symptoms or rash, can't be monitored for 4 weeks or have elevations that persist ≥ 4 weeks [Stopping Criteria #2 - #5]:

- Make every reasonable attempt to have the subject return to the clinic within 24-72 hrs for repeat liver chemistries and additional testing.
- Monitor subjects <u>weekly</u> until liver chemistries (ALT, AST, alkaline phosphatase, bilirubin) resolve, stabilize or return to within baseline values.

[1] INR testing not required per protocol and the threshold value does not apply to subjects receiving anticoagulants.

Safety Follow-Up Procedures for subjects with ALT ≥ 3xULN and < 5xULN and bilirubin < 2xULN, who do not exhibit hepatitis symptoms or rash:

- Notify the GSK medical monitor within 24 hours of learning of the abnormality to discuss subject safety.
- Subject can continue study treatment if liver chemistries (ALT, AST, alkaline phosphatase, bilirubin) can be monitored weekly for up to 4 weeks.
- If at any point these subjects meet the liver chemistry stopping criteria (outlined in Section 3.4.1.1), immediately withdraw study treatment, perform additional testing and continue safety follow-up until liver chemistries resolve, stabilize or return to baseline values.
- After 4 weeks of monitoring, if ALT < 3xULN and bilirubin < 2xULN, subjects must be monitored twice monthly until liver chemistries normalize or return to within baseline values.

Additional Follow-Up Procedures for subjects who meet *any* of the stopping criteria:

- Viral hepatitis serology including:
  - Hepatitis A IgM antibody;
  - Hepatitis B surface antigen and Hepatitis B Core Antibody (IgM);
  - Hepatitis C RNA;
  - Cytomegalovirus IgM antibody;
  - Epstein-Barr viral capsid antigen IgM antibody (or if unavailable, obtain heterophile antibody or monospot testing);
  - Hepatitis E IgM antibody.
- Blood sample for PK analysis. Record the date/time of the PK blood sample draw and the date/time of the last dose of study treatment prior to blood sample draw on the CRF. If the date or time of the last dose is unclear, provide the subject's best approximation. If the date/time of the last dose can not be approximated OR a PK sample can not be collected within a week of the liver event **do not obtain a PK sample**. Instructions for sample handling and shipping are included in the SPM.
- Serum creatine phosphokinase (CPK) and lactate dehydrogenase (LDH).
- Fractionate bilirubin, if total bilirubin ≥ 2xULN.
- Assess eosinophilia
- Record the appearance or worsening of clinical symptoms of hepatitis (fatigue, nausea, vomiting, right upper quadrant pain or tenderness, fever, rash or eosinophilia) as relevant on the AE CRF
- Record use of concomitant medications, acetaminophen, herbal remedies, other over the counter medications, or putative hepatotoxins on the Concomitant Medications CRF.

- Record alcohol use on the Liver Events CRF.

The following are required for subjects with ALT ≥ 3xULN **and** bilirubin ≥ 2xULN (>35% direct) but are optional for other abnormal liver chemistries:

- Anti-nuclear antibody, anti-smooth muscle antibody, and Type 1 anti-liver kidney microsomal antibodies and quantitative total immunoglobulin G (IgG or gamma globulins).
- Serum acetaminophen adduct assay (quantifies potential acetaminophen contribution to liver injury, detectable by High-Performance Liquid Chromatograph (HPLC) assay more than 1 week following acetaminophen use [James, 2009]).
- Liver imaging (ultrasound, magnetic resonance, or computerized tomography) to evaluate liver disease.
- The Liver Imaging and/or Liver Biopsy CRFs are also to be completed if these tests are performed.

12.1. Restarting Investigational Product

Following a Liver Event that is possibly related to investigational product, GSK will consider drug rechallenge where:

- the subject is receiving compelling benefit,
- the benefit of drug restart exceeds risk, and
- no effective alternative therapy is available

for the drug restart/rechallenge process

13. STUDY CONDUCT CONSIDERATIONS

13.1. Posting of Information on Publicly Available Clinical Trial Registers

Study information from this protocol will be posted on publicly available clinical trial registers before enrollment of subjects begins.

13.2. Regulatory and Ethical Considerations, Including the Informed Consent Process

GSK will obtain favorable opinion/approval to conduct the study from the appropriate regulatory agency in accordance with any applicable country-specific regulatory requirements prior to a site initiating the study in that country.

The study will be conducted in accordance with all applicable regulatory requirements.

The study will also be conducted in accordance with ICH Good Clinical Practice (GCP), all applicable subject privacy requirements, and, the guiding principles of the 2008 Declaration of Helsinki. This includes, but is not limited to, the following:

- IRB/IEC review and favorable opinion/approval to conduct the study and of any subsequent relevant amended documents
- Written informed consent (and any amendments) to be obtained for each subject before participation in the study
- Investigator reporting requirements (e.g. reporting of AEs/SAEs/protocol deviations to IRB/IEC)

Written informed consent must be obtained from each subject prior to participation in the study.

Information regarding pharmacogenetic research is included in Appendix 3. In approving the clinical protocol the IEC/IRB and, where required, the applicable regulatory agency must also approve the PGx assessments (i.e., approval of Appendix 3), unless otherwise indicated. Where permitted by regulatory authorities, approval of the PGx assessments can occur after approval is obtained for the rest of the study. If so, then the written approval will clearly indicate approval of the PGx assessments is being deferred and the study, except for PGx assessments, can be initiated. When PGx assessments are not approved, then the approval for the rest of the study will clearly indicate this and therefore, PGx assessments will not be conducted.

13.2.1. Urgent Safety Measures

If an event occurs that is related to the conduct of the study or the development of the study treatment, and this new event is likely to affect the safety of subjects, the sponsor and the investigator will take appropriate urgent safety measures to protect subjects against any immediate hazard.

The sponsor will work with the investigator to ensure the IEC/IRB is notified within 3 days if the study is based in the UK.

13.3. Quality Control (Study Monitoring)

In accordance with applicable regulations including GCP, and GSK procedures, GSK monitors will contact the site prior to the start of the study to review with the site staff the protocol, study requirements, and their responsibilities to satisfy regulatory, ethical, and GSK requirements. When reviewing data collection procedures, the discussion will also include identification, agreement and documentation of data items for which the InForm will serve as the source document.

GSK will monitor the study and site activity to verify that the:

- Data are authentic, accurate, and complete.
- Safety and rights of subjects are being protected.

- Study is conducted in accordance with the currently approved protocol and any other study agreements, GCP, and all applicable regulatory requirements.

The investigator and the head of the medical institution (where applicable) agrees to allow the monitor direct access to all relevant documents

13.4. Quality Assurance

To ensure compliance with GCP and all applicable regulatory requirements, GSK may conduct a quality assurance assessment and/or audit of the site records, and the regulatory agencies may conduct a regulatory inspection at any time during or after completion of the study. In the event of an assessment, audit or inspection, the investigator (and institution) must agree to grant the advisor(s), auditor(s) and inspector(s) direct access to all relevant documents and to allocate their time and the time of their staff to discuss the conduct of the study, any findings/relevant issues and to implement any corrective and/or preventative actions to address any findings/issues identified.

13.5. Study and Site Closure

Upon completion or premature discontinuation of the study, the monitor will conduct site closure activities with the investigator or site staff, as appropriate, in accordance with applicable regulations including GCP, and GSK procedures.

In addition, GSK reserves the right to temporarily suspend or prematurely discontinue this study at any time for reasons including, but not limited to, safety or ethical issues or severe non-compliance. For multicenter studies, this can occur at one or more or at all sites. If GSK determines such action is needed, GSK will discuss this with the investigator or the head of the medical institution (where applicable), including the reasons for taking such action. When feasible, GSK will provide advance notification to the investigator or the head of the medical institution, where applicable, of the impending action prior to it taking effect.

If the study is suspended or prematurely discontinued for safety reasons, GSK will promptly inform investigators or the head of the medical institution (where applicable) and the regulatory authorities of the suspension or premature discontinuation of the study and the reason(s) for the action. If required by applicable regulations, the investigator or the head of the medical institution (where applicable) must inform the IRB/IEC promptly and provide the reason for the suspension or premature discontinuation.

13.6. Records Retention

Following closure of the study, the investigator or the head of the medical institution (where applicable) must maintain all site study records, except for those required by local regulations to be maintained by someone else, in a safe and secure location. The records must be maintained to allow easy and timely retrieval, when needed (e.g., audit or inspection), and, whenever feasible, to allow any subsequent review of data in conjunction with assessment of the facility, supporting systems, and staff. Where permitted by local laws/regulations or institutional policy, some or all of these records can be maintained in a format other than hard copy (e.g., microfiche, scanned,

electronic); however, caution needs to be exercised before such action is taken. The investigator must assure that all reproductions are legible and are a true and accurate copy of the original, and meet accessibility and retrieval standards, including re-generating a hard copy, if required. Furthermore, the investigator must ensure there is an acceptable back-up of these reproductions and that an acceptable quality control process exists for making these reproductions.

GSK will inform the investigator of the time period for retaining these records to comply with all applicable regulatory requirements. The minimum retention time will meet the strictest standard applicable to that site for the study, as dictated by any institutional requirements or local laws or regulations, or GSK standards/procedures; otherwise, the retention period will default to 15 years.

The investigator must notify GSK of any changes in the archival arrangements, including, but not limited to, archival at an off-site facility or transfer of ownership of the records in the event the investigator leaves the site.

13.7. Provision of Study Results to Investigators, Posting of Information on Publically Available Clinical Trials Registers and Publication

Where required by applicable regulatory requirements, an investigator signatory will be identified for the approval of the clinical study report. Investigators and authors will be provided reasonable access to statistical tables, figures, and relevant reports and will have the opportunity to review the complete study results at a GSK site or other mutually-agreeable location.

GSK will also provide investigators with the full summary of the study results. Investigators are encouraged to share the summary results with the study subjects, as appropriate.

GSK will provide investigator with the randomization codes for their site after completion of the full statistical analysis.

GSK aims to post a results summary to the GSK Clinical Study Register and other publicly available registers no later than 8 months after the last subject's last visit (LSLV) [this applies to each data analysis phase for studies with multiple phases, e.g., primary analysis, follow up analysis etc]. In addition, the aim is to submit a manuscript to a peer-reviewed journal for publication within 18 months of LSLV. GSK also aims to publish the full study protocol on the GSK Clinical Study Register at the time the results of the study are published as a manuscript in the scientific literature.

When manuscript publication in a peer-reviewed journal is not feasible, further study information will be posted to the GSK Clinical Study Register to supplement the results summary.

A manuscript will be progressed for publication in the scientific literature if the results provide important scientific or medical knowledge.

13.8. Data Management

For this study subject data will be entered into GSK defined electronic case report forms (eCRFs, InForm), transmitted electronically to GSK and combined with data provided from other sources in a validated data system.

Management of clinical data will be performed in accordance with applicable GSK standards and data cleaning procedures to ensure the integrity of the data, e.g., removing errors and inconsistencies in the data. Adverse events and concomitant medications terms will be coded using Medical Dictionary for Regulatory Activities (MedDRA). eCRFs (including queries and audit trails) will be retained by GSK, and copies will be sent to the investigator to maintain as the investigator copy. Subject initials will not be collected or transmitted to GSK according to GSK policy.

For this study subject data will be collected using GSK defined case report forms, InForm and combined with data provided from other sources in a validated data system.

14. REFERENCES

American Diabetes Association Workgroup on Hypoglycemia (2005). Defining and Reporting Hypoglycemia in Diabetes: A report from the American Diabetes Association Workgroup on Hypoglycemia**.** Diabetes Care; **28**:1245-124.

Amin MA, Mansfield PJ, Pakozdi A, Campbell PL, Ahmed S, Martinez RJ, et al.(2007) Interleukin-18 induces angiogenic factors in rheumatoid arthritis synovial tissue fibroblasts via distinct signaling pathways. Arthritis Rheum; **56**:1787–97.

Araki S et al (2007).Predictive impact of elevated serum level of IL-18 for early renal dysfunction in type 2 diabetes: an observational follow-up study. Diabetologia, **50**:867–873.

Blakenberg S., et al. (2003). Interleukin-18 and the Risk of Coronary Heart Disease in European Men: The Prospective Epidemiological Study of Myocardial Infarction (PRIME). Circulation. **108**:2453-2459.Bosch, 2005a;

Bosch, M et al. (2005a). Potential Role of Interleukin-18 in Liver Disease Associated with Insulin Resistance. Obes Res.**13**:1925–1931.

Bosch, M., et al. (2005b). Circulating IL-18 concentration is associated with insulin sensitivity and glucose tolerance through increased fat-free mass. Diabetologia. **48**: 1841–1843.

Bruun, J., Stallknecht, B. (2007). Interleukin-18 in plasma and adipose tissue: effects of obesity, insulin resistance, and weight loss. Eur. J. Endocrin. **157**: 465–471.

Chandrasekar B, Mummidi S, Claycomb WC, Mestril R, Nemer M (2005). Interleukin-18 is a pro-hypertrophic cytokine that acts through a phosphatidylinositol 3-kinase-phosphoinositide-dependent kinase-1-Akt-GATA4 signaling pathway in cardiomyocytes. J Biol Chem, **280**:4553–67.

Donath M, Whitmore J, Bauer R, Scannon P,Weder C, et al. (2008). Xoma 052, an anti-IL-1b antibody, in a double-blind, placebo-controlled, dose-escalation study of the safety and pharmacokinetics in patients with type 2 diabetes mellitus, a new approach to therapy. Diabetologia **51**:[Suppl 1; S7(1)]

DuckworthW, Abraira C, Moritz T, et al (2009).. Glucose control and vascular complications in Veterans with type 2 diabetes. N Engl J Med. **360**:129–139.

Escobar-Morreale H.F., Botella-Carretero J.I., Villuendas G., Sancho J., San Millán, J.L. (2004). Serum Interleukin-18 Concentrations Are Increased in the Polycystic Ovary Syndrome: Relationship to Insulin Resistance and to Obesity. Journal of Clinical Endocrinology and Metabolism, **89**: 806-811.

Espinola-Klein, C. et al.(2008) Impact of inflammatory markers on cardiovascular mortality in patients with metabolic syndrome. European Journal of Cardiovascular Prevention and Rehabilitation.**15**:278–284

Esposito K, Pontillo A, Ciotola M et al. (2002) Weight loss reduces interleukin-18 levels in obese women. J Clin Endocrinol Metab, **87**:3864–3866.

Fain JN, Tichansky DS, Madan AK (2006). Most of the interleukin 1 receptor antagonist, cathepsin S, macrophage migration inhibitory factor, nerve growth factor, and interleukin 18 release by explants of human adipose tissue is by the non-fat cells, not by the adipocytes. Metabolism, **55**:1113-1121.

Fernandez-Real, JM, Broch, M; Vendrell, J; Gutierrez, C; Casamitjana, R; Pugeat,M; Richart, C; Ricart W (2000). Interleukin-6 gene polymorphism and insulin sensitivity, Diabetes, **49**: 517– 520.

Fischer CP, Perstrup LB, Berntsen A, Eskildsen P, Pedersen BK (2005). Elevated plasma interleukin-18 is a marker of insulin-resistance in type 2 diabetic and non-diabetic humans. Clin Immunol;**117**:152–60.

Fix C, Bingham K and Carver W (2011). Effects of interleukin-18 on cardiac fibroblast function and gene expression Cytokine, **53**:19–28.

Frigerio S, Holländer GA, Zumsteg U. (2002). Functional IL-18 is produced by primary pancreatic mouse islets and NIT-1 beta cells and participates in the progression towards destructive insulitis. Hormone Research; **57:** 94-104.

Fujita T, Ogihara N., Kamura Y., Satomura A., Fuke Y, Shimizu C, Wada Y , Matsumoto K (2010). Interleukin-18 contributes more closely to the progression of diabetic nephropathy than other diabetic complications. Acta Diabetologica, Online Firtst: DOI: 10.1007/s00592-010-0178-4.

Garcia MC, et al. (2006). Mature-onset obesity in interleukin-1 receptor I knockout mice. Diabetes.**55**(5):1205–13.

Gerdes N, Sukhova GK, Libby P, Reynolds RS, Young JL, Schönbeck U (2002). Expression of interleukin (IL)-18 and functional IL-18 receptor on human vascular endothelial cells, smooth muscle cells and macrophages: implications for atherogenesis. J Exp Med,**195**:245–57.

Gerstein HC, Miller ME, Byington RP, et al, (2008). Action to Control Cardiovascular Risk in Diabetes Study Group. Effects of intensive glucose lowering in type 2 diabetes.N Engl J Med. **358**:2545–2559.

GlaxoSmithKline Document Number 2011N113387/00, Clinical Pharmacology Study Report for GSK1614235 in Healthy Subject. (Version 00). Effective Date: 24th May, 2011.

GlaxoSmithKline Document Number GM2007/00045/09, Protocol Amendment 7 FOR GSK1070806 in Healthy and Obese Subjects. (Version 09). Effective Date: 01 Sep, 2011.

GlaxoSmithKline Document Number RM2006/00553/00, Clinical Pharmacology Study Report for GSK189075 with GW869682 in Subject with T2DM. (Version 00). Effective Date: 02 July, 2007.

GlaxoSmithKline Document Number WM2009/00047/01, GSK1070806 Investigator's Brochure (Version 1). Effective Date: June, 2011.

Goldfine, A. Silver, R. et al (2008). Use of Salsalate to Target Inflammation in the Treatment of Insulin Resistance and Type 2 Diabetes. Clin. Translat. Sci**. 1(1):** 36-43.

Grimble, R. F. (2002). Inflammatory status and insulin resistance. Curr. Opin. Clin. Nutr. Metab. Care **5**: 551–559.

Herder C, Baumert J, Thorand B, Koenig W, de Jager W, Meisinger C, Illig T, Martin S, Kolb H.(2006). Chemokines as risk factors for type 2 diabetes: results from the MONICA/KORA Augsburg study, 1984-2002. Diabetologia. **49**(5):921-9. Epub 2006 Mar 11.

Hulthe J, McPheat W, Samnegård A, Tornvall P, Hamsten A, Eriksson P, (2006). Plasma interleukin (IL)-18 concentrations is elevated in patients with previous myocardial infarction and related to severity of coronary atherosclerosis independently of C-reactive protein and IL-6. Atherosclerosis;**188**:450–4.

Hung, J., McQuillan, B. M. (2005). Elevated IL-18 levels are associated with the metabolic syndrome independent of obesity and insulin resistance. Arterioscler. Thromb. Vasc. Biol. **25**:1-6.`

James LP. (2009). Pharmacokinetics of Acetaminophen - Protein Adducts Liver Failure. Drug Metab Disp, **37**:1779-1784.

Jin Kim, H., Seok Kang, E. et al. (2007). Effects of rosiglitazone and metformin on inflammatory markers and adipokines: decrease in interleukin-18 is an independent factor for the improvement of homeostasis model assessment-beta in type 2 diabetes mellitus. Clin. Endocrin. **66**: 282–289.Larsen CM.

Jung MK, Song HK, Kim KE, Hur DY, Kim T, Bang S, et al (2006). Il-18 enhances the migration ability of murine melanoma cells through the generation of ROI and the MAPK pathway. Immunol Lett,**107**:125–30.

Kaine J. L, Kivitz AJ, Birbara C, Luo AY (2007). Immune responses following administration of influenza and pneumococcal vaccines to patients with rheumatoid arthritis receiving adalimumab. J Rheumatol. **34(2):**272-9.

Khan F, Peltekian KM, Peterson TC, (2008). Effect of interferon-alpha, ribavirin,pentoxifylline and interleukin-18 antibody on hepatitis C sera-stimulated hepatic stellat cell proliferation. J Interferon Cytokine Res,**28**:643–51.

Kim KE, Song H, Kim TS, Yoon D, Kim CW, Bang SI, et al.(2007). Interleukin-18 is a critical factor for vascular endothelial growth factor-enhanced migration in human gastric cancer cell lines. Oncogene;**26**:1468–76.

Larsen CM, Faulenbach M, Vaag A, Vølund A, Ehses JA, Seifert B, Mandrup-Poulsen T, Donath MY, (2007) Interleukin-1-receptor antagonist in type 2 diabetes mellitus. N Engl J Med. **356**(15):1517-26.

Lewis, E.C. & Dinarello, C. (2006) Responses of IL-18- and IL-18 receptor-deficient pancreatic islets with convergence of positive and negative signals for the IL-18 receptor. PNAS. **103**:16852–16857.

Lin, S-Y; Wang,Y; Sheu, H (2004). Increased serum soluble tumor necrosis factor receptor levels are associated with insulin resistance in liver cirrhosis, Metabolism, **53**: 922– 926.

Mallat Z, Corbaz A, Scoazec A, Besnard S, Lesèche G, Chvatchko Y, et al. (2001) Expression of interleukin-18 in human atherosclerotic plaques and relation to plaque instability. Circulation,**104**:1598–603.

Marleaut AM and Sarvetnick N E (2011) IL-18 is required for self-reactive T cell expansion in NOD mice. Journal of Autoimmunity, **36**: 263-277.

Mishima, Y; Kuyama, A; Tada,A; Takahashi, K; Ishioka, K; Kibata, M (2002). Relationship between serum tumor necrosis factor-[alpha] and insulin resistance in obese men with Type 2 diabetes mellitus, Diabetes. Res. Clin. Pract. **52**:119– 123.

Miyauchi K, Takiyama Y, Honjyo J, Tateno M, Haneda M. Upregulated IL-18 expression in type 2 diabetic subjects with nephropathy (2009). TGF-beta1 enhanced IL-18 expression in human renal proximal tubular epithelial cells. Diabetes Res Clin Pract ;**83**:190–199.

Montori VM, Ferna´ ndez-Balsells M. (2009). Glycemic control in type 2 diabetes: time for an evidence-based aboutface? Ann Intern Med. April 20, **150** [Epub ahead of print]

Moriwaki, (2003). Moriwaki Y, Yamamoto T, Shibutani Y, et al. Elevated levels of interleukin-18 and tumor necrosis factor-alpha in serum of patients with type 2 diabetes mellitus: Relationshipwith diabetic nephropathy. Metabolism 2003;**52**:605–608.

Nakamura, (2005). Nakamura A, Shikata K, Hiramatsu M, et al. Serum interleukin-18 levels are associated with nephropathy and atherosclerosis in Japanese patients with type 2 diabetes. Diabetes Care 2005;**28**:2890–2895.

Napolitano, L., Park, PK., Sihler, K. P., Papadimos. T. (2009). Intensive-Care Patients With Severe Novel Influenza A (H1N1) Virus Infection. CDCs MMWR. **58**(Dispatch);1-4.

Natea, M., Joosten, L. (2006). Deficiency of interleukin-18 in mice leads to hyperphagia, obesity and insulin resistance. Nat. Med. **12:** 650-656.

Okamura H, Tsutsui H, Komatsu T et al (1995) Cloning of a new cytokine that induces IFN-gamma production by T cells. Nature, **378**:88–91

Park S, Cheon S, Cho D. (2007). The dual effects of interleukin-18 in tumor progression. Cell Mol Immunol, **4**:329–35.

Pickup, J. C. & Crook, M. A. (1998). Is type II diabetes mellitus a disease of the innate immune system? Diabetologia 41: 1241–1248.

Pickup, J. C., Mattock, M. B., Chusney, G. D. & Burt, D. (1997). NIDDM as a disease of the innate immune system: association of acute-phase reactants and interleukin-6 with metabolic syndrome X. Diabetologia, **40**:1286–1292.

Rabkin, S. (2009). The role of interleukin 18 in the pathogenesis of hypertension-induced vascular disease. Nature Clinical Practice. **6(3)**.p192.

Sallusto F, Geginat JLanzavecchia A (2004) Central memory and effector memory T cell subsets: function, generation, and maintenance. Annu Rev Immunol **22**:745–763.

Shah, P., Vella, A., Basu, A., Basu, R., Adkins, A., Schwenk, W.F., Johnson, C.M., Nair, K.S., Jensen, M.D., Rizza, R.A, (2003). Elevated free fatty acids impair glucose metabolism in women: decreased stimulation of muscle glucose uptake and suppression of splanchnic glucose production during combined hyperinsulinemia and hyperglycemia. Diabetes **52**:38-42.

Skurk T, Kolb H, Muller-Scholze S, Rohrig K, Hauner H, Herder C (2005). The proatherogenic cytokine interleukin-18 is secreted by human adipocytes. Eur J Endocrinol, **152**:863-868.

Solinas G, Vilcu C, Neels JG, Bandyopadhyay GK, Luo JL, Naugler W, Grivennikov S, Wynshaw-Boris A, Scadeng M, Olefsky JM, Karin M. (2007). JNK1 in hematopoietically derived cells contributes to diet-induced inflammation and insulin resistance without affecting obesity. Cell Metab. **6**:386–397.

Takeda K, Tsutsui H, Yoshimoto T, Adachi O, Yoshida N & Kishi Tet al.(1998) Defective interferon production. Infect. Immun. 57, 590–595. NK cell activity and Th1 response in IL-18-deficient mice. Immunity,**8**:383–390.

The ADVANCE Collaborative Group, (2008). Intensive blood glucose control and vascular outcomes in patients with type 2 diabetes. N Engl J Med. **358**:2560–2572.

The Diabetes Control and Complications Trial Research Group (DCCT), (1996). The absence of a glycemic threshold for the development of long-term complications: the perspective of the Diabetres Control and Complications. Trial. Diabetes. **45**:1289–1298.

Thorand B, Kolb H, Baumert J, Koenig W, Chambless L, Meisinger C, et al.(2005). Elevated levels of Interleukin-18 predict the development of type 2 diabetes. Results from the MONICA/KORA Augsburg study, 1984–2002, Diabetes **54**: 2932–2938.

Tilg, H & Moschen, A.R. (2008). Inflammatory mechanisms in the regulation of insulin resistance. Mol. Med. **14**(3-4).pp222-231.

Tominaga K, Yoshimoto T, Torigoe K, Kurimoto M, Matsui K, Hada T, et al. (2000). IL-12 synergizes with IL-18 or IL-1beta for IFN-gamma production from human T cells. Int Immunol,**12**:151–60.

Tomura M, Zhou XY, Maruo S, Ahn HJ, Hamaoka T, Okamura H, et al.(1998). The critical role for IL-18 in the proliferation and activation of NK1.1+ CD3_ cells. J Immunol,**160**:4738–46.

Troseid T, Ingebjørg S and Arnesen H (2010). The role of interleukin-18 in the metabolic syndrome. Cardiovascular Diabetology 9: 11.

UK Prospective Diabetes Study Group. (1998). Tight blood pressure control and risk of macrovascular and microvascular complications in type 2 diabetes: (UKPDS 38). Br Med J. **317**:703–713.

Venkatachalam K, Prabhu SD, Reddy VS, Boylston WH, Valente AJ, Chandrasekar B. (2009). Neutralization of interleukin-18 ameliorates ischemia/reperfusion-induced myocardial injury. J Biol Chem, **284**:7853–65.

Welsh P, Woodward M, Rumley A, Macmahon S, Lowe GD,(2010). Does interleukin-18 or tumor necrosis factor-alpha have an independent association with the risk of coronary Cytokine **50** (1): 94-98**.**

Woldbaek PR, Sande JB, Strømme TA, Lunde PK, Djurovic S, Lyberg T, et al.(2005). Daily administration of interleukin-18 causes myocardial dysfunction in healthy mice. Am J Physiol Heart Circ Physiol, **289**:H708–714.

Wong CK, Ho AW, Tong PC, et al (2007). Aberrant activation profile of cytokines and mitogen-activated protein kinases in type 2 diabetic patients with nephropathy. *Clin Exp Immuno*;**149**:123–131

Yamagami H, Kitagawa K, Hoshi T, Furukado S, Hougaku H, Nagai Y and Hori, M (2005). Associations of serum IL-18 levels with carotid intima-media thickness. Arteriosclerosis, Thrombosis, and Vascular Biology, **25**: 1458-1462**.**

Yuen CM, Chiu CA, Chang LT, Liou CW, Lu CH, Youssef AA, et al. (2007). Level and value of interleukin-18 after acute ischemic stroke. Circ J,**71**:1691–6.

Zaccone P, Phillips J, Conget I, Cooke A, Nicoletti F, (2005) IL-18 binding protein fusion construct delays the development of diabetes in adoptive transfer and cyclophosphamide-induced diabetes in NOD mouse. Clin Immunol. **115**(1):74-9.

Zilverschoon GR, Tack CJ, Joosten LA, Kullberg BJ, van der Meer JW, Netea MG.(2008). Interleukin-18 resistance in patients with obesity and type 2 diabetes mellitus. Int J Obes. **32**(9):1407-14

Zirlik, A. et al. (2007). Interleukin-18, the Metabolic Syndrome, and Subclinical Atherosclerosis Results From the Dallas Heart Study Arterioscler Thromb Vasc Biol. **27**;2043-2049.

Zorilla, E., Sanchez-Alavez, M. et al. (2007). Interleukin-18 controls energy homeostasis by suppressing appetite and feed efficiency. PNAS. **104**. 11097–11102

APPENDICES

Appendix 1: Liver Safety Algorithms

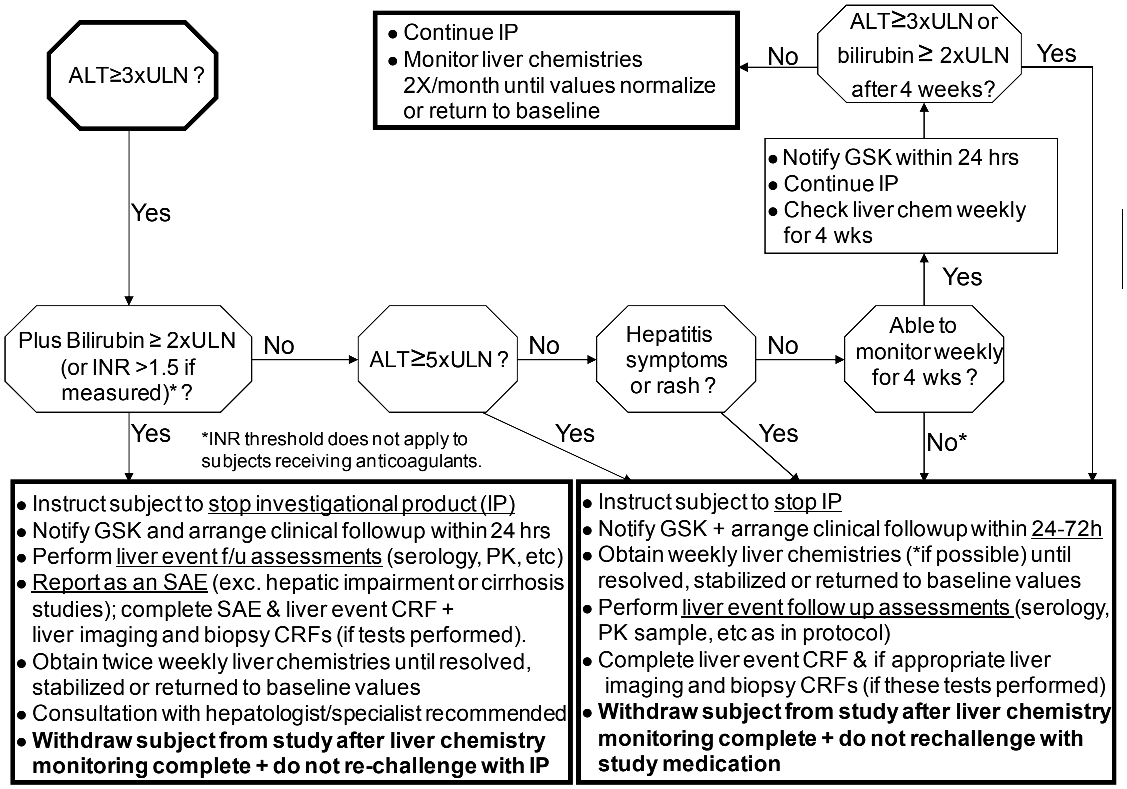

Appendix 2: Liver Safety Drug Restart Guidelines

Drug restart may be considered for a subject exhibiting compelling benefit for a critical medicine following drug-induced liver injury, if there is favorable benefit: risk ratio and no alternative medicine available.

Background Information on Drug Restart/Rechallenge

Following drug-induced liver injury, **drug restart or rechallenge is associated with a 13% mortality across all drugs in prospective studies.**[1] Clinical outcomes vary by drug, with nearly 50% fatality with halothane readministered in one month of initial injury. However, some drugs seldom result in recurrent liver injury or fatality. Risk factors for a fatal drug restart/rechallenge outcome include: hypersensitivity[1] with initial liver injury (e.g. fever, rash, eosinophilia), jaundice or bilirubin ≥2xULN or INR>1.5 suggesting severe liver injury, prior IP-related severe or fatal drug restart/rechallenge[2,3] or evidence of drug-related preclinical liability / mitochondrial impairment[3].

Drug Restart/Rechallenge Process (also see Figure 1)

1. Principal Investigator (PI) requests consideration of drug restart for a subject receiving compelling benefit from a critical or life-saving drug, who exhibits liver chemistry elevation meeting subject stopping criteria, with no alternative treatment.
2. GSK Medical Monitor & Clinical Safety Physician.to review the subject's restart/rechallenge risk factors & complete checklist (Table 4).

Table 4 Checklist for drug restart/Julie borlandrechallenge for critical medicine

(Following drug-induced liver injury, drug rechallenge is associated with 13% mortality across all drugs in prospective studies)		
	**Yes**	**No**
**Compelling benefit** of the investigational product (IP) **for this subject and no alternative therapy. Provide brief explanation**:		
**Relative benefit-risk favorable for drug restart/rechallenge**, after considering the **following high risk factors**:		
• Initial liver injury event included:		
– fever, rash, eosinophilia, or hypersensitivity		
– or bilirubin ≥2xULN (direct bilirubin >35% of total)		
• Subject currently exhibits ALT ≥3xULN, bilirubin ≥2xULN (direct bilirubin >35% of total, if available), or INR ≥1.5		
• Severe or fatal restart/rechallenge has earlier been observed with IP **If yes, please provide brief explanation**:		
• IP associated with known preclinical hepatic liability/ injury		

3. If GSK provides written approval for restart/rechallenge following the above review, the Principal Investigator (PI) must ensure the following:
    - The PI is to obtain Ethics Committee or Institutional Review Board review of drug reinitiation, as required.
    - PI must discuss the possible benefits and risks of drug reinitiation with the subject.
    - The subject must sign informed consent with a clear description of possible benefits and risks of drug administration, including recurrent liver injury or death. Consent specifically for the IP restart must be recorded in the study chart.
    - The drug must be reinitiated at GSK approved dose(s).
    - Subjects approved by GSK for restart of IP must return to the clinic twice a week for liver chemistry tests until stable, liver chemistries have been demonstrated and then laboratory monitoring may resume as per protocol. If protocol defined stopping criteria for liver chemistry elevations are met, study drug must be stopped.
    - The Ethics Committee or Institutional Review Board is to be informed of the subject's outcome, as required.
    - GSK is to be notified of any adverse events, as per Section 11.8

Figure 1 GSK process for drug restart after possible drug-induced liver injury

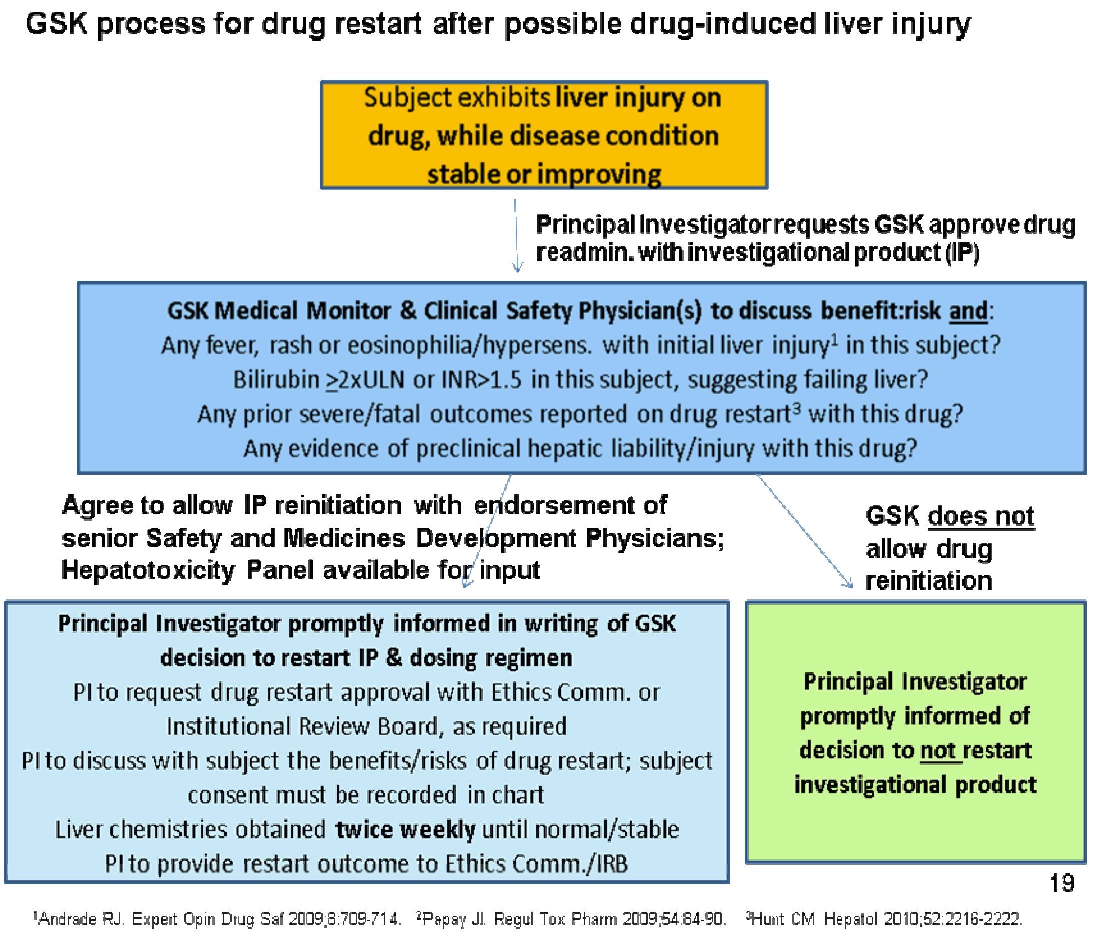

References:

1. Expert Opin Drug Saf 2009;8:709-714.
2. Papay JI. Regul Tox Pharm 2009;54:84-90.
3. Hepatol 2010;52:2216-2222

Appendix 3: Pharmacogenetic research

Pharmacogenetics - Background

Pharmacogenetics (PGx) is the study of variability in drug response due to hereditary factors in populations. There is increasing evidence that an individual's genetic composition (i.e., genotype) may impact the pharmacokinetics (absorption, distribution, metabolism, elimination), pharmacodynamics (relationship between concentrations and pharmacologic effects or the time course of pharmacologic effects) and/or clinical outcome (in terms of efficacy and/or safety and tolerability). Collection of whole blood samples, even when no a priori hypothesis has been identified, may enable PGx analysis to be conducted if at any time it appears that there is a potential unexpected or unexplained variation response to GSK1070806.

Pharmacogenetic Research Objectives

The objective of the PGx research (if there is a potential unexpected or unexplained variation) is to investigate a possible genetic relationship response to GSK1070806. If at any time it appears there is potential variability in response in this clinical study or in a series of clinical studies with GSK1070806 that may be attributable to genetic variations of subjects, the following objectives may be investigated – the relationship between genetic variants and study treatment with respect to:

- PK and PD of study treatment
- Safety and tolerability
- Efficacy

Informed Consent

Subjects who do not wish to participate in the PGx research may still participate in the clinical study. PGx informed consent must be obtained prior to any blood being taken for PGx research. Refusal to participate will involve no penalty or loss of benefits to which the subject would otherwise be entitled.

Study Population

Any subject who is enrolled in the clinical study can participate in PGx research. Any subject who has received an allogeneic bone marrow transplant must be excluded from the PGx research.

Subject participation in the PGx research is voluntary and refusal to participate will not indicate withdrawal from the clinical study.

Study Assessments and Procedures

Blood samples can be taken for Deoxyribonucleic acid (DNA) extraction and used in PGx assessments.

In addition to any blood samples taken for the clinical study, a whole blood sample (~10 ml) will be collected for the PGx research using a tube containing EDTA. It is recommended that the blood sample be taken at the first opportunity after a subject has been randomized and provided informed consent for PGx research, but may be taken at any time while the subject is participating in the clinical study.

No additional whole blood samples will be necessary for the PGx analysis. Saliva (2 ml) is spit into the DNA self-collection kit. A single sample will be taken but can be duplicated if the first sample is unusable. It is recommended that the saliva sample be taken at the first opportunity after a subject has been randomized and provided informed consent for PGx research, but may be taken at any time while the subject is participating in the clinical study.

The PGx sample is labelled (or "coded") with a study specific number that can be traced or linked back to the subject by the investigator or site staff. Coded samples do not carry personal identifiers (such as name or social security number). The blood sample is taken on a single occasion unless a duplicate sample is required due to inability to utilize the original sample.

The DNA extracted from the blood sample may be subjected to sample quality control analysis. This analysis will involve the genotyping of several genetic markers to confirm the integrity of individual samples. If inconsistencies are noted in the analysis, then those samples may be destroyed.

The need to conduct PGx analysis may be identified after a study (or a set of studies) of GSK1070806 has been completed and the clinical study data reviewed.

In some cases, the samples may not be studied. e.g., no questions are raised about how people respond to GSK1070806.

Samples will be stored securely and may be kept for up to 15 years after the last subject completes the study or GSK may destroy the samples sooner. GSK or those working with GSK (for example, other researchers) will use samples collected from the study for the purpose stated in this protocol and in the informed consent form.

Subjects can request their sample to be destroyed at any time.

Subject Withdrawal from Study

If a subject who has consented to participate in PGx research withdraws from the clinical study for any reason other than being lost to follow-up, the subject will be given a choice of one of the following options concerning the PGx sample, if already collected:

- Retain the sample for PGx research
- Destroy the PGx sample

If a subject withdraws consent from PGx research or requests sample destruction, the investigator must complete the appropriate documentation to request sample destruction within the timeframe specified by GSK, and maintain the documentation in the site study records.

Screen and Baseline Failures

If a blood sample for PGx research has been collected and it is then determined that the subject does not meet the entry criteria for participation in the clinical study, then the investigator should instruct the participant that their PGx sample will be discarded. No forms are required to complete this process as it will be completed as part of the consent and sample reconciliation process. In this instance a sample destruction form will not be available to include in the site files.

Pharmacogenetics Analyses

The need to conduct PGx analysis may be identified after a study (or set of studies) has been completed. For this reason, samples may be kept for up to 15 years after the last subject completes the study. GSK may destroy the samples sooner.

Generally, GSK will utilize one of two approaches to explore genetic variation in drug response.

1. Specific genes may be studied that encode the drug targets, or mechanism pathways, drug metabolizing enzymes, or which may underpin adverse events, disease risk or drug response. These candidate genes may include a common set of ADME (Absorption, Distribution, Metabolism and Excretion) genes that are studied to determine the relationship between gene variants or treatment response and/or tolerance.

   In addition, continuing research may identify other enzymes, transporters, proteins or receptors that may be involved in response to [insert the name of the study treatment]. The genes that may code for these proteins may also be studied.

2. Genome-wide scans involving a large number of polymorphic markers (e.g., single nucleotide polymorphisms) at defined locations in the genome, often correlated with a candidate gene, may be studied to determine the relationship between genetic variants and treatment response or tolerance. This approach is often employed when a definitive candidate gene(s) does not exist and/or the potential genetic effects are not well understood.

If applicable and PGx research is conducted, appropriate statistical analysis methods will be used to evaluate pharmacogenetic data in the context of the other clinical data. Results of PGx investigations will be reported either as part of the main clinical study report or as a separate report. Endpoints of interest from all comparisons will be descriptively and/or graphically summarized as appropriate to the data A detailed description of the analysis to be performed will be documented in the study reporting and analysis plan (RAP) analysis plan or in a separate pharmacogenetics RAP, as appropriate

Provision of Study Results and Confidentiality of Subject's PGx Data

GSK may summarize the PGx research results in the clinical study report or may publish the results in scientific journals.

GSK does not inform the investigator, subject, or anyone else (e.g., family members, study investigators, primary care physicians, insurers, or employers) of the PGx research results that are not known to be relevant to the subject's medical care at the time of the study, unless required by law. This is because the information generated from PGx studies is generally preliminary in nature, and therefore the significance and scientific validity of the results are undetermined.

Appendix 4: Hypoglycemia monitoring

There are often substantial differences in the interpretation and reporting of the severity of hypoglycemic episodes among investigators, studies, and clinical programs because of the diversity of the definitions used in clinical studies. One recommended approach for such standardization is to use classifications of severity from well-accepted sources, such as the ADA.

The ADA Workgroup on Hypoglycemia classifies hypoglycemia as follows (Diabetes Care, 2005, 28: 1245):

- Severe hypoglycemia. An event requiring assistance of another person to actively administer carbohydrate, glucagon, or other resuscitative actions. These episodes may be associated with sufficient neuroglycopenia to induce seizure or coma. Plasma glucose measurements may not be available during such an event, but neurological recovery attributable to the restoration of plasma glucose to normal is considered sufficient evidence that the event was induced by a low plasma glucose concentration.
- Documented symptomatic hypoglycemia. An event during which typical symptoms of hypoglycemia are accompanied by a measured plasma glucose concentration less than or equal to 70 mg/dL (3.9 mmol/L).
- Asymptomatic hypoglycemia. An event not accompanied by typical symptoms of hypoglycemia but with a measured plasma glucose concentration less than or equal to 70 mg/dL (3.9 mmol/L). Since the glycemic threshold for activation of glucagon and epinephrine secretion as glucose levels decline is normally 65 to 70 mg/dL (3.6 to 3.9 mmol/L) and since antecedent plasma glucose concentrations of less than or equal to 70 mg/dL (3.9 mmol/L) reduce sympathoadrenal responses to subsequent hypoglycemia, this criterion sets the lower limit for the variation in plasma glucose in nondiabetic, nonpregnant individuals as the conservative lower limit for individuals with diabetes.
- Probable symptomatic hypoglycemia. An event during which symptoms of hypoglycemia are not accompanied by a plasma glucose determination, but was presumably caused by a plasma glucose concentration less than or equal to 70 mg/dL (3.9 mmol/L). Since many people with diabetes choose to treat symptoms with oral carbohydrate without a test of plasma glucose, it is important to recognize these events as probable hypoglycemia. Such self-reported episodes that are not confirmed by a contemporaneous low plasma glucose determination may not be suitable outcome measures for clinical studies that are aimed at evaluating therapy, but they should be reported.

- Relative hypoglycemia. An event during which the person with diabetes reports any of the typical symptoms of hypoglycemia, and interprets the symptoms as indicative of hypoglycemia, but with a measured plasma glucose concentration greater than 70 mg/dL (3.9 mmol/L). This classification reflects the fact that patients with chronically poor glycemic control can experience symptoms of hypoglycemia at plasma glucose levels greater than 70 mg/dL (3.9 mmol/L) as plasma glucose concentrations decline toward that level. Though causing distress and interfering with the patient's sense of well-being, and potentially limiting the achievement of optimal glycemic control, such episodes probably pose no direct harm and, therefore, may not be a suitable outcome measure for clinical studies that are aimed at evaluating therapy, but they should be reported.

An electronic CRF page is provided for site investigators to document adverse events of hypoglycaemia in a manner consistent with the workgroup definitions.

Appendix 5: Protocol Amendment Changes

AMENDMENT 1

Where the Amendment Applies

This amendment applies to all the sites.

Summary of Amendment Changes with Rationale

To update the protocol to include interim data analyses for the purpose of internal GSK decision making.

List of Specific Changes

Section 5.3.1 Interim Analysis

PREVIOUS TEXT

Section 5.3.1 Data Review

A data review will be performed once the first 15 subjects have completed the study. The purpose of this review is to provide the project team and key GSK stakeholders with an early indication of any pharmacodynamic effects and/or safety, in order to plan future studies and will have minimal implications for altering the conduct of the study. It is not expected that this review will occur on a fully clean database.

Data summaries will be at the treatment group level only for key endpoints, and no individual subject data will be released. The circulation of results will be restricted to selected members of the project team and key GSK stakeholders (details to be included in the RAP, but in particular the results will not be circulated to staff involved in the conduct of the study or anyone who may be involved in data query resolution).

REVISED TEXT

~~Section 5.3.1 Data Review~~

~~A data review will be performed once the first 15 subjects have completed the study. The purpose of this review is to provide the project team and key GSK stakeholders with an early indication of any pharmacodynamic effects and/or safety, in order to plan future studies and will have minimal implications for altering the conduct of the study. It is not expected that this review will occur on a fully clean database.~~

~~Data summaries will be at the treatment group level only for key endpoints, and no individual subject data will be released. The circulation of results will be restricted to selected members of the project team and key GSK stakeholders (details to be included in the RAP, but in particular the results will not be circulated to staff involved in the conduct of the study or anyone who may be involved in data query resolution).~~

Section 5.3.1 Interim Analysis

During The Study

There will be ongoing data reviews conducted by the study team of the unblinded safety, pharmacokinetics and biomarker/pharmacodynamic data throughout the trial progression.

Interim Analysis: Day 57

Details of the formal interim analysis are outlined below:

- The interim analysis will occur when approximately 30 patients have completed Day 57 of the study. As appropriate, available data for subjects completing post Day 57 may also be included.
- The purpose of this interim analysis is to provide the project team and GSK stakeholders with key data to inform internal decision making and in order to plan future studies within the clinical development for the asset.
- Appropriate data summaries will be at the individual patient and treatment group level for key endpoints of interest and the circulation of results will be restricted to selected members of the project team and key GSK stakeholders. Results will not be circulated or discussed with staff involved in the conduct of the study at the sites.
- It is not expected that this review will occur on a fully clean database.
- There are no planned implications for the conduct of the study.
- Full details of planned interim analysis will be included in the reporting and analysis plan (RAP).

Interim Analysis: Day 85

It may also be necessary to conduct a formal interim analysis once all of the Day 85 data are available for all subjects to enable the data from the primary endpoint to be reported within 8 month of the primary completion date in accordance with GSK's data disclosure guidelines (POL-GSKF-408).

Full details of planned interim analysis will be included in the RAP.

Section 5.3.2 Final Analysis

PREVIOUS TEXT

Section 5.3.2 Interim Analysis

No formal interim analysis will be performed.

REVISED TEXT

~~Section 5.3.2 Interim Analysis~~

~~No formal interim analysis will be performed.~~

Section 5.3.2 Final Analysis
