# Supplementary material for: A Study to Investigate the Efficacy and Safety of an Anti-Interleukin-18 Monoclonal Antibody in the Treatment of Type 2 Diabetes Mellitus
Source: PLoS One. 2016 Mar 1;11(3):e0150018. doi: 10.1371/journal.pone.0150018 (PMC4773233; doi:10.1371/journal.pone.0150018)
Supplement: S1 Table — Note: Only results for Days 29, 57, and 85 are presented. (DOCX) [file pone.0150018.s012.docx]

Supplementary Tables

S1 Table. Summary of Statistical Analysis Results of Change from Baseline in Fasting Plasma Glucose (mmol/l) (All Visits up to Day 85) [Per Protocol Population].

| **Comparison** | **Day** | **Adjusted mean** | | **Adjusted difference (SE)**  **(GSK1070806–Placebo)** | **95% CI** |
| --- | --- | --- | --- | --- | --- |
|  |  | **GSK1070806** | **Placebo** |  |  |
| GSK1070806 0.25 mg/kg vs placebo | 29 | –0.49 | –0.02 | –0.46 (0.588) | (–1.66, 0.74) |
|  | 57 | –0.38 | –0.73 | 0.36 (0.708) | (–1.09, 1.80) |
|  | 85 | –0.02 | –0.53 | 0.51 (0.876) | (–1.28, 2.30) |
| GSK1070806  5 mg/kg vs placebo | 29 | –0.08 | –0.02 | –0.05 (0.585) | (–1.24, 1.14) |
|  | 57 | –1.07 | –0.73 | –0.33 (0.705) | (–1.78, 1.11) |
|  | 85 | –0.66 | –0.53 | –0.13 (0.874) | (–1.91, 1.66) |

Note: Only results for Days 29, 57 and 85 presented.
