# Supplementary material for: A Study to Investigate the Efficacy and Safety of an Anti-Interleukin-18 Monoclonal Antibody in the Treatment of Type 2 Diabetes Mellitus
Source: PLoS One. 2016 Mar 1;11(3):e0150018. doi: 10.1371/journal.pone.0150018 (PMC4773233; doi:10.1371/journal.pone.0150018)
Supplement: S2 Table — (DOCX) [file pone.0150018.s013.docx]

Supplementary Tables

**S2 Table. Summary of Statistical Analysis Results of Change from Baseline in Glucose-Weighted Mean AUC(0–4hrs) from Mixed Meal Test (All Visits up to Day 85) [Per Protocol Population].**

| **Comparison** | **Day** | **Adjusted mean** | | **Adjusted difference (SE)**  **(GSK1070806–Placebo)** | **95% CI** |
| --- | --- | --- | --- | --- | --- |
|  |  | **GSK1070806** | **Placebo** |  |  |
| GSK1070806 0.25 mg/kg vs placebo | 29 | –0.48 | 0.15 | –0.62 ( 0.667) | (–1.98, 0.74) |
|  | 57 | –0.40 | –0.41 | 0.01 ( 0.801) | (–1.62, 1.64) |
|  | 85 | –0.33 | –0.42 | 0.09 ( 0.847) | (–1.63, 1.82) |
| GSK1070806  5 mg/kg vs placebo | 29 | –0.44 | 0.15 | –0.58 ( 0.635) | (–1.88, 0.71) |
|  | 57 | –1.39 | –0.41 | –0.98 ( 0.774) | (–2.56, 0.60) |
|  | 85 | –1.11 | –0.42 | –0.68 ( 0.822) | (–2.36, 0.99) |
