# Supplementary material for: A Study to Investigate the Efficacy and Safety of an Anti-Interleukin-18 Monoclonal Antibody in the Treatment of Type 2 Diabetes Mellitus
Source: PLoS One. 2016 Mar 1;11(3):e0150018. doi: 10.1371/journal.pone.0150018 (PMC4773233; doi:10.1371/journal.pone.0150018)
Supplement: S3 Table — (DOCX) [file pone.0150018.s014.docx]

Supplementary Tables

**S3 Table. Summary of Statistical Analysis Results of Change from Baseline in Insulin-Weighted Mean AUC(0–4hrs) from Mixed Meal Test (All Visits up to Day 85) [Per Protocol Population].**

| **Comparison** | **Day** | **Adjusted mean** | | **Adjusted difference (SE)**  **(GSK1070806–Placebo)** | **95% CI** |
| --- | --- | --- | --- | --- | --- |
|  |  | **GSK1070806** | **Placebo** |  |  |
| GSK1070806 0.25 mg/kg vs placebo | 29 | –36.01 | 6.59 | –42.60 (16.868) | (–77.08, –8.11) |
|  | 57 | –17.71 | 8.81 | –26.52 (22.113) | (–71.64, 18.60) |
|  | 85 | 0.15 | 8.55 | –8.40 (26.090) | (–61.81, 45.02) |
| GSK1070806  5 mg/kg vs placebo | 29 | 11.53 | 6.59 | 4.94 (16.927) | (–29.66, 39.54) |
|  | 57 | 29.46 | 8.81 | 20.65 (22.158) | (–24.55, 65.86) |
|  | 85 | 11.86 | 8.55 | 3.31 (26.129) | (–50.18, 56.80) |
