# Supplementary material for: A Study to Investigate the Efficacy and Safety of an Anti-Interleukin-18 Monoclonal Antibody in the Treatment of Type 2 Diabetes Mellitus
Source: PLoS One. 2016 Mar 1;11(3):e0150018. doi: 10.1371/journal.pone.0150018 (PMC4773233; doi:10.1371/journal.pone.0150018)
Supplement: S4 Table — (DOCX) [file pone.0150018.s015.docx]

Supplementary Tables

**S4 Table. Summary of Statistical Analysis Results of Change from Baseline in C-peptide Weighted Mean AUC(0–4hrs) from Mixed Meal Test (All Visits up to Day 85) [Per Protocol Population].**

| **Comparison** | **Day** | **Adjusted mean** | | **Adjusted difference (SE)**  **(GSK1070806–Placebo)** | **95% CI** |
| --- | --- | --- | --- | --- | --- |
|  |  | **GSK1070806** | **Placebo** |  |  |
| GSK1070806 0.25 mg/kg vs placebo | 29 | –0.19 | 0.15 | –0.35 ( 0.167) | (–0.69, –0.01) |
|  | 57 | –0.15 | 0.06 | –0.21 ( 0.163) | (–0.54, 0.12) |
|  | 85 | 0.07 | 0.06 | 0.01 ( 0.155) | (–0.30, 0.33) |
| GSK1070806  5 mg/kg vs placebo | 29 | –0.02 | 0.15 | –0.17 ( 0.169) | (–0.51, 0.18) |
|  | 57 | –0.16 | 0.06 | –0.22 ( 0.166) | (–0.56, 0.12) |
|  | 85 | –0.09 | 0.06 | –0.15 ( 0.158) | (–0.47, 0.17) |
