# Supplementary material for: A Study to Investigate the Efficacy and Safety of an Anti-Interleukin-18 Monoclonal Antibody in the Treatment of Type 2 Diabetes Mellitus
Source: PLoS One. 2016 Mar 1;11(3):e0150018. doi: 10.1371/journal.pone.0150018 (PMC4773233; doi:10.1371/journal.pone.0150018)
Supplement: S5 Table — AE, adverse event. (DOCX) [file pone.0150018.s016.docx]

Supplementary Tables

S5 Table. All Post-Treatment AEs Reported in Two or More Patients.

| **AE** | **Placebo**  **(*n*=12)**  ***n* (%)** | **GSK1070806**  **(0.25 mg/kg)**  **(*n*=13)**  ***n* (%)** | **GSK1070806**  **(5 mg/kg)**  **(*n*=12)**  ***n* (%)** |
| --- | --- | --- | --- |
| **Any AE** | 11 (92) | 12 (92) | 11 (92) |
| Nasopharyngitis | 4 (33) | 3 (23) | 4 (33) |
| Diarrhoea | 2 (17) | 1 (8) | 2 (17) |
| Headache | 1 (8) | 3 (23) | 1 (8) |
| Hypertension | 0 | 2 (15) | 3 (25) |
| Back pain | 0 | 2 (15) | 2 (17) |
| Cough | 1 (8) | 1 (8) | 2 (17) |
| Arthralgia | 1 (8) | 0 | 2 (17) |
| Depression | 1 (8) | 1 (8) | 1 (8) |
| Dizziness | 0 | 3 (23) | 0 |
| Toothache | 2 (17) | 1 (8) | 0 |
| Urinary tract infection | 1 (8) | 1 (8) | 1 (8) |
| Asthenia | 0 | 1 (8) | 1 (8) |
| Constipation | 1 (8) | 0 | 1 (8) |
| Erythema | 0 | 2 (15) | 0 |
| Gastroenteritis | 0 | 1 (8) | 1 (8) |
| Paraesthesia | 1 (8) | 0 | 1 (8) |
| Pyrexia | 1 (8) | 1 (8) | 0 |

AE, adverse event
