# Supplementary material for: A Study to Investigate the Efficacy and Safety of an Anti-Interleukin-18 Monoclonal Antibody in the Treatment of Type 2 Diabetes Mellitus
Source: PLoS One. 2016 Mar 1;11(3):e0150018. doi: 10.1371/journal.pone.0150018 (PMC4773233; doi:10.1371/journal.pone.0150018)
Supplement: S6 Table — *Values represent the geometric mean (CVb%) for each parameter, except for Tmax: median (min–max). †AUC(0–Week 8) = area under the curve from time 0 to Week 8 following the second dose. ‡Cum AUC(0–Week 12) = cumulative area under the curve from time 0 to Week 12, from the first dose. N, number of patients in cohort; n, number of patients with nonmissing values; NA, not applicable. (DOCX) [file pone.0150018.s017.docx]

Supplementary Tables

S6 Table. Plasma GSK1070806 PK Parameters in the [Per Protocol Population].

| **PK parameter**^*^ | **GSK1070806 in mg/kg** | | | | | | | |
| --- | --- | --- | --- | --- | --- | --- | --- | --- |
|  | **0.25 mg/kg** | | | | **5 mg/kg** | | | |
|  | **Dose 1** | | **Dose 2** | | **Dose 1** | | **Dose 2** | |
|  | *N*=13 (*n*=11) | *N*=11 (*n*=11) | *N*=13 (*n*=13) | *N*=11  (*n*=11) | *N*=12 (*n*=12) | *N*=11  (*n*=11) | *N*=12 (*n*=12) | *N*=11  (*n*=11) |
| AUC(0-τ) (mg.hr/ml) | 1.84 (32.5) | 1.84 (32.5) | 2.48 (31.3) | 2.67 (27.4) | 25.4 (62.5) | 29.7 (22.4) | 44.3 (22.8) | 44.4 (23.9) |
|  | - | - | *N*=13 (*n*=13) | *N*=11  (*n*=11) | - | - | *N*=12 (*n*=12) | *N*=11  (*n*=11) |
| AUC(0-week 8) (mg.hr/ml)^†^ | NA | NA | 3.33 (29.7) | 3.55 (27.2) | NA | NA | 63.2 (23.1) | 63.2 (24.3) |
|  | - | - | *N*=13 (*n*=11) | *N*=11^*^ (*n*=11) | - | - | *N*=12 (*n*=12) | *N*=11  (*n*=11) |
| Cum AUC(0-week 12) (mg.hr/ml)^‡^ | NA | NA | 5.44 (25.4) | 5.44 (25.4) | NA | NA | 90.6 (24.1) | 92.9 (23.4) |
|  | *N*=13 (*n*=11) | *N*=11 (*n*=11) | *N*=13 (*n*=13) | *N*=11 (*n*=11) | *N*=12 (*n*=12) | *N*=11 (*n*=11) | *N*=12 (*n*=12) | *N*=11 (*n*=11) |
| C_max_ (µg/ml) | 8.36 (26.8) | 8.36 (26.8) | 9.69 (21.9) | 10.2 (17.2) | 95.2 (69.0) | 113 (23.2) | 153 (22.0) | 152 (23.0) |
|  | *N*=13 (*n*=11) | *N*=11 (*n*=11) | *N*=13 (*n*=13) | *N*=11 (*n*=11) | *N*=12 (*n*=12) | *N*=11 (*n*=11) | *N*=12 (*n*=12) | *N*=11 (*n*=11) |
| T_max_ (hours) | 1.1 (1-68) | 1.1 (1-68) | 1.1 (1-69) | 1.1 (1-69) | 1.1 (1-4) | 1.1 (1-4) | 1.1 (1-4) | 1.1 (1-4) |
|  | - | - | *N*=13 (*n*=12) | *N*=11 (*n*=10) | - | - | *N*=12 (*n*=12) | *N*=11 (*n*=11) |
| *t*_½_ (days) | NA | NA | 23.1 (22.7) | 23.3 (24.0) | NA | NA | 30.4 (21.3) | 30.3 (22.4) |

^*^Values represent the geometric mean (CVb%) for each parameter, except for T_max_: median (min-max)

^†^AUC(0–week 8) = area under the curve from time 0 to week 8 following the second dose.

^‡^Cum AUC(0–Week 12) = cumulative area under the curve from time 0 to week 12, from the first dose.

N, number of patients in cohort; n, number of patients with non-missing values; NA, not applicable
